# Overcoming GNA/RNA base-pairing limitations using isonucleotides improves the pharmacodynamic activity of ESC+ GalNAc-siRNAs

**DOI:** 10.1093/nar/gkab916

**Published:** 2021-10-14

**Authors:** Mark K Schlegel, Shigeo Matsuda, Christopher R Brown, Joel M Harp, Joseph D Barry, Daniel Berman, Adam Castoreno, Sally Schofield, John Szeto, Muthiah Manoharan, Klaus Charissé, Martin Egli, Martin A Maier

**Affiliations:** Alnylam Pharmaceuticals, Inc., Cambridge, MA 02142, USA; Alnylam Pharmaceuticals, Inc., Cambridge, MA 02142, USA; Alnylam Pharmaceuticals, Inc., Cambridge, MA 02142, USA; Department of Biochemistry, School of Medicine, Vanderbilt University, Nashville, TN 37232, USA; Alnylam Pharmaceuticals, Inc., Cambridge, MA 02142, USA; Alnylam Pharmaceuticals, Inc., Cambridge, MA 02142, USA; Alnylam Pharmaceuticals, Inc., Cambridge, MA 02142, USA; Alnylam Pharmaceuticals, Inc., Cambridge, MA 02142, USA; Alnylam Pharmaceuticals, Inc., Cambridge, MA 02142, USA; Alnylam Pharmaceuticals, Inc., Cambridge, MA 02142, USA; Alnylam Pharmaceuticals, Inc., Cambridge, MA 02142, USA; Department of Biochemistry, School of Medicine, Vanderbilt University, Nashville, TN 37232, USA; Alnylam Pharmaceuticals, Inc., Cambridge, MA 02142, USA

## Abstract

We recently reported that RNAi-mediated off-target effects are important drivers of the hepatotoxicity observed for a subset of GalNAc–siRNA conjugates in rodents, and that these findings could be mitigated by seed-pairing destabilization using a single GNA nucleotide placed within the seed region of the guide strand. Here, we report further investigation of the unique and poorly understood GNA/RNA cross-pairing behavior to better inform GNA-containing siRNA design. A reexamination of published GNA homoduplex crystal structures, along with a novel structure containing a single (*S*)-GNA-A residue in duplex RNA, indicated that GNA nucleotides universally adopt a rotated nucleobase orientation within all duplex contexts. Such an orientation strongly affects GNA-C and GNA-G but not GNA-A or GNA-T pairing in GNA/RNA heteroduplexes. Transposition of the hydrogen-bond donor/acceptor pairs using the novel (*S*)-GNA-isocytidine and -isoguanosine nucleotides could rescue productive base-pairing with the complementary G or C ribonucleotides, respectively. GalNAc-siRNAs containing these GNA isonucleotides showed an improved *in vitro* activity, a similar improvement in off-target profile, and maintained *in vivo* activity and guide strand liver levels more consistent with the parent siRNAs than those modified with isomeric GNA-C or -G, thereby expanding our toolbox for the design of siRNAs with minimized off-target activity.

## INTRODUCTION

Therapeutics based on RNA interference (RNAi) hold enormous potential for the treatment of a wide range of genetic diseases. RNAi utilizes a highly conserved mechanism by which double-stranded, small interfering RNA (siRNA), comprises lengths ranging from 20 to 25 nucleotides, load into the Argonaute 2 (Ago2) component of the RNA-induced silencing complex (RISC) and subsequently recognizes and degrades a target messenger RNA (mRNA). Despite the specificity afforded by full-length pairing to the intended target mRNA, unintended off-targets can arise through the binding of nucleotides from positions 2 to 8 in the seed region of the siRNA guide strand (g2-g8) with complementary site(s) in the 3′-untranslated region (3′-UTR) of mRNAs (1–4). Binding of these off-targets can lead to widespread transcriptional dysregulation through a microRNA (miRNA)-like mechanism. To enhance siRNA specificity by mitigating the miRNA-like repression of off-targets, thermally destabilizing modifications have been incorporated into the seed region of the guide strand (5–11). These chemical modifications preferentially diminish seed-only pairing (seed-pairing destabilization) while still allowing for productive full-length pairing and thereby maintain on-target activity. Of recent note, siRNAs conjugated to a triantennary *N*-acetylgalactosamine ligand [GalNAc-siRNAs, (12,13)] featuring full modification at the 2′-position of all ribonucleotides and a single incorporation of glycol nucleic acid (GNA) in the seed region of the guide strand have demonstrated an improved therapeutic index in rodents and initial proof of concept in human clinical trials (11, manuscript submitted). These GalNAc-siRNAs demonstrating an improved specificity and therapeutic index, designed in the context of our enhanced stabilization chemistry [ESC, (12,13)], were termed ESC+ (manuscript submitted).

GNA comprises an acyclic, three-carbon backbone with a single stereocenter and currently constitutes the simplest known nucleic acid self-pairing system with a phosphodiester backbone (14–16). Interestingly, homoduplexes of GNA have been shown to be thermodynamically more stable than duplexes of DNA or RNA of the same sequence (16). Although the enhanced stability of an acyclic nucleic acid analog such as GNA was initially counterintuitive, that stability was later attributed to a pre-organized single-stranded state, an enhanced base–base stacking within the zipper-like structure, and the ability of the GNA duplex to efficiently sample many different conformations of its backbone without disrupting Watson–Crick hydrogen bonding interactions between complementary nucleobases (17–19). Further evaluation of GNA crystal structures pointed towards a global structure of the (*S*)-isomer which closely mimics the backbone of an A-form helix, likely explaining the preference for cross-pairing with RNA but not DNA (17,18,20).

Despite the global similarities between GNA and RNA homoduplexes, one outstanding question from previous reports remained the uncharacteristically weak nature of (*S*)-GNA/RNA cross-pairing with increasing G:C content. Whereas the melting curves of GNA/RNA heteroduplexes consisting of only A and U/T nucleotides were indicative of stable duplex formation, an increase in the G:C content of these heteroduplexes by 1 or 3 base-pairs resulted in an unexpected decrease in the thermal stability and loss of a distinctive CD spectrum (16). We recently observed similar behavior where the incorporation of GNA-C or GNA-G into the center of double-stranded RNA resulted in a greater reduction of duplex stability relative to either GNA-A or GNA-T incorporation (21). Recent structural data provided some initial insight regarding the nature of this pairing discrepancy; a single GNA-T nucleotide adopted a unique rotated nucleobase orientation within an RNA duplex and paired with the complementary nucleotide in a reverse Watson–Crick fashion (21). Following this observation, we hypothesized that rotation of nucleobase orientation may be common to all GNA nucleotides when incorporated in duplex RNA and may explain the G:C versus A:T pairing discrepancy. This hypothesis was further supported by the demonstration of a reduced level of thermal destabilization after the incorporation of GNA-C or GNA-G in duplex RNA when paired with isoguanosine or isocytidine ribonucleotides, respectively, allowing for more productive hydrogen bond donor/acceptor interactions in a reverse Watson–Crick pairing mode (21–23).

Given the unanswered questions on the exact nature of GNA/RNA cross-pairing, especially in a G:C-rich sequence context, and the potential impact of a unique base-pairing which could limit the accessible sequence space for the development of GNA-containing siRNAs, we wanted to more fully understand GNA/RNA cross-pairing to better inform siRNA design in the context of our ESC+ approach. Herein, we further investigate the structural impact of GNA incorporation in RNA duplexes. We present new insights from previously reported GNA homoduplex structures and a novel duplex RNA crystal structure featuring a single (*S*)-GNA-A nucleotide, both of which demonstrate that a rotated nucleobase orientation is common to all GNA nucleotides regardless of duplex context. We describe the synthesis of two novel GNA monomers, (*S*)-GNA-isocytidine and (*S*)-GNA-isoguanosine (GNA-isoC and GNA-isoG, collectively GNA isonucleotides), which have an improved pairing ability with complementary nucleotides in RNA or siRNA conjugate duplexes. Finally, we show that GalNAc-siRNAs with GNA-isoC or GNA-isoG substitution are better tolerated *in vitro* relative to GNA-C or GNA-G, respectively, maintain the ability to reduce off-target effects, and lead to pharmacodynamic (PD) and pharmacokinetic (PK) profiles more consistent with the parent (not modified with GNA) siRNA in mice.

## MATERIALS AND METHODS

### Structure determination by X-ray crystallography

Crystals of the RNA dodecamer 5′-CGCG**A**A-^Br^U-UAGCG-3′ (site of GNA incorporation highlighted by the bold underlined text; ^Br^U = 5-bromo-Uridine) were grown by sitting-drop vapor-diffusion at room temperature. A crystal was obtained from a drop (0.8 μl) containing 0.5 mM oligonucleotide, 2.3 mM HoCl_3_, 40 mM NaCl, 5 mM spermine*4HCl, and 20 mM Na cacodylate, pH 7.0. Crystallization drops were equilibrated against reservoirs containing 70 μl 2-methyl-2,4-pentanediol. Crystals were mounted without further cryo-protection and flash-cooled in liquid nitrogen.

Diffraction data were collected on the 21-ID-D beam line of the Life Sciences Collaborative Access Team (LS-CAT) at the Advanced Photon Source (APS), located at Argonne National Laboratory (Argonne, IL). Crystals were maintained at 100 K during data collection. Diffraction images were collected using a Dectris Eiger 9 M hybrid photon counting detector. Data were collected using X-ray energy of 13 500 eV for Br-SAD phasing. Diffraction data were indexed, scaled and merged using the expert system, xia2 (24) and DIALS (25). Selected crystal data and data collection parameters are summarized in Supplementary Table S2.

SAD data were phased using SHELXC, SHELXD and SHELXE (26) through HKL2MAP (26,27). The resulting maps were used to manually build the model using COOT (28). The modified residues were built into the electron density and refinement continued with a dictionary created using PRODRG (29). All refinement was performed using the PHENIX package (30,31). Refinement parameters are summarized in Supplementary Table S2.

### Synthesis of (*S*)-GNA-isocytidine phosphoramidite

Compound **3**. To a solution of compound **1** (1.90 g, 17.1 mmol) in anhydrous DMF (34 ml) was added NaH (60% in mineral oil; 137 mg, 3.42 mmol). The reaction mixture was stirred at room temperature for 1 h and then a solution of compound **2** (5.85 g, 15.5 mmol) in DMF (34 ml) was added (15). The mixture was heated at 110°C for 18 h. After removing the solvent under reduced pressure, the residue was extracted with EtOAc and H_2_O. The organic layer was separated, dried over anhydrous Na_2_SO_4_, filtered and concentrated. The crude material was purified by flash column chromatography on silica gel (0–10% MeOH in CH_2_Cl_2_) to obtain compound **3** as a light-yellow foam (3.27 g, 6.71 mmol, 43%, *R*_f_ = 0.24 developed with 8% MeOH in CH_2_Cl_2_). ^1^H NMR (500 MHz, DMSO-*d*_6_): δ (ppm) = 7.45–7.43 (m, 2 H), 7.33–7.28 (m, 6 H), 7.22 (t, *J* = 7.3 Hz, 1 H), 7.16 (d, *J* = 7.5 Hz, 1 H), 6.90–6.88 (m, 4 H), 6.69 (brs, 2 H), 5.44 (t, *J* = 6.8 Hz, 2 H), 3.95–3.92 (m, 1 H), 3.83 (dd, *J* = 15.0, 3.5 Hz, 1 H), 3.74 (s, 6 H), 3.64 (dd, *J* = 14.8, 8.8 Hz, 1 H), 3.00 (dd, *J* = 9.5, 5.5 Hz, 1 H), 2.93 (dd, *J* = 9.5, 4.5 Hz, 1 H); ^13^C NMR (126 MHz, DMSO-*d*_6_): δ (ppm) = 169.98, 158.02, 155.45, 144.94, 143.95, 135.65, 135.60, 129.77, 127.76, 126.57, 113.13, 113.12, 105.88, 85.32, 67.50, 65.13, 55.01, 53.01; HRMS calc. for C_28_H_29_N_3_NaO_5_ [*M* + Na]^+^ 510.2005, found 510.1991.

Compound **4**. To a solution of compound **3** (3.25 g, 6.67 mmol) in MeOH (40 ml) was added *N,N*-dimethylformamide dimethyl acetal (1.77 ml, 13.3 mmol). The reaction mixture was stirred at room temperature for 16 h. After removing the solvent under reduced pressure, the residue was purified by flash column chromatography on silica gel (0–8% MeOH in CH_2_Cl_2_) to obtain compound **4** as a light-yellow foam (3.47 g, 6.39 mmol, 96%, *R*_f_ = 0.41 developed with 8% MeOH in CH_2_Cl_2_). ^1^H NMR (400 MHz, DMSO-*d*_6_): δ (ppm) = 8.59 (s, 1 H), 7.40 (d, *J* = 7.2 Hz, 2 H), 7.36 – 7.20 (m, 8H), 6.90 – 6.87 (m, 4 H), 5.59 (d, *J* = 7.6 Hz, 1 H), 5.17 (d, *J* = 6.0 Hz, 1 H), 4.43 (dd, *J* = 13.6, 3.6 Hz, 1 H), 3.97 (brs, 1 H), 3.74 (s, 6 H), 3.51 (dd, *J* = 13.4, 8.6 Hz, 1 H), 3.17 (s, 3 H), 3.02 – 2.97 (m, 1 H), 2.96 (s, 3 H), 2.90 – 2.86 (m, 1 H); ^13^C NMR (126 MHz, DMSO-*d*_6_): δ (ppm) = 170.59, 158.14, 158.10, 158.07, 144.99, 144.92, 135.63, 135.62, 129.72, 127.88, 127.68, 126.72, 113.20, 107.23, 85.39, 68.09, 65.94, 55.09, 54.10, 40.78, 34.69; HRMS calc. for C_31_H_35_N_4_O_5_ [*M* + H]^+^ 543.2607, found 543.2609.

Phosphoramidite ***i*C**. To a solution of compound **4** (2.00 g, 3.69 mmol) in CH_2_Cl_2_ (20 ml) and *N,N*-diisopropylethylamine (1.29 ml, 7.38 mmol) was added 2-cyanoethyl *N*,*N*-diisopropylchlorophosphoramidite **5** (1.32 ml, 5.90 mmol). The reaction mixture was stirred at room temperature for 16 h under an argon atmosphere. The reaction mixture was diluted with CH_2_Cl_2_ (200 ml) then washed with saturated aqueous NaHCO_3_ (100 ml). The organic layer was separated, dried over anhydrous Na_2_SO_4_, filtered and concentrated. The crude material was purified by flash column chromatography over silica gel (33–100% EtOAc in hexane then CH_2_Cl_2_:acetone:Et_3_N = 50:50:1) to obtain phosphoramidite ***i*C** as a light-yellow foam (880 mg, 1.18 mmol, 32%, *R*_f_ = 0.41 developed with 8% MeOH in CH_2_Cl_2_). ^1^H NMR (500 MHz, CD_3_CN): δ (ppm) = 8.64–8.63 (m, 1 H), 7.49–7.45 (m, 2 H), 7.35–7.19 (m, 8 H), 6.88–6.84 (m, 4 H), 5.67–5.61 (m, 1 H), 4.44–4.35 (m, 2 H), 3.92–3.83 (m, 1 H), 3.773–3.766 (m, 6 H), 3.73–3.52 (m, 4 H), 3.20–2.92 (m, 8 H), 2.60–2.43 (m, 2 H), 1.34–1.03 (m, 12 H); ^13^C NMR (126 MHz, CD_3_CN): δ (ppm) = 172.43, 172.32, 159.78, 159.69, 159.30, 146.12, 146.06, 145.64, 145.40, 136.94, 136.91, 136.84, 131.04, 131.01, 130.98, 130.96, 130.94, 129.01, 128.97, 128.95, 128.91, 128.87, 128.81, 127.84, 127.82, 114.16, 114.15, 114.03, 114.01, 108.75, 108.46, 87.00, 86.95, 72.84, 72.73, 72.36, 72.26, 70.02, 65.83, 65.44, 59.33, 59.18, 59.08, 58.92, 55.94, 55.92, 55.23, 54.53, 46.70, 44.07, 43.97, 43.87, 43.77, 41.80, 41.67, 35.63, 32.19, 29.73, 25.05, 25.01, 24.98, 24.95, 24.91, 24.89, 24.85, 24.84, 20.94, 20.89; ^31^P NMR (202 MHz, CD_3_CN): δ (ppm) = 150.28, 149.99; HRMS calc. for C_40_H_52_N_6_O_6_P [*M* + H]^+^ 743.3686, found 743.3690.

### Synthesis of (*S*)-GNA-isoguanosine phosphoramidite

Compound **7**. To a suspension of 2,6-diaminopurine **6** (9.38 g, 62.5 mmol) in anhydrous DMF (125 mL) was added NaH (60% in mineral oil; 500 mg, 12.5 mmol). The reaction mixture was stirred at room temperature for 1 h and then a solution of compound **2** (22.4 g, 59.5 mmol) in DMF (100 ml) was added. The mixture was heated at 110°C for 21 h. After removing the solvent under reduced pressure, the crude material was purified by flash column chromatography on silica gel (0–10% MeOH in CH_2_Cl_2_) to obtain compound **7** as a light-yellow foam (19.3 g, 36.6 mmol, 61%, *R*_f_ = 0.33 developed with 8% MeOH in CH_2_Cl_2_). ^1^H NMR (400 MHz, CDCl_3_): δ (ppm) = 7.41–7.39 (m, 3 H), 7.29–7.13 (m, 8 H), 6.81–6.79 (m, 4 H), 5.82 (brs, 2 H), 4.95 (brs, 2 H), 4.26–4.12 (m, 3 H), 3.77 (s, 6 H), 3.27 (dd, *J* = 9.2, 4.4 Hz, 1 H), 2.99 (dd, *J* = 9.6, 6.4 Hz, 1 H); ^13^C NMR (101 MHz, CDCl_3_): δ (ppm) = 158.68, 155.53, 151.90, 144.81, 139.54, 135.95, 135.84, 130.08, 128.13, 128.02, 127.02, 114.10, 113.32, 86.52, 69.82, 64.57, 55.36, 53.56, 48.50; HRMS calc. for C_29_H_31_N_6_O_4_ [*M* + H]^+^ 527.2407, found 527.2410.

Compound **8**. Compound **7** (19.0 g, 36.1 mmol) was treated with 80% aq. AcOH (500 ml) for 16 h. After removing the solvent, the residue was dissolved in toluene (200 ml), CH_2_Cl_2_ (100 ml) and MeOH (10 ml). The solution was left overnight at room temperature during which a white precipitate formed. The precipitated material was filtered and washed with CH_2_Cl_2_ to give the acetate salt of compound **8** as an off-white powder (8.68 g, 30.5 mmol, 84%, *R*_f_ = 0.18 developed with 20% MeOH in CH_2_Cl_2_). ^1^H NMR (400 MHz, D_2_O): δ (ppm) = 7.87 (s, 1 H), 4.24–4.20 (m, 1 H), 4.13–4.07 (m, 2 H), 3.68–3.57 (m, 2 H), 2.02 (s, 3H); ^13^C NMR (126 MHz, D_2_O): δ (ppm) = 179.54, 157.16, 153.94, 151.82, 142.16, 112.53, 70.38, 63.28, 46.54, 22.34; HRMS calc. for C_8_H_13_N_6_O_2_ [*M* + H]^+^ 225.1100, found 225.1099.

Compound **9**. To a suspension of compound **8** (7.88 g, 27.7 mmol) in H_2_O (250 ml) was added a solution of NaNO_2_ (7.41 g, 107.4 mmol) in H_2_O (47 ml) at 50°C. Then AcOH (11.1 ml, 193.9 mmol) was added dropwise. After stirring for 10 min, the brown colored solution was cooled on ice, diluted with H_2_O (250 ml) and concentrated aq. NH_4_OH (∼11 ml) was added to adjust the pH to 8. The solution was evaporated, and the residue was resuspended in H_2_O (250 ml). The resulting solid was filtered off and the cake was dried *in vacuo* overnight. The material was transferred to a round-bottom flask, coevaporated with toluene, and then dried *in vacuo* overnight to give the acetate salt of compound **9** as a light-purple solid (7.61 g, 26.7 mmol, 96%). ^1^H NMR (500 MHz, DMSO-*d*_6_): δ (ppm) = 7.62 (s, 1 H), 4.02 (dd, *J* = 14.0, 3.5 Hz, 1 H), 3.81 (dd, *J* = 14.0, 7.0 Hz, 1 H), 3.76–3.71 (m, 1 H), 3.34 (dd, *J* = 11.0, 5.0 Hz, 1 H), 3.21 (dd, *J* = 11.0, 6.5 Hz, 1 H), 1.83 (s, 3 H); ^13^C NMR (126 MHz, DMSO-*d*_6_): δ (ppm) = 174.55, 156.84, 152.44, 139.69, 112.74, 108.60, 69.67, 62.90, 45.63, 24.00; HRMS calc. for C_8_H_12_N_5_O_3_ [*M* + H]^+^ 226.0940, found 226.0938.

Compound **10**. To a suspension of compound **9** (3.03 g, 13.5 mmol) in MeOH (54 mL) was added *N,N*-dimethylformamide dimethyl acetal (3.57 mL, 26.9 mmol). The reaction mixture was stirred for 15 h at room temperature. Additional *N,N*-dimethylformamide dimethyl acetal (1.8 ml) and MeOH (20 ml) were added and the solution was heated at 55°C for 3 h. The mixture was evaporated and the residue was dried *in vacuo* overnight to give compound **10** as a grey powder (3.68 g, 13.1 mmol, 97%). ^1^H NMR (400 MHz, DMSO-*d*_6_): δ (ppm) = 10.97 (s, 1 H), 9.20 (s, 1 H), 7.78 (s, 1 H), 5.15 (brs, 1 H), 4.96 (brs, 1 H), 4.05 (dd, *J* = 13.4, 3.0 Hz, 1 H), 3.84–3.77 (m, 2 H), 3.38 – 3.34 (m, 1 H), 3.28–3.23 (m, 1 H), 3.20 (s, 3 H), 3.10 (s, 3 H); ^13^C NMR (126 MHz, DMSO-*d*_6_): δ (ppm) = 161.34, 157.89, 156.37, 154.24, 142.87, 69.42, 63.18, 45.65, 41.08, 34.29; HRMS calc. for C_11_H_17_N_6_O_3_ [*M* + H]^+^ 281.1362, found 281.1360.

Compound **11**. To a suspension of compound **10** (3.66 g, 13.1 mmol) in anhydrous pyridine (180 ml) and *N,N*-diisopropylethylamine (2.97 ml, 17.0 mmol) was added diphenylcarbamoyl chloride (3.04 g, 13.1 mmol). The reaction mixture was stirred at room temperature for 2 h then quenched with saturated aq. NaHCO_3_ (50 ml). The mixture was extracted with CH_2_Cl_2_ (300 ml), the organic layer separated, dried over anhydrous Na_2_SO_4_, filtered and concentrated. The crude material was purified by flash column chromatography on silica gel (0-8% MeOH in CH_2_Cl_2_) to give compound **11** as a brown foam (2.82 g, 5.93 mmol, 45%, *R*_f_ = 0.24 developed with 8% MeOH in CH_2_Cl_2_). ^1^H NMR (400 MHz, DMSO-*d*_6_): δ (ppm) = 8.93 (s, 1 H), 8.14 (s, 1 H), 7.44–7.28 (m, 10 H), 5.09 (d, *J* = 5.6 Hz, 1 H), 4.82 (t, *J* = 5.6 Hz, 1 H), 4.28 (dd, *J* = 14.0, 3.6 Hz, 1 H), 3.99 (dd, *J* = 14.0, 8.4 Hz, 1 H), 3.86 – 3.79 (m, 1 H), 3.43–3.30 (m, 2 H), 3.21 (s, 3 H), 3.13 (s, 3 H); ^13^C NMR (126 MHz, DMSO-*d*_6_): δ (ppm) = 160.31, 158.77, 155.26, 152.88, 151.58, 144.04, 141.94, 129.23, 127.10, 126.84, 123.38, 69.43, 63.57, 46.54, 40.79, 34.59; HRMS calc. for C_24_H_26_N_7_O_4_ [*M* + H]^+^ 476.2046, found 476.2040.

Compound **12**. To a solution of compound **11** (2.82 g, 5.93 mmol) in pyridine (30 mL) was added 4,4′-dimethoxytrityl chloride (2.21 g, 6.52 mmol) and the mixture was stirred at room temperature for 16 h. After quenching the reaction by the addition of MeOH (3 ml), the solvent was removed under reduced pressure. The residue was redissolved in CH_2_Cl_2_ (100 ml), washed with saturated aq. NaHCO_3_ (50 ml), the organic layer was separated, dried over anhydrous Na_2_SO_4_, filtered and concentrated. The crude was purified by flash column chromatography on silica gel (0–8% MeOH in CH_2_Cl_2_) to give compound **12** as a light-yellow foam (2.52 g, 3.24 mmol, 55%, *R*_f_ = 0.34 developed with 5% MeOH in CH_2_Cl_2_). ^1^H NMR (400 MHz, DMSO-*d*_6_): δ (ppm) = 8.91 (s, 1 H), 8.10 (s, 1 H), 7.43–7.37 (m, 10 H), 7.30–7.17 (m, 9 H), 6.85–6.82 (m, 4 H), 5.36 (d, *J* = 5.6 Hz, 1 H), 4.32 (dd, *J* = 13.6, 3.6 Hz, 1 H), 4.13–4.08 (m, 1 H), 4.05–4.01 (m, 1 H), 3.699 (s, 3 H), 3.697 (s, 3 H), 3.21 (s, 3 H), 3.13 (s, 3 H), 3.00 (dd, *J* = 9.6, 5.2 Hz, 1 H), 2.86 (dd, *J* = 9.4, 6.2 Hz, 1 H); ^13^C NMR (101 MHz, DMSO-*d*_6_): δ (ppm) = 160.29, 158.72, 158.00, 157.98, 155.30, 152.88, 151.63, 144.91, 143.83, 141.95, 135.55, 135.52, 129.73, 129.71, 129.22, 127.77, 127.70, 127.04, 126.80, 126.59, 123.35, 85.42, 67.80, 65.36, 54.97, 46.66, 40.80, 34.60; HRMS calc. for C_45_H_44_N_7_O_6_ [*M* + H]^+^ 778.3353, found 778.3345.

Phosphoramidite ***i*G**. To a solution of compound **12** (1.15 g, 1.48 mmol) in CH_2_Cl_2_ (15 ml) and *N,N*-diisopropylethylamine (1.03 ml, 5.92 mmol) was added 2-cyanoethyl *N*,*N*-diisopropylchlorophosphoramidite **5** (0.363 ml, 1.63 mmol) and 1-methylimidazole (0.118 ml, 1.48 mmol). The reaction mixture was stirred at room temperature for 1 h under an argon atmosphere. The reaction mixture was diluted with CH_2_Cl_2_ (200 ml), then washed with saturated aq. NaHCO_3_ (50 ml) and brine (50 ml). The organic layer was separated, dried over anhydrous Na_2_SO_4_, filtered and concentrated. The crude material was purified by flash column chromatography on silica gel (33–100% EtOAc in hexane) to obtain phosphoramidite ***i*G** as a light-yellow foam (1.16 g, 1.19 mmol, 80%, *R*_f_ = 0.30 developed with EtOAc). ^1^H NMR (500 MHz, CD_3_CN): δ (ppm) = 8.93 (s, 0.6 H), 8.91 (s, 0.4 H), 7.88 (s, 0.6 H), 7.83 (s, 0.4 H), 7.47–7.37 (m, 10 H), 7.31–7.18 (m, 9 H), 6.82–6.77 (m, 4 H), 4.41–4.33 (m, 3 H), 3.73 (s, 2H), 3.73 (s, 2.5 H), 3.73 (s, 3.5 H), 3.65–3.43 (m, 4 H), 3.19 (d, *J* = 2.5 Hz, 3 H), 3.16 (d, *J* = 2.0 Hz, 3 H), 3.14–2.95 (m, 2 H), 2.50–2.47 (m, 1 H), 2.42 (t, *J* = 4.8 Hz, 1 H), 1.08 (dd, *J* = 7.0, 5.5 Hz, 6 H), 0.98 (dd, *J* = 7.0, 3.0 Hz, 6 H); ^13^C NMR (126 MHz, CD_3_CN): δ (ppm) = 161.86, 161.83, 159.89, 159.82, 159.63, 159.62, 156.96, 156.92, 154.57, 154.43, 153.19, 153.16, 146.07, 146.02, 144.32, 144.18, 143.41, 136.78, 136.72, 136.68, 131.04, 130.99, 130.21, 130.20, 128.98, 128.94, 128.83, 128.82, 128.17, 127.88, 127.80, 127.78, 124.87, 124.77, 119.52, 119.33, 114.01, 87.12, 72.28, 72.26, 72.16, 72.15, 65.26, 65.23, 65.01, 64.99, 59.39, 59.32, 59.24, 59.17, 55.88, 47.15, 47.11, 46.80, 46.78, 43.97, 43.93, 43.87, 43.83, 41.69, 35.29, 24.94, 24.90, 24.88, 24.84, 24.82, 20.89, 20.85, 20.83, 20.79; ^31^P NMR (202 MHz, CD_3_CN): δ (ppm) = 150.65, 149.98; HRMS calc. for C_54_H_61_N_9_O_7_P [*M* + H]^+^ 978.4432, found 978.4434.

### Oligonucleotide synthesis

All oligonucleotides were prepared on a MerMade 192 synthesizer on a 1 μmole scale using universal or custom GalNAc supports. All phosphoramidites were used at a concentration of 100 mM in 100% Acetonitrile or 9:1 Acetonitrile:DMF with a standard protocol for 2-cyanoethyl phosphoramidites, except that the coupling time was extended to 400 s. Oxidation of the newly formed linkages was achieved using a solution of 50 mM I_2_ in 9:1 Acetonitrile:Water to create phosphate linkages or 100 mM DDTT (3-[(dimethylaminomethylene)amino]-3H-1,2,4-dithiazole-5-thione) in 9:1 Pyridine:Acetonitrile to create phosphorothioate linkages. After the trityl-off synthesis, columns were incubated with 150 μl of 40% aqueous methylamine for 30 minutes and the solution drained via vacuum into a 96-well plate. After repeating the incubation and draining with a fresh portion of aqueous methylamine (150 μl), the plate containing crude oligonucleotide solution was sealed and shaken at room temperature for an additional 60 min to completely remove all exocyclic and cyanoethyl protecting groups. For RNA-containing oligonucleotides, 200 μl of DMSO and 300 μl of triethylamine trihydrofluoride were added followed by heating to 60°C for 60 min to remove all silyl protecting groups. Precipitation of the crude oligonucleotides was accomplished via the addition of 1.2 ml of 9:1 acetonitrile:EtOH (conjugates) or 1:1 EtOH:iPrOH (RNA) to each well followed by centrifugation at 3000 RPM for 45 min, the supernatant removed from each well, and the pellets resuspended in 950 μl of 20 mM aqueous NaOAc. Oligonucleotides were purified using anion exchange chromatography (IEX) over a GE Source 15Q column (4.6 × 100 mm) with a linear gradient from 100 to 600 mM NaBr over 20 min in 20 mM sodium phosphate buffer (pH = 8.5) with 15% acetonitrile at 60°C. The desired fractions were desalted over a GE Hi-Trap Desalting Column (Sephadex G25 Superfine) using water to elute the final oligonucleotide products. All identities and purities were confirmed using ESI-MS and IEX HPLC, respectively.

### Determination of *T*_M_ via temperature-dependent UV spectroscopy

The melting studies were performed in 1 cm path length quartz cells on a Cary 300 spectrophotometer equipped with a thermoprogrammer. RNA 12-mer duplexes were evaluated at a duplex concentration of 2 μM in 1× PBS (10 mM Na/K phosphate buffer, pH 7.4, with 137 mM NaCl and 3 mM KCl). GalNAc-siRNA conjugate duplexes were evaluated at a duplex concentration of 1 μM in 0.1× PBS (1.0 mM Na/K phosphate buffer, pH 7.4, with 13.7 mM NaCl and 0.3 mM KCl). Each cuvette contained 800 μl of sample solution covered by 200 μl of light mineral oil. Melting curves were monitored at 260 nm with a heating rate of 1°C/min from 15-90°C. Melting temperatures (*T*_M_) were calculated from the first derivatives of the smoothed heating curves and the reported values are the result of at least two independent measurements.

### Evaluation of *in vitro* activity

Primary Mouse Hepatocytes (Thermo Fisher Scientific/Gibco) were transfected by adding 5.0 μl of a mixture containing Lipofectamine RNAiMax (Invitrogen, cat # 13778-150) and Opti-MEM plus (mixture composed of 0.1 μl of Lipofectamine and 5.0 μl of Opti-MEM plus) to each well, along with 5 μl of the desired siRNA duplex into a 384-well plate and incubated at room temperature for 15 min. About 40 μl of Dulbecco’s Modified Eagle Medium (Hep3b) or William’s Medium (PMH) containing ∼5 × 10^3^ cells were then added to the siRNA mixture. Cells were incubated for 24 h at 37°C and then processed for RNA purification. RNA was isolated using an automated protocol on a BioTek-EL406 platform using DYNABEADs (Invitrogen, cat # 61012). Briefly, 70 μl of Lysis/Binding Buffer and 10 μl of lysis buffer containing 3 μl of magnetic beads were added to each well. Plates were incubated on an electromagnetic shaker for 10 min at room temperature and then magnetic beads were captured and the supernatant was removed. Bead-bound RNA was then washed two times with 150 μl Wash Buffer A and once with Wash Buffer B. Beads were then washed with 150 μl Elution Buffer, re-captured and supernatant removed. 12 μl of a master mix containing 1.2 μl 10× Buffer, 0.48 μl 25× dNTPs, 1.2 μl 10× Random primers, 0.6 μl Reverse Transcriptase, 0.6 μl RNase inhibitor and 7.92 μl of water per reaction was added to RNA isolated above. Plates were sealed, mixed, and incubated on an electromagnetic shaker for 10 min at room temperature, followed by 2 h at 37°C. About 2 μl of cDNA were added to a master mix containing 2 μl water, 0.5 μl of either an appropriate GAPDH TaqMan VIC Probe or the target probe and 5 μl Lightcycler 480 probe master mix (Roche, cat # 04887301001) per well in a 384 well plate (Roche, cat # 04887301001). Real time PCR was done in a LightCycler480 Real Time PCR system (Roche). Each duplex was tested in quadruplicate and data were normalized to cells transfected with a non-targeting control siRNA. To calculate relative fold change, real time data were analyzed using the ΔΔ*C*t method and normalized to assays performed with cells transfected with a non-targeting control siRNA.

### Off-target reporter assays

Cos7 cells (ATCC, Manassas, VA) were grown to near confluence at 37°C in an atmosphere of 5% CO_2_ in DMEM (ATCC) supplemented with 10% FBS, before being released from the plate by trypsinization. siRNA and psiCHCECK2 plasmid transfection was carried out by adding 5 μl of siRNA duplexes and 5 μl of psiCHECK2 plasmid per well along with 5 μl of Opti-MEM plus 0.1 μl of Lipofectamine RNAiMax per well and then incubated at room temperature for 15 min. The mixture was then added to the cells which were resuspended in 35 μl of fresh complete media. The transfected cells were incubated at 37°C in an atmosphere of 5% CO_2_. 48 hours after the siRNAs and psiCHECK2 plasmid were transfected, Firefly (transfection control) and Renilla (fused to target sequence) luciferase were measured. First, media was removed from cells. Then, Firefly luciferase activity was measured by first adding 20 μl of Dual-Glo^®^ Luciferase Reagent equal to the culture medium volume to each well and mixed. The mixture was incubated at room temperature for 30 min before luminescence (500 nm) was measured on a Spectramax plate reader (Molecular Devices) to detect the Firefly luciferase signal. Renilla luciferase activity was measured by adding 20 μl of Dual-Glo® Stop & Glo^®^ Reagent to each well and the plates were incubated for 10–15 min before luminescence was again measured to determine the Renilla luciferase signal. siRNA activity was determined by normalizing the Renilla signal to the Firefly (control) signal within each well. The magnitude of siRNA activity was then assessed relative to cells that were transfected with the same vector but were not treated with siRNA or were treated with a non-targeting siRNA. All transfections were done at *n* = 4 or greater.

### Specificity evaluation of GalNAc-siRNAs

Transfection in PMH, incubation, and RNA extraction was performed as above. cDNA libraries were prepared with the TruSeq Stranded Total RNA Library Prep Kit (Illumina) and sequenced on a NextSeq500 sequencer (Illumina), all according to manufacturers’ instructions. Raw RNAseq reads were filtered with minimal mean quality scores of 28 and minimal remaining length of 36, using the ea-utils software fastq-mcf (https://expressionanalysis.github.io/ea-utils/). Filtered reads were aligned to the *mus musculus* genome (GRCm39/mm39) using STAR (ultrafast universal RNAseq aligner) (32). Uniquely aligned reads were counted by featureCounts (33) with the minimum mapping quality score set to 10. Differential gene expression analysis was performed by the R package DESeq2 with the betaPrior parameter set to TRUE to shrink log_2_ fold-change estimates for noisy, low-count genes (33,34).

### Care and use of laboratory animals

All procedures using mice were conducted by certified laboratory personnel using protocols consistent with local, state and federal regulations and in full compliance with AALAC guidelines at an AALAC-accredited facility. All procedures were approved by the Institutional Animal Care and Use Committee (IACUC) at Alnylam. All animals were acclimated in-house for 48 h prior to study start. Female C57BL/6 mice approximately 6–8 weeks of age were obtained from Charles River Laboratories and randomly assigned to each group. All animals were treated in accordance with IACUC protocols. Mice were dosed subcutaneously at 10 μl/g with siRNA duplex or phosphate buffered saline (PBS) control. GalNAc-siRNAs were diluted into PBS when making dosing solutions. All dosing solutions were stored at 4°C until 1 h before time of injection, when they were removed from storage and allowed to reach room temperature prior to injection. Animals were sacrificed at days indicated in the figures, after which livers were harvested and snap frozen for further analysis.

### Serum and plasma collection

Blood was collected utilizing the retro-orbital eye bleed procedure in accordance with IACUC approved protocols. For serum samples, blood was collected in Becton Dickinson serum separator tubes (Fisher Scientific, BD365967). Serum samples were kept at room temperature for 1 h and then spun in a micro-centrifuge at 21 000 × *g* at room temperature for 10 min. Serum was transferred to 96-well plates for storage at −80°C. For plasma samples, blood was collected in Becton Dickinson plasma (K_2_EDTA) separator tubes (Fisher Scientific, BD365974). Plasma samples were kept at 4°C for no >30 min before being spun in a micro-centrifuge at 10 000 × *g* at 4°C for 10 min. Plasma was transferred to 96-well plates for storage at −80°C.

### Quantification of circulating protein levels

TTR serum protein levels were measured by ELISA (serum was diluted 1:4000 and used in a mouse prealbumin kit, ALPCO, 41-PALMS-E01) following to the provided protocol. F12 plasma protein levels were measured by ELISA (plasma was diluted 1:20 000 and used in a mouse Factor 12 kit, Molecular Innovations, MFXIIKT-TOT) following to the provided protocol.

### *In vivo* gene expression evaluation

Powdered liver (∼10 mg) was resuspended in 500 μl QIAzol (RNeasy 96 Universal Tissue Kit, Qiagen, 74881) and a 5 mm steel grinding ball was added to each sample. Samples were homogenized at 25/s for 1 min at 4°C using a TissueLyser II (Qiagen, 85300). Samples were incubated at room temperature for 5 min followed by the addition of 100 μl chloroform. Samples were mixed by vigorously shaking the tubes, followed by a 10 min incubation at room temperature. Samples were spun at 12 000 × *g* for 15 min at 4°C and the supernatant was removed to a new tube and 1.5 volumes of 100% ethanol was added. Samples were then purified using a RNeasy 96 Universal Tissue Kit (Qiagen, 74881). Samples were eluted from RNeasy columns with 60 μl RNAse-free water (Ambion) and quantified on a Nanodrop (Thermo Fisher Scientific). About 1.5 μg of RNA was used to generate cDNA using a High-Capacity cDNA Reverse Transcription Kit (Applied Biosystems, 4368813). qPCR reactions were performed using gene specific TaqMan assays for each target (Mm00439249_m1 for *Hao1*) and mouse *Gapdh* as an endogenous control (Thermo Fisher, 4352339E). Real-Time PCR was performed in a Roche LightCycler 480 using LightCycler 480 Probes Master Mix (Roche, 04707494001). Data were analyzed using the ΔΔ*C*t method normalizing to control animals dosed with PBS alone.

### Quantification of total liver guide strand levels by RT-qPCR

Cohorts of mice were sacrificed on day 7 post-dose, and livers were snap frozen in liquid nitrogen and ground into powder for further analysis. Total liver guide strand levels were measured by stem-loop Taqman qPCR as previously described (35–37). RT-qPCR primers and probes used in this study are summarized in Supplementary Table S4.

### Mass identification of siRNA metabolites in mouse liver

Cohorts of mice were sacrificed on day 7 post-dose, and livers were snap frozen in liquid nitrogen and ground into powder for further analysis. Lyophilized mouse liver (50 mg) was thawed at RT to which 430 μl proteinase K digestion buffer (105 mM Tris-HCl, 17.5% Tween 20%, 1.26% Triton X-100, 50 mM CaCl_2_, 3 mM disodium EDTA, pH 8.0) was added. After briefly vortexing (20 s) and sonicating (10 min) at RT using a bath sonicator, 20 μl proteinase K (600 mAU/ml; Qiagen, Cat. 19133) was added and vortexed (5 s). After incubation for 3 hours at 50 degrees C, samples were centrifuged at 12.7 kRPM for 10 min and three aliquots of 100 μl supernatant was removed. To each fraction, 900 μl lysis loading buffer (Phenomenex, Cal. ALO-8579; adjusted to pH 5.5 with citric acid) with 0.5 ng/ml internal standard (12 nt fully modified 2′-O-methyl uridine oligonucleotide) was added. Solid phase extraction (SPE) was facilitated by an automated positive pressure manifold (Biotage, Extrahera) and Clarity OTX plates (Phenomenex, Cat. 8E-S103-EGA) per manufacturer’s recommendations. Briefly, the SPE plate was conditioned with 1 ml methanol and washed with 1.9 ml buffer (50 mM ammonium acetate, 2 mM sodium azide; pH 5.5). Samples were loaded (1 ml), washed 3× with 1.5 ml wash buffer (50 mM ammonium acetate in 50:40:10 H_2_O:MeCN:THF; pH 5.5) and eluted with 600 μl elution buffer (10 mM EDTA, 10 mM DTT, 100 mM ammonium bicarbonate, 50:40:10 H_2_O:MeCN:THF; pH 8.8). Solvent was evaporated to dryness using a nitrogen manifold (Biotage, Turbovap) at 40°C and 65 psi. Samples were reconstituted in 40 μl LC-MS grade water. Three replicate samples were combined and 30 μl was analyzed via high accuracy high resolution mass spectrometry (Thermo Scientific, QExactive) coupled to an Ultimate 3000 UPLC (Dionex). Chromatography was performed with a XBridge BEH XP C8 column (130 Å, 2.5 μm, 2.1 × 30 mm; Waters) at 80°C and a linear gradient of methanol (1–35%) in mobile phase A (16 mM triethylamine, 200 mM 1,1,1,3,3,3-hexafluoro-2-propanol in water). The mass spectrometer was equipped with a HESI II source and operated in negative ion full scan mode with a scan range of 500–2000 *m*/*z* at a resolution setting of 35 000. Spray voltage was 2.8 kV, auxiliary gas and capillary temperature were set to 300°C. Data analysis and signal deconvolution was performed using XCalibur software (Thermo Scientific) interfaced to Promass HR (Novatia LLC).

### Statistical analysis

Differences between group means relative to each other were evaluated for statistical significance using a one-way ANOVA in GraphPad Prism 8 and are indicated in each graph (n.s. = not significant, *P < 0.05, **P < 0.01, ***P < 0.001, ****P < 0.0001).

## RESULTS

### Evaluation of GNA and GNA/RNA mixed backbone crystal structures

**Figure 1.**
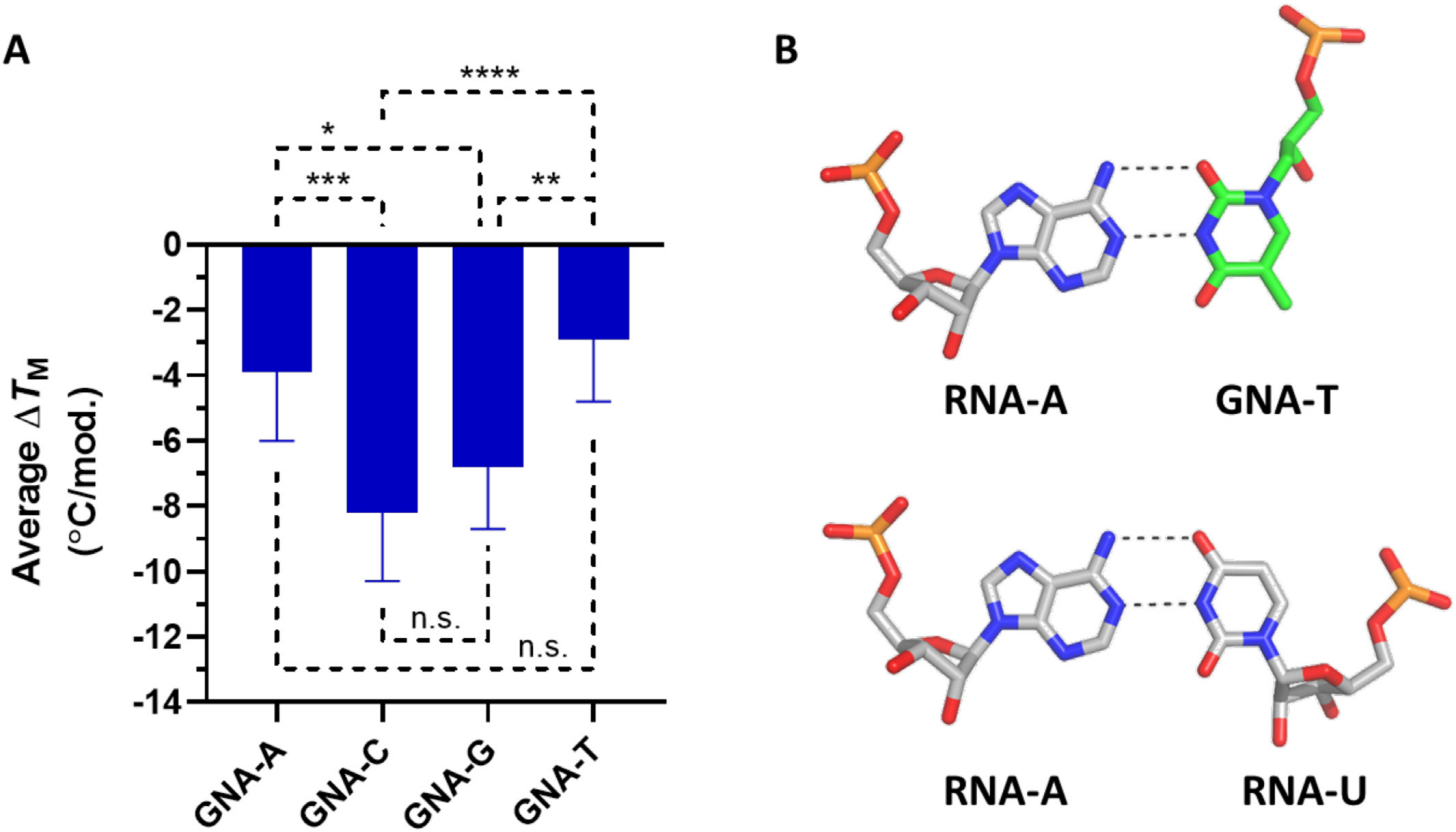
Reverse Watson–Crick base-pairing in GNA/RNA hetereoduplexes. (**A**) Average change in *T*_M_ after incorporation of a single GNA nucleotide in positions 3–19 in a GalNAc-siRNA conjugate (21). (**B**) Example of RNA-A:(*S*)-GNA-T (top) and RNA-A:RNA-U (bottom) base pairs demonstrating a rotated GNA nucleobase orientation in a reverse Watson–Crick base pair. Adapted from the structure 5V1L in the Protein Data Bank (21); *P < 0.05, **P < 0.01, ***P < 0.001, ****P < 0.0001.

All publications to date have reported that GNA homoduplexes exhibit Watson–Crick base pairing and that (*S*)-GNA/RNA heteroduplexes cross-pair in an antiparallel fashion within A:U-rich contexts (14,16,18). Despite these observations, we have previously shown that the incorporation of GNA-C or GNA-G into RNA or GalNAc-siRNA duplexes led to a greater thermal destabilization of the duplex compared to either GNA-A or GNA-T nucleotide incorporation (21). Further analysis of this *T*_M_ data indicated the statistical significance of those findings (Figure 1A). In crystal structures of self-complementary 8- and 12-mer RNA duplexes that contained a single (*S*)-GNA-T nucleotide, GNA-T and RNA-A were paired in a reverse Watson–Crick mode where the methyl group at C5 of the thymine nucleobase was directed into the minor groove (Figure 1B), providing a potential explanation of the unique melting behavior described above.

**Figure 2.**
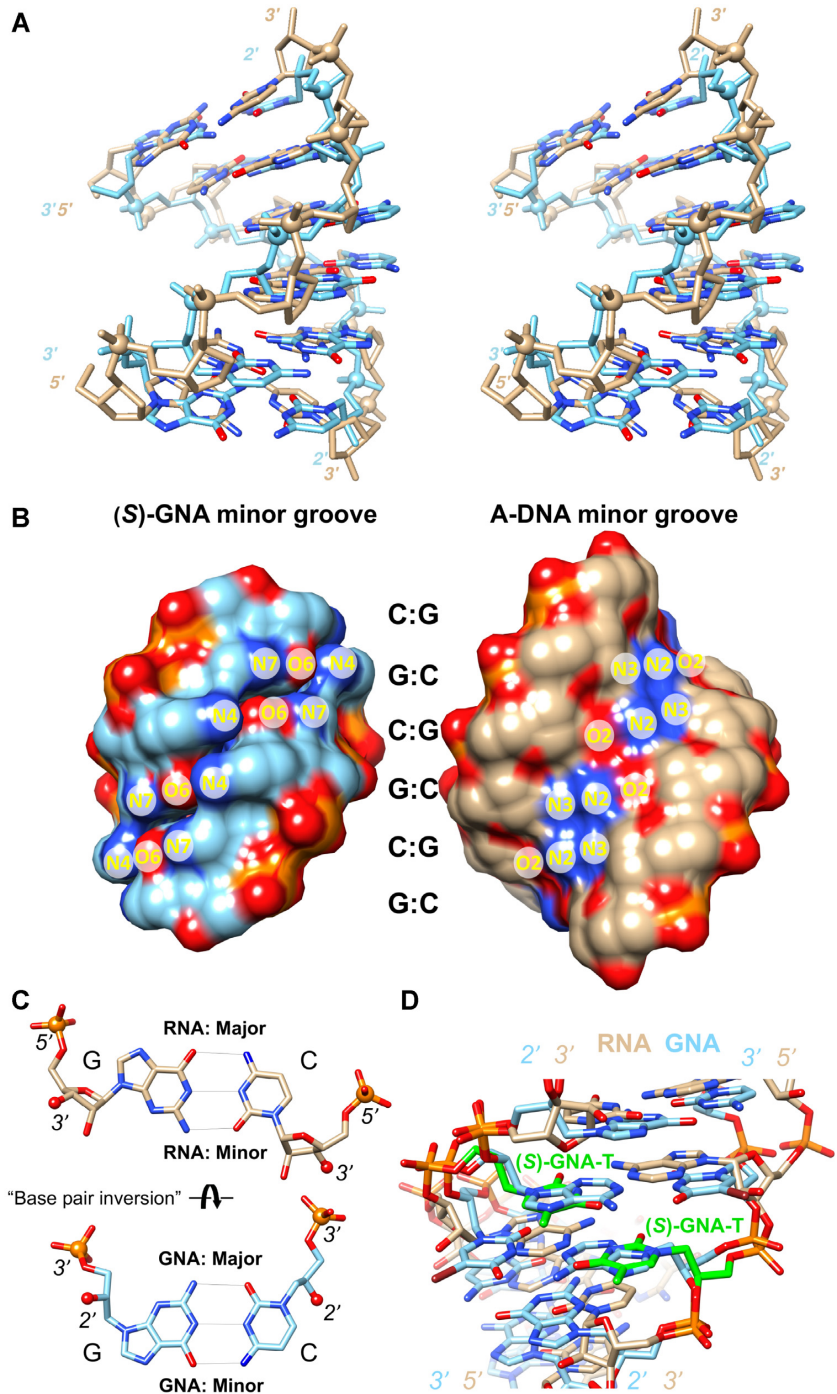
GNA displays an inverted base pair orientation relative to DNA and RNA. (**A**) Cross-eye stereo image of the (*S*)-GNA hexamer duplex (20) with sequence 3′-GCGCGC-2′ (light blue, PDB ID 2WNA) superimposed on the A-DNA model duplex with sequence 5′-GCGCGC-3′ (tan). Phosphorus atoms are highlighted as spheres and nucleobase oxygen and nitrogen atoms are colored in red and blue, respectively. The DNA duplex was built using the 3DNA program (38) and a 2.87 Å helical rise and a 33° twist. The overlay was done using phosphate groups and N1 (cytosine) and N9 (guanine) atoms, resulting in an r.m.s.d. of 2 Å. (**B**) The separated (*S*)-GNA and A-DNA duplexes from panel (A) in a surface rendering and viewed into the minor groove. Atom labels for guanine and cytosine H-bond donor and acceptor atoms indicate that base edges in the major and minor grooves are swapped in GNA relative to DNA and RNA. (**C**) Comparison between GNA and RNA G:C pairs that illustrates the rotation of base pairs around their long axis in the two systems, despite similar A-form backbone geometries and groove dimensions. RNA 5′-phosphorus and 3′-oxygen atoms as well as GNA 3′-phosphorus and 2′-oxygen atoms are drawn as spheres to highlight their similar relative orientations. (**D**) Overlay of the (*S*)-GNA octamer (19) duplex with sequence 3′-CTC-^Br^U-AGAG-2′ (PDB ID 2XC6) and the RNA duplex (21) of the sequence 5′-CGAA**T**UCG-3′ with (*S*)-GNA-T (green carbon atoms) modification (PDB ID 5V2H). The view is into the minor groove and illustrates that the GNA thymidine retains its base-rotated orientation opposite RNA adenosine.

To further understand the conformational behavior of GNA when incorporated in duplex RNA, and GNA pairing preferences in general, we revisited the structures of GNA homoduplexes (17,19–20). A comparison between hexameric A-form DNA (built using 3DNA program, 38) and (*S*)-GNA homoduplexes shows intriguingly similar, right-handed backbone curvatures, but a closer look reveals that the familiar major groove edges of G (O6 and N7) and C (N4) are directed into the minor groove in GNA (Figure 2A–C) (39). Similarly, the G (N2 and N3) and C (O2) minor groove edges are directed into the major groove in GNA. To our surprise, we realized that in over a decade of work directed at understanding the stability and pairing of this simplified nucleic acid system, the fact that GNA base-pairs were of the Watson–Crick type, but their orientation rotated 180° around the helical axis relative to the orientation in A- or B-form DNA and RNA had been overlooked. This rotation is distinct from that around the C1′-N1 (pyrimidines) or C1′-N9 (purines) glycosidic bond resulting in *syn* and *anti* nucleobase conformers in RNA or DNA. Rather, the structurally constrained backbone in GNA forces a change in the nucleobase orientation where the base is projected into the duplex from the side of the major instead of minor groove, resulting in the formation of reverse Watson–Crick pairs opposite RNA similar to what is observed in parallel duplexes of RNA or DNA. Not only are base pairs rotated between GNA and RNA or DNA, but GNA pairs are also shifted toward the minor groove (Figure 2D). This reorientation has important consequences. For one, the electrostatic surface potentials in the major and minor grooves of a GNA duplex will differ drastically from those in the corresponding grooves in RNA or DNA duplexes. Likewise, the arrangements of hydrogen bond donors and acceptors in GNA and RNA or DNA are very different (Figure 2B). Therefore, GNA constitutes a sort of hybrid between right-handed and left-handed DNA duplexes. Its backbone conformation resembles A-DNA, but the orientation of its base pairs resembles that in Z-DNA. In fact, the first reported CD spectra for (*S*)- and (*R*)-GNA homoduplexes offered telltale signs that GNA was not the simple pairing system it appeared to be (14). (*S*)-GNA duplexes show a broad negative CD peak with a minimum near 270 nm, almost the exact opposite of the broad positive bands in the CD spectra of A- and B-form DNA and A-RNA with maxima between 260 and 280 nm (40). The negative peak in the (*S*)-GNA spectra is quite similar to the negative peak characteristic of Z-DNA.

**Figure 3.**
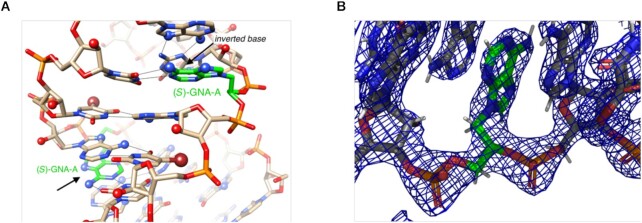
The RNA dodecamer duplex with single (*S*)-GNA-A residues (carbon atoms colored in green) viewed into the central minor groove. (**A**) The inverted orientation of GNA residues is indicated with arrows and all adenine N6 and N7 (blue), 2′-hydroxyl oxygen (red), and bromine atoms (brown) are highlighted in ball-and-stick mode. H-bonds are drawn with thin lines. (B) F_obs_ – F_calc_ electron density of the (*S*)-GNA-A residue demonstrating the unambiguous orientation of the nucleobase.

To expand upon our previous structural work on the behavior of pyrimidine GNA nucleotides ((*S*)-GNA-T) in RNA duplexes, we determined the crystal structure of 5′-CGCG**A**A-^Br^U-UAGCG-3′ (**A**= (*S*)-GNA-A; ^Br^U = 5-bromo-Uridine) at a resolution of 1.78 Å (Supplementary Table S2). This sequence was chosen since it has previously provided high quality structures (RNA with or without modified nucleotides) and resulted in the highest quality diffracting crystals of an RNA duplex containing GNA-A (21,41,42). The modified dodecamer crystallizes in space group *P*3_2_ with two independent duplexes per crystallographic asymmetric unit along with 156 water molecules. All four GNA-A residues pair with uridine in the reverse Watson–Crick mode. As shown in Figure 3, the adenine nucleobases of GNA project their N6 and N7 atoms into the minor groove and form two H-bonds with uridine: [gA]N6-H^…^O2[rU] and [gA]N1^…^H-N3[rU]. The structure confirms that GNA does not alter its hallmark inverted base orientation inside RNA and appears unable to adapt to the standard Watson–Crick pairing mode, thereby precluding the formation of three H-bonds in mixed GNA/RNA G:C pairs. Crystal structure determination of RNA duplexes containing GNA-C or GNA-G have so far been unsuccessful.

### Synthesis of (*S*)-GNA-isocytidine and (*S*)-GNA-isoguanosine phosphoramidites

**Figure 4.**
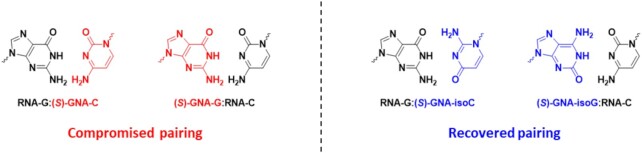
Proposed pairing modes of (*S*)-GNA-C, -G, -isoC and -isoG with complementary nucleotides in duplex RNA. The rotation of the GNA nucleobase orientation, if common to all four nucleotides, would allow full pairing of GNA isonucleotides in a reverse Watson–Crick pairing mode as shown on the right.

With accumulating structural evidence suggesting that both purine and pyrimidine nucleotides of GNA adopt a rotated nucleobase orientation in all oligonucleotide duplexes, we were interested in evaluating the isocytidine and isoguanosine nucleotides (22,23) of GNA alongside their native counterparts. Should rotation of the nucleobase be a common feature of all four nucleobases (A, C, G and T), one would expect an improved pairing ability of GNA-isoC and GNA-isoG nucleotides in a reverse Watson–Crick pairing mode relative to GNA-C and GNA-G, respectively (Figure 4).

**Scheme 1.**
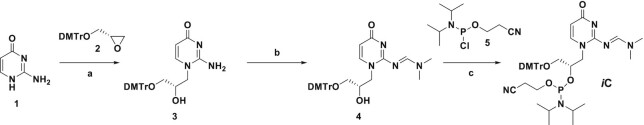
Synthesis of (*S*)-GNA-isocytidine phosphoramidite ***i*C**. (**a**) NaH, DMF, 43%; (**b**) DMF-DMA, MeOH, 96%; (**c**) DIPEA, CH_2_Cl_2_, 32%. DMF-DMA = *N*,*N*-dimethylformamide dimethylacetal; DIPEA = *N*,*N*-diisopropylethylamine.

To probe this structural observation more directly, we synthesized the phosphoramidite building blocks ***i*C** and ***i*G** for incorporation of (*S*)-GNA-isocytidine and (*S*)-GNA-isoguanosine into oligonucleotides, respectively. Commercially available isocytosine (**1**) was first used in the regio- and stereospecific ring-opening of (*S*)-glycidyl 4,4′-dimethoxytrityl ether **2** in DMF to afford compound **3** in 43% yield (Scheme 1). The *N*^2^-dimethylformamidine protecting group was subsequently introduced by heating a mixture of compound **3** and *N*,*N*-dimethylformamide dimethylacetal in methanol to afford compound **4** in 96% yield. Conversion of **4** to the desired phosphoramidite ***i*C** was accomplished using 2-cyanoethyl *N*,*N*-diisopropylchlorophosphoramidite (**5**) in dichloromethane and was isolated in 32% yield. Overall, phosphoramidite ***i*C** was isolated in 13% yield over three steps.

**Scheme 2.**
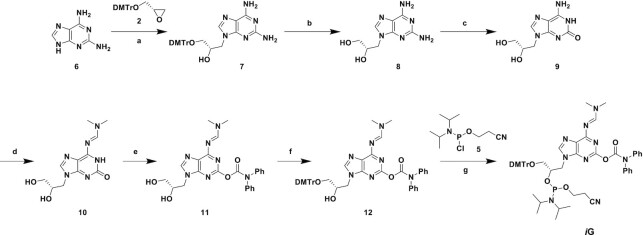
Synthesis of (*S*)-GNA-isoguanosine phosphoramidite ***i*G**. (**a**) NaH, DMF, 61%; (**b**) 80% aq. AcOH, 84%; (**c**) NaNO_2_, AcOH, H_2_O, 96%; (**d**) DMF-DMA, MeOH, 97%; (e) DPC-Cl, DIPEA, pyridine, 45%; (**f**) DMTr-Cl, pyridine, 55%; (**g**) DIPEA, MeIm, CH_2_Cl_2_, 80%. DMF-DMA = *N*,*N*-dimethylformamide dimethylacetal, DPC-Cl = diphenylcarbamoyl chloride, DMTr-Cl = 4,4′-dimethoxytrityl chloride, MeIm = 1-methylimidazole.

For the synthesis of the (*S*)-GNA-isoguanosine phosphoramidite ***i*G**, 2,6-diaminopurine (**6**) was utilized for the ring-opening of compound **2** in DMF to afford compound **7** in 61% yield (Scheme 2). Removal of the DMTr-protecting group was accomplished with 80% aqueous acetic acid to afford compound **8** in 84% yield. Subsequent regiospecific conversion of the *N*^2^-amine to oxygen through the diazonium salt was accomplished using NaNO_2_ in acetic acid to afford compound **9** in 96% yield (43). The *N*^6^-amine was protected using *N*,*N*-dimethylformamide dimethylacetal in methanol to afford compound **10** in 97% yield. Further protection of the *O*^2^-oxygen using diphenylcarbamoyl chloride in pyridine provided compound **11** in 45% yield and subsequent regiospecific installation of the DMTr-protecting group on the primary 3′-OH in pyridine afforded compound **12** in 55% yield. Finally, conversion of **12** to the desired phosphoramidite ***i*G** using 2-cyanoethyl *N*,*N*-diisopropylchlorophosphoramidite in CH_2_Cl_2_ was accomplished in 80% yield for an overall yield of 9% in seven steps.

### Impact of GNA isonucleotide incorporation on duplex thermal stability

**Figure 5.**
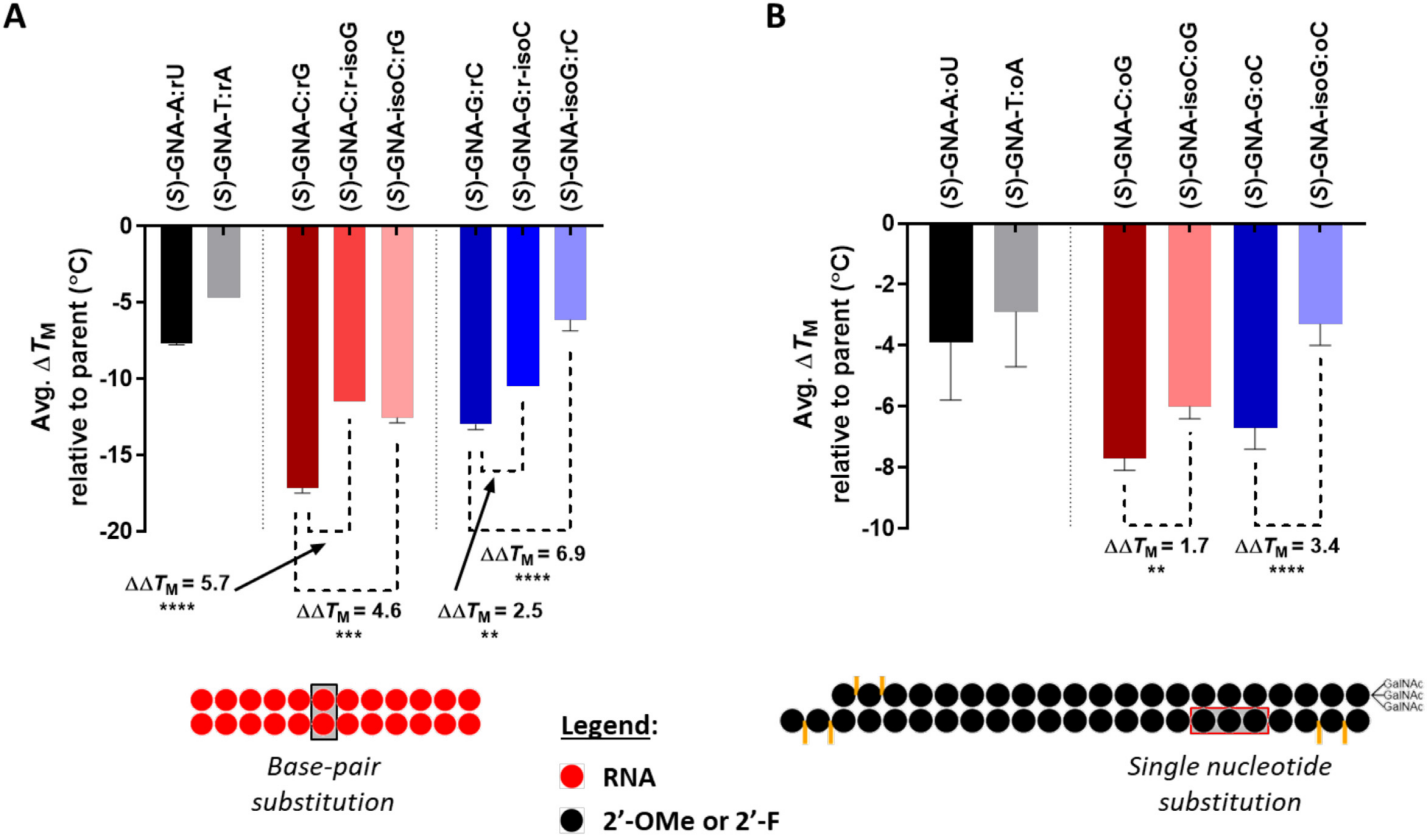
Impact of GNA nucleotide incorporation on duplex stability. (**A**) For the 12-mer RNA duplexes, the site of the modified base-pair is indicated by the black box (see Supplementary Table S1). Measurements were performed in 1× PBS at a duplex concentration of 2 μM and each data point is the average of two separate measurements relative to the unmodified parent. (**B**) The conjugate GalNAc-siRNA duplexes were modified with a single GNA nucleotide, and the site of incorporation is highlighted by the red box (g5, g6 or g7; see Supplementary Table S3). Measurements were performed on the fully modified GalNAc-siRNA duplexes (**D1**–**D28**) at a concentration of 1 μM in 0.1× PBS, and the data are represented as the average result across five different sequences relative to the parent GalNAc-siRNA not modified with GNA; *P < 0.05, **P < 0.01, ***P < 0.001, ****P < 0.0001.

The phosphoramidites ***i*C** and ***i*G** were used to incorporate a single (*S*)-GNA-isocytidine or (*S*)-GNA-isoguanosine nucleotide, respectively, into both native RNA and GalNAc-siRNA conjugate duplexes (Supplementary Tables S1 and S3). The melting temperature (*T*_M_) of duplexes containing these novel nucleotides was assessed using temperature-dependent UV spectroscopy. The incorporation of a single GNA-C destabilized the 12-mer RNA duplex by 17.2°C relative to the unmodified parent (Figure 5A). As previously reported, switching the complementary RNA-G nucleotide to RNA-isoG improved the pairing behavior and resulted in a significant lower destabilization of only 11.5°C (21). In this study, a comparable increase in stability relative to GNA-C was also observed when GNA-isoC was paired with RNA-G and a *T*_M_ that was lowered by only 12.6°C compared to the unmodified RNA duplex. The same RNA duplex featuring GNA-isoG paired with RNA-C showed a similar trend in which the measured destabilization of 6.2°C was reduced relative to the 13.0°C decrease in *T*_M_ observed with GNA-G:RNA-C, and even less than the 10.5°C destabilization for the GNA-G:RNA-isoC pair (Figure 5A).

We next evaluated the pairing ability of GNA-isoC and GNA-isoG with 2′-*O*-methyl (2′-OMe) nucleotides when incorporated into the seed region of the guide strand in several GalNAc-siRNA conjugates. The position of the modification ranged from g5-g7 depending on the sequence context (Supplementary Table S3). The substitution of GNA-C resulted in an average destabilization of 7.7°C across five sequences relative to the fully modified parent GalNAc-siRNA conjugate containing 2′-deoxy-2′-fluoro (2′-F) and 2′-OMe nucleotides (Figure 5B). In line with the results in the unmodified duplex RNA, GalNAc-siRNAs incorporating GNA-isoC had a measured *T*_M_ that was on average 6.0°C lower that the parent, representing an average increase in stability of 1.7°C compared to GNA-C. GalNAc-siRNAs containing a single GNA-G were destabilized by an average of 6.7°C across five sequences compared to their respective parent siRNA, whereas switching to GNA-isoG resulted in a significantly reduced *T*_M_ loss of 3.3°C, representing a stabilization of 3.4°C relative to GNA-G (Figure 5B).

### Impact of GNA isonucleotides on GalNAc-siRNA *in vitro* activity

**Figure 6.**
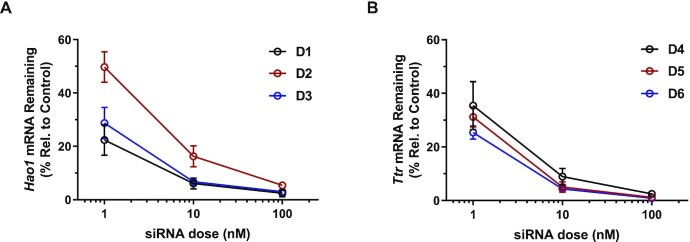
Free uptake silencing of parent and GNA-modified GalNAc-siRNAs (**A**) **D1**-**D3** targeting the mRNA of *Hao1* or (**B**) **D4**-**D6** targeting the mRNA of *Ttr* in primary mouse hepatocytes.

A subset of GalNAc-siRNAs used for *T*_M_ determination (Table 1) was used to evaluate the effect of GNA-isoC or GNA-isoG incorporation on free uptake *in vitro* silencing activity in primary mouse hepatocytes (PMH). Incorporation of GNA-C at g5 in **D2** targeting *Hao1* led to a decrease in on-target silencing relative to the parent **D1** at all three doses tested (Figure 6A). On the other hand, substitution of g5 with GNA-isoC in **D3** demonstrated silencing activity like the parent, albeit it with a slightly reduced activity at the lowest dose tested of 1 nM. We next evaluated GNA-isoG in comparison to GNA-G in an siRNA sequence targeting *Ttr*. Substitution of g6 with GNA-G in **D5** led to a similar level of *in vitro* activity relative to the parent **D4** at all doses tested (Figure 6B). Evaluation of the siRNA **D6** containing GNA-isoG demonstrated activity similar to the GNA-G modified **D5** but increased activity relative to the parent **D4**.

**Table 1.** GalNAc-siRNAs evaluated in mice.

siRNA Duplex	Target mRNA	Passenger (5′-3′) Guide (3′-5′)	*T*_M_ (°C)	Δ*T*_M_ (°C)	ΔΔ*T*_M_ (°C)
**D1**	*Hao1*	g•a•augu*G*aa*AG*ucaucgacaaL g•u•cuuac*A*c*U*uuca*GU*a*G*cug•*U*•u	66.5 ± 0.0	-	-
**D2**	*Hao1*	g•a•augu*G*aa*AG*ucaucgacaaL g•u•cuuac*A*c*U*uuca*GU*a*G***C**ug•*U*•u	58.6 ± 0.0	-7.9	-
**D3**	*Hao1*	g•a•augu*G*aa*AG*ucaucgacaaL g•u•cuuac*A*c*U*uuca*GU*a*Gi***C**ug•*U*•u	61.1 ± 0.0	-5.4	+2.5
**D4**	*Ttr*	a•a•cagu*G*u*UCU*ugcucuauaaL u•u•uuguc*A*c*A*agaacga*G*aua•*U*•u	66.0 ± 0.0	-	-
**D5**	*Ttr*	a•a•cagu*G*u*UCU*ugcucuauaaL u•u•uuguc*A*c*A*agaacga**G**aua•*U*•u	59.0 ± 0.0	-7.0	-
**D6**	*Ttr*	a•a•cagu*G*u*UCU*ugcucuauaaL u•u•uuguc*A*c*A*agaacga*i***G**aua•*U*•u	62.3 ± 0.0	-3.7	+3.3
**D7**	*F12*	u•g•cuuu*G*a*GCC*ucagcuucuaL u•c•acgaa*A*c*U*cggagucgaag•*A*•u	79.3 ± 0.4	-	-
**D8**	*F12*	u•g•cuuu*G*a*GCC*ucagcuucuaL u•c•acgaa*A*c*U*cggagu**C**gaag•*A*•u	71.3 ± 0.4	-8.0	-
**D9**	*F12*	u•g•cuuu*G*a*GCC*ucagcuucuaL u•c•acgaa*A*c*U*cggagu*i***C**gaag•*A*•u	73.3 ± 0.4	-6.0	+2.0
**D10**	*Ttr*	u•u•cuug*C*u*CUA*uaaaccguguL a•c•aagaa*C*g*A*gauauuuggca•*C*•a	70.0 ± 0.7	-	-
**D11**	*Ttr*	u•u•cuug*C*u*CUA*uaaaccguguL a•c•aagaa*C*g*A*gauauuug**G**ca•*C*•a	64.0 ± 0.7	-6.0	-
**D12**	*Ttr*	u•u•cuug*C*u*CUA*uaaaccguguL a•c•aagaa*C*g*A*gauauuug*i***G**ca•*C*•a	67.3 ± 0.4	-2.7	+3.3

Italicized uppercase, lower case and uppercase bold underlined letters represent 2′-F, 2′-OMe, and (*S*)-GNA modifications, respectively to adenosine, cytosine, guanosine and uridine. ‘L’ represents the tri-N-acetylgalactosamine ligand. Phosphorothioate linkages are indicated by the ‘•’ symbol. All *T*_M_ values are the average of two independent measurements of the fully modified siRNA duplex at a concentration of 1 μM in 0.1× PBS.

**Figure 7.**
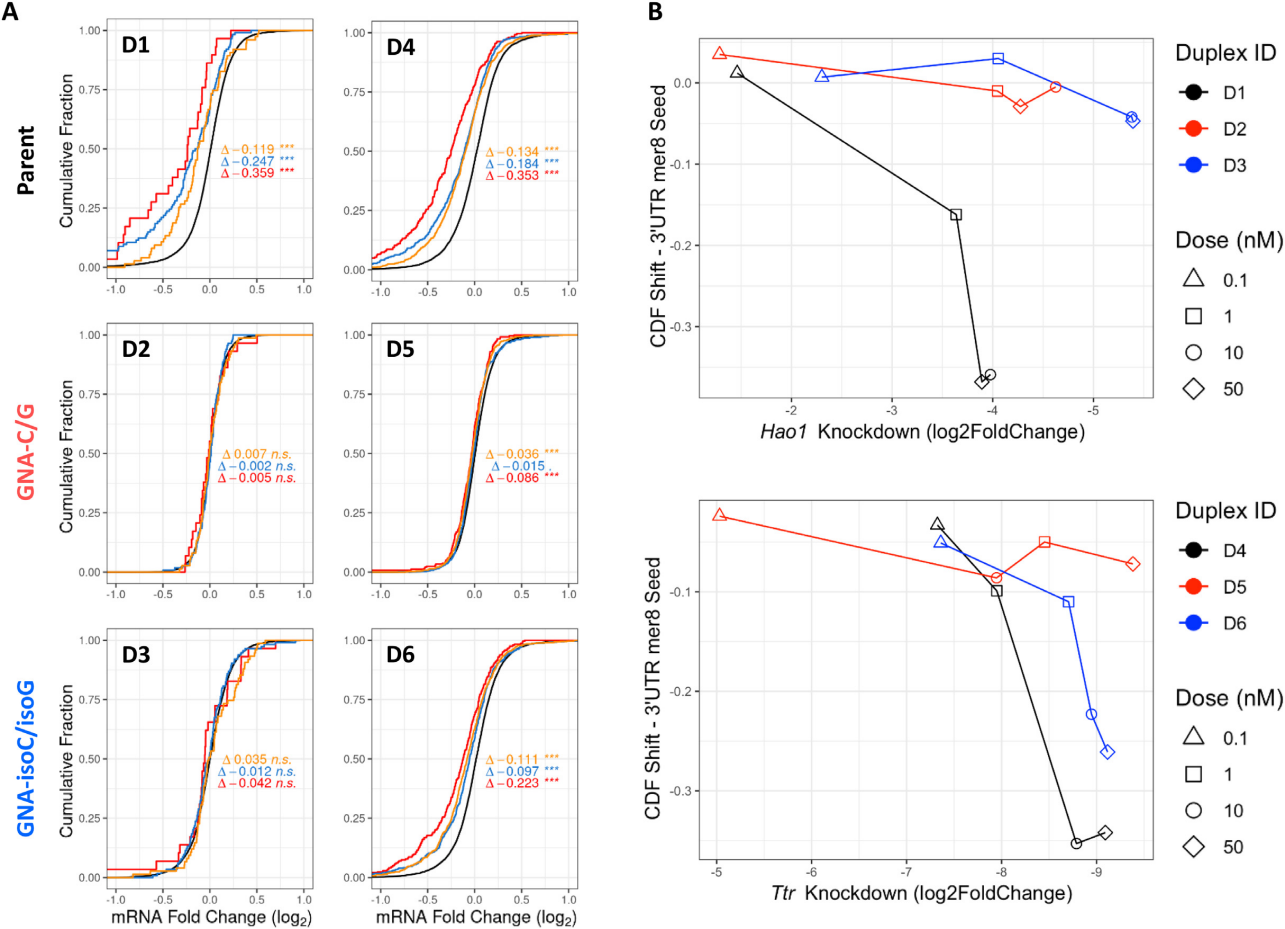
Evaluation of off- and on-target activity via RNA sequencing in primary mouse hepatocytes. (**A**) Cumulative distribution function (CDF) plots showing transcriptional dysregulation after transfection at a 10 nM dose of the indicated GalNAc-siRNAs. Each line represents the cumulative distribution of expression change among genes with or without the presence of the specified seed match in the 3′-UTR. The black line represents background genes lacking the specified seed matches below while the colored lines represent genes with at least one seed match, split by the identity of the strongest match (red = mer8, blue = mer7m8, yellow = mer7A1) (44). Delta values indicate the magnitude of each CDF shift versus background. (**B**) Plot of mer8 CDF shift versus on-target knockdown across various doses of the indicated GalNAc-siRNAs.

To evaluate the ability of GNA isonucleotide substitution to mitigate miRNA-like off-target repression, we utilized RNA sequencing to assess global transcriptional dysregulation after transfection of **D1**-**D6** at a dose of 0.1, 1, 10 or 50 nM in PMH. As shown in Figure 7A, parent **D1** or **D4** dosed at 10 nM in PMH led to a statistically significant downregulation in the cumulative expression of mRNA transcripts containing three different types of seed-matches (44) relative to background genes lacking those seed-matches (CDF shift). A similar pattern could be observed in plots of the log_2_-fold change of transcripts relative to a mock control (MA plots, Supplementary Figures S3 and S5). There was a strong correlation between the magnitude of the CDF shift and the on-target activity; the level of transcriptional dysregulation increased as the on-target activity increased for **D1** and **D4** (Figures 7B, Supplementary Figures S4 and S6, Supplementary Table S5). Transfection with the GNA-containing **D2** or **D5** did not lead to significant transcriptional dysregulation at any of the doses tested. In stark contrast to the parent siRNAs, the correlation between on-target activity and CDF shift was absent with **D2** and **D5**, demonstrating a strong reduction in the repression of off-targets with GNA-C and -G substitution. GNA-isoC substitution in **D3** led to a similar reduction in CDF shift across all doses tested. The correlation between on-target activity and CDF shift was also absent, suggesting that GNA-isoC substitution has a similar ability to mitigate miRNA-like off-targets like GNA-C. On the other hand, GNA-isoG substitution in **D6** led to a less pronounced reduction in CDF shift (Figure 7 and Supplementary Figure S6). Although there was a correlation between CDF shift and on-target activity similar to the parent **D4**, the shift of this curve to the right in Figure 7B indicated that substitution with GNA-isoG in **D6** did lead to lower levels of cumulative dysregulation than the parent when compared at the same level of on-target knockdown. These findings were largely corroborated using a dual-luciferase reporter assay expressing a four-tandem repeat complementary to the seed-region of the desired siRNA sequence (off-target reporter, Supplementary Figure S1). Although this is an idealized *in vitro* system, it was able to predict the trends observed in the RNA sequencing experiment when taking into account the differences in on-target free-uptake silencing across siRNAs in PMH (Supplementary Figure S2).

### Impact of GNA isonucleotides on GalNAc-siRNA activity in mice

**Figure 8.**
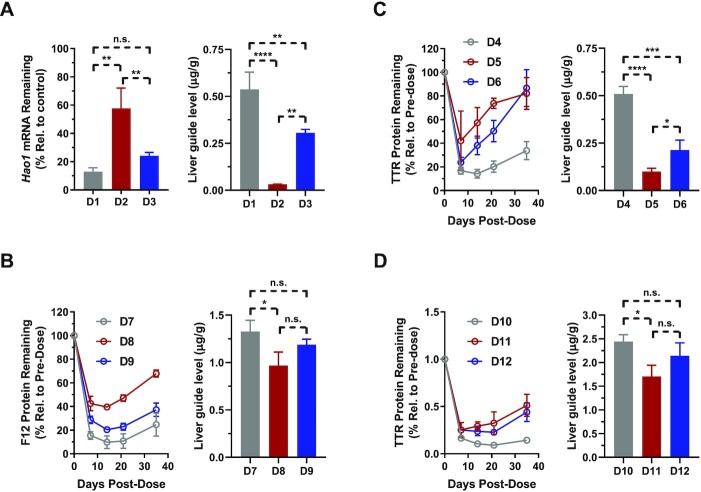
*In vivo* pharmacology (left of each panel) and guide liver levels (right of each panel) in mice after a single subcutaneous dose of parent or GNA-modified GalNAc-siRNAs. (**A**) *Hao1* mRNA knockdown 7 days after a dose of 1 mg/kg of **D1**-**D3**. (**B**) F12 protein knockdown after a dose of 1 mg/kg of **D7**-**D9**. (**C** and **D**) TTR protein knockdown after a dose of 0.5 mg/kg (**D4**-**D6**) or 1 mg/kg (**D10**-**D12**) siRNA; *P < 0.05, **P < 0.01, ***P < 0.001, ****P < 0.0001.

Given the strong body of supporting data suggesting that rotation of the nucleobase orientation is a common feature of all GNA nucleotides, we were interested in evaluating whether the improved base-pairing ability of GNA-isoC or GNA-isoG nucleotides could impact the *in vivo* translation of several GalNAc-siRNA conjugate duplexes. For the evaluation of GNA-isoC, two different siRNA sequences targeting either *Hao1* or *F12* were chosen based on previous data demonstrating a strong loss of activity in mice upon GNA-C substitution (Table 1). The incorporation of GNA-isoC at g5 (**D3**) of the *Hao1*-targeting sequence led to a similar, although slightly less efficacious, silencing of the target mRNA in the liver compared the parent siRNA (**D1**) lacking seed destabilization (Figure 8A). In comparison, the siRNA containing GNA-C at g5 (**D2**) was found to be significantly less efficacious with 42% compared to 87% target knockdown suggesting that GNA-C was less well tolerated *in vivo* than GNA-isoC. To further elucidate the observed differences in activity and the potential association with siRNA metabolic stability, we measured the levels of guide strand remaining in the liver for each of these GalNAc-siRNAs. Interestingly, the level of guide strand modified with GNA-C (**D2**) detected in the liver by RT-qPCR was found to be significantly lower compared to both the parent (**D1**) and GNA-isoC modified (**D3**) guide strands (Figure 8A). Furthermore, we utilized mass spectrometry to perform GalNAc-siRNA metabolite profiling from the livers of mice 7 days after a single dose of 10 mg/kg of **D1**-**D3** (Supplementary Figures S7–S9 and Supplementary Table S6). We observed that >85% of the detected metabolites corresponded to a guide strand that was truncated 5′- of the GNA-C incorporation in **D2** and were therefore considered inactive species. Only 13% of the active **D2** guide strand, defined as the sum of full-length and 3′-N-1 metabolite, was detected in the liver compared to a total of 97% for **D1**. Switching to GNA-isoC in **D3** substantially increased the level of active guide strand to 68%, but a significant amount of truncation 5′- of the GNA-isoC incorporation was still detected. The levels of guide strand measured using both RT-qPCR and mass spectrometry closely correlated with each other and the observed target knockdown in mice, suggesting that RT-qPCR may be utilized as a simple and efficient means of determining active guide liver levels (Figure 8A and Supplementary Figure S9).

Similarly, for a second sequence targeting *F12*, the GalNAc-siRNA incorporating GNA-isoC at g7 (**D9**) demonstrated a level and duration of F12 protein suppression that was like the parent siRNA (**D7**, Figure 8B). In contrast, the siRNA containing GNA-C at g7 (**D8**) was significantly less efficacious in F12 protein suppression relative to both the parent and GNA-isoC modified siRNAs. As with the previous set targeting *Hao1*, the level of guide strand detected in the liver correlated well with the on-target activity and a significantly lower amount of the GNA-C modified (**D8**), but not the GNA-isoC (**D9**) modified, guide strand detected relative to the parent (**D7**) seven days post-dose.

We next evaluated the activity of two different GalNAc-siRNA conjugate sequences containing GNA-isoG targeting *Ttr* in mice. The incorporation of GNA-G at g6 in **D5** led to a significant loss in both potency and duration of TTR protein suppression compared to the parent **D4** (Figure 8C). In contrast, the incorporation of GNA-isoG in **D6** was better tolerated with a similar efficacy at nadir (the timepoint at which maximum pharmacodynamic activity is observed), but shorter duration than the parent siRNA without seed destabilization. This activity data was again supported by the measured levels of each guide strand in the liver; whereas GNA-modified guide strands were detected at significantly lower levels than the parent **D4**, the level of GNA-isoG modified guide strand in **D6** was significantly higher than that of the GNA-G modified guide strand (**D5**, Figure 8C). In the second sequence targeting *Ttr*, the GNA-G (**D11**) or GNA-isoG (**D12**) modified siRNAs exhibited similar PD profiles with somewhat less efficacious and durable silencing compared to the parent siRNA **D10** (Figure 8D). Nevertheless, and similar to the previous examples, the differences in the levels of guide strands detected in the livers of the treated animals indicate that, in contrast to GNA-G (**D11**), the presence of GNA-isoG (**D12**) stabilized the siRNA against degradation to an extent similar to the parent (**D10**).

## DISCUSSION

We have recently reported an siRNA design strategy, termed ESC+, which has the potential to improve the therapeutic index of siRNAs with unfavorable off-target profiles in both rodents and humans through seed-pairing destabilization with GNA (manuscript submitted). Despite decades of previous research on GNA structure, the exact nature of GNA interactions in heteroduplexes with RNA has been poorly understood. Since the design of effective siRNAs utilizing GNA in an ESC+ approach relies on a duplex structure that can provide robust on-target activity and sufficient metabolic stability, we wanted to gain a better understanding of GNA/RNA heteroduplex structure to address any potential shortcomings of this approach and to further guide our siRNA design efforts. In this report we show that rotation of the base orientation in GNA nucleotides, first observed in an RNA duplex structure containing GNA-T, is common to all GNA nucleotides, whether present in GNA homoduplexes or GNA/RNA heteroduplex contexts. Although reverse Watson–Crick pairing does not have a large impact on cross-pairing of GNA-A or -T with complementary RNA nucleotides, it strongly disrupts the ability of GNA-C or -G to form a complementary base pair with RNA. A transposition of the hydrogen bond donor/acceptor pairs using the GNA isonucleotides of C and G demonstrated a significant improvement in the pairing ability of GNA with RNA, further resulting in an improved *in vitro* activity of GalNAc-siRNAs, similar off-target mitigation profiles as the isomeric counterparts, and a more consistent translation of the desired mRNA knockdown in mice.

Previous reports demonstrated that the (*S*)-isomer of GNA was capable of cross-pairing with RNA but not the (*R*)-isomer (16). While that behavior could be explained by the overall duplex conformation observed with GNA homoduplexes where the (*S*)-isomer adopted a structure comparable to an A-form like structure, the poor cross-pairing of (*S*)-GNA with RNA in G:C-rich contexts was not well understood. The newly uncovered and unique nucleobase orientation in GNA helps explain earlier puzzling observations regarding its pairing behavior. The right-handed and negatively inclined (*S*)-GNA strand (45,46) pairs stably with RNA, but does not tolerate G:C pairs because they feature only two H-bonds (reverse Watson–Crick) rather than three in the standard Watson–Crick pairing mode, are sheared, and result in less optimal stacking. As Figures 2D and 3 illustrate, GNA inside RNA retains its base orientation and so does RNA; hence the formation of reverse-Watson–Crick pairs. This likely complicates the pairing between (*S*)-GNA and DNA in addition to the shorter P-P distance (ca. 5.4 Å) in GNA compared to B-form DNA (ca. 7 Å). Moreover, B-form DNA may not adapt to the geometric constraints of the GNA backbone, unlike in its hybrids with RNA where it can convert to the A-form. Although (*S*)-GNA features inverted base pairs like Z-DNA, their backbone curvatures (right- and left-handed, respectively) do not match. Similarly, both (*R*)-GNA and Z-DNA are left-handed (smoothly curved phosphate backbone in the former and a zig-zag arrangement of phosphates in the latter), but they exhibit inverted base orientations. Finally, the shorter backbones of right-handed (*S*)-GNA and TNA should allow pairing, however, their base orientations are inverted so it is perhaps no surprise that 16-mer (S)-GNA and TNA strands that contained only A and T were not capable of cross-pairing (47). It would therefore appear that TNA and GNA are incapable of bridging the gaps that exist between them in terms of base orientation, backbone conformation, and perhaps dynamic behavior.

To directly probe the influence of a transposed hydrogen bonding donor/acceptor pair on GNA nucleotide pairing with RNA, the isonucleotides of GNA-C and -G were synthesized. Oligonucleotide synthesis proceeded with no changes to typical protocols and each nucleotide was successfully incorporated into both RNA and GalNAc-siRNA duplexes. GNA isonucleotides showed a significantly improved ability to cross-pair with complementary C or G nucleotides in both modified and unmodified RNA duplexes. This improved pairing presumably leads to an increased structural compatibility of the siRNA duplex with Ago2 during loading and/or the guide strand with target mRNA, thereby leading to an enhanced *in vitro* activity in primary mouse hepatocytes. A closer look at the ratio between off- and on-target activities through RNA sequencing demonstrated that ESC+ siRNAs containing GNA isonucleotides are capable of mitigating off-target effects, albeit in a reduced fashion with GNA-isoG. In the example shown with GNA-isoG, it may be that position g6 is not the ideal site of modification for the mitigation of off-targets as a sequence- and position-specific effect has been previously reported with GNA (11, manuscript submitted). Since it has been shown that the isocytidine and isoguanosine nucleobases can adopt various tautomeric forms (22,23), a further investigation into the impact of GNA-isoC and GNA-isoG substitution on pairing specificity and off-target mitigation across both position and sequence space is warranted.

A non-standard and strongly disruptive cross-pairing of GNA-C and -G with RNA has the potential to influence the *in vivo* translation of ESC+ GalNAc-siRNAs, thereby limiting the extent of this approach. Whereas GalNAc-siRNAs containing GNA-C or GNA-G can silence the intended target *in vitro* to a similar level as the parent siRNA lacking GNA, there were several instances in which the incorporation of these nucleotides led to a significant loss of *in vivo* activity. Given the enhanced structural perturbation afforded with a less than optimal pairing of GNA-C or -G with complementary RNA, the lack of activity in mice was hypothesized to be the result of a decreased metabolic stability of the siRNA *in vivo*, likely caused by an increased fraying of the duplex which can potentially expose the siRNA to a more expedient degradation by nucleases. The improved pharmacodynamics of the ESC+ GalNAc-siRNAs containing GNA-isoC or -isoG in mice was due to both an improved inherent activity, but also importantly to a decreased susceptibility to nucleolytic degradation as evidenced by the increased levels of intact guide strand detected in the liver by RT-qPCR or mass spectrometry.

In summary, these GNA isonucleotides expand our toolbox of modifications useful for seed-pairing destabilization of siRNAs. GNA-isoC and -isoG extend the utility of our ESC+ approach and will allow one to modulate the properties and further tailor each siRNA in support of a sequence-specific design of more effective and specific siRNAs using all GNA nucleotides (A, C, G, T, isoC, isoG). Our future work will focus on the further utilization of these novel GNA modifications in the context of our ESC+ design strategy, which offers the potential for increased sequence flexibility in the discovery of potent siRNAs with high specificity, *i.e*. low off-target potential.

## DATA AVAILABILITY

The RNA sequencing data in this manuscript has been deposited in NCBI’s Gene Expression Omnibus and are accessible through GEO Series accession number GSE183164.

Atomic coordinates and structure factors for the reported crystal structure have been deposited with the Protein Data bank under accession number 7LO9.

## Supplementary Material

gkab916_Supplemental_File
