# Supplementary material for: Overcoming GNA/RNA base-pairing limitations using isonucleotides improves the pharmacodynamic activity of ESC+ GalNAc-siRNAs
Source: Nucleic Acids Res. 2021 Oct 14;49(19):10851–67. doi: 10.1093/nar/gkab916 (PMC8565336; doi:10.1093/nar/gkab916)

Supporting Information

Overcoming GNA/RNA Base-Pairing Limitations Using Isonucleotides Improves the Pharmacodynamic Activity of ESC+ GalNAc-siRNAs

Mark K. Schlegel[1*], Shigeo Matsuda[1], Christopher R. Brown[1], Joel M. Harp[2], Joseph D. Barry[1], Daniel Berman[1], Adam Castoreno[1], Sally Schofield[1], John Szeto[1], Muthiah Manoharan[1], Klaus Charissé[1], Martin Egli[2], and Martin A. Maier[1]

[1] *Alnylam Pharmaceuticals, Inc., Cambridge, MA 02142, USA*
[2] *Department of Biochemistry, School of Medicine, Vanderbilt University, Nashville, TN 37232, USA*

**Corresponding author: mschlegel@alnylam.com*

Table of Contents

1. RNA sequences for T_M evaluation S2
2. X-ray diffraction data collection and statistics S3
3. Sequences of GalNAc-siRNAs S4
4. RT-qPCR primers and probes S6
5. Off-target silencing in dual-luciferase reporter and analysis S7
6. Additional plots and analyses from *in vitro* RNAseq S8
7. Mass Identification of siRNA Metabolites in Mouse Liver S13
8. NMR Spectra S16

RNA sequences for T_M evaluation

Table S1. RNA duplexes for the evaluation of thermal stability

Duplex	Sequence A (5' to 3')	Sequence B (5' to 3')	Avg. T_M	ΔT_M[a]	$\Delta\Delta T_M$
**R1**	UACAGUCUAUGU	ACAUAGACUGUA	53.1 ± 0.0	-	-
**R2**	UACAGUCUAUGU	ACAUAG**A**CUGUA		-7.7	-
**R3**	UACAG**T**CUAUGU	ACAUAGACUGUA		-4.7	-
**R4**	UACAGU**C**UAUGU	ACAUAGACUGUA	35.9 ± 0.3	-17.2	-
**R5**	UACAGU**C**UAUGU	ACAUA^{i}GACUGUA		-11.5	5.7
**R6**	UACAGU^{i}**C**UAUGU	ACAUAGACUGUA	40.6 ± 0.4	-12.6	4.6
**R7**	UACAGUCUAUGU	ACAUA**G**ACUGUA	40.1 ± 0.4	-13.0	-
**R8**	UACAGU^{i}CUAUGU	ACAUA**G**ACUGUA		-10.5	2.5
**R9**	UACAGUCUAUGU	ACAUA^{i}**G**ACUGUA	47.0 ± 0.7	-6.1	6.9

Uppercase bold underlined represent (*S*)-GNA modifications to Cytosine and Guanosine.
[a]ΔT_M data for **R2-R3**, **R5**, and **R8** were adapted from a previous publication where stabilities were measured under the same conditions, but on a different spectrophotometer (Schlegel *et. al.* JACS 2017).

X-ray diffraction data collection and statistics

Table S2. X-ray diffraction data collection and refinement statistics

**Parameter**	5'-CGCG**A**A-^{Br}U-UAGCG-3'
Wavelength [Å]	0.9184
Resolution range [Å]	23.76 - 1.78 (1.83 - 1.78)
Space group	$P3_2$
Unit cell [Å]; [°]	*a*=*b*=29.37, *c*=133.14; α=β=90, γ=120
Total reflections	330,087 (9,847)
Unique reflections	12,301 (1,274)
Multiplicity	26.7 (10.1)
Completeness (%)	99.61 (99.07)
Mean I/sigma(I)	17.8 (1.1)
Wilson B-factor [Å]	40.29
R-merge	0.082 (1.369)
R-meas	0.092 (1.544)
R-pim	0.017 (0.482)
CC1/2	0.999 (0.792)
Reflections used in refinement	12,298 (1,272)
Reflections used for R-free	556 (52)
R-work	0.1945 (0.4114)
R-free	0.2260 (0.4289)
Number of non-hydrogen atoms	1,024
macromolecules	852
ligands	156
solvent	16
RNA residues	48
RMS, bonds [Å]	0.002
RMS, angles [°]	0.46
Clashscore	2.67
Average B-factor [$Å^2$]	47.8

Numbers in parentheses refer to the highest resolution shell

Sequences of GalNAc-siRNAs

Table S3. siRNA duplexes used in these studies

siRNA	mRNA Target	Passenger	Sequence (5' to 3')	Guide	Sequence (5' to 3')	Avg. T_M	ΔT_M	$\Delta\Delta T_M$
D1	*Hao1*	P1	g●a●augu*G*aa*AG*ucaucgacaaL	G1	u●*U*●guc*G*a*UG*acuu*U*c*A*cauuc●u●g	66.5 ± 0.0	-	-
D2	*Hao1*	P1	g●a●augu*G*aa*AG*ucaucgacaaL	G2	u●*U*●gu**C***G*a*UG*acuu*U*c*A*cauuc●u●g	58.6 ± 0.1	-7.9	-
D3	*Hao1*	P1	g●a●augu*G*aa*AG*ucaucgacaaL	G3	u●*U*●gu[i]**C***G*a*UG*acuu*U*c*A*cauuc●u●g	61.1 ± 0.1	-5.4	2.5
D4	*Ttr*	P2	a●a●cagu*G*u*UCU*ugcucuauaaL	G4	u●*U*●aua*G*agcaaga*A*c*A*cuguu●u●u	66.0 ± 0.0	-	-
D5	*Ttr*	P2	a●a●cagu*G*u*UCU*ugcucuauaaL	G5	u●*U*●aua**G**agcaaga*A*c*A*cuguu●u●u	59.0 ± 0.0	-7.0	-
D6	*Ttr*	P2	a●a●cagu*G*u*UCU*ugcucuauaaL	G6	u●*U*●aua[i]**G**agcaaga*A*c*A*cuguu●u●u	62.3 ± 0.4	-3.7	3.3
D7	*F12*	P3	u●g●cuuu*G*a*GCC*ucagcuucuaL	G7	u●*A*●gaagcugaggc*U*c*A*aagca●c●u	79.3 ± 0.4	-	-
D8	*F12*	P3	u●g●cuuu*G*a*GCC*ucagcuucuaL	G8	u●*A*●gaag**C**ugaggc*U*c*A*aagca●c●u	71.3 ± 0.4	-8.0	-
D9	*F12*	P3	u●g●cuuu*G*a*GCC*ucagcuucuaL	G9	u●*A*●gaag[i]**C**ugaggc*U*c*A*aagca●c●u	73.3 ± 0.4	-6.0	2.0
D10	*Ttr*	P4	u●u●cuug*C*u*CUA*uaaaccguguL	G10	a●*C*●acgguuuauag*A*g*C*aagaa●c●a	70.0 ± 0.7	-	-
D11	*Ttr*	P4	u●u●cuug*C*u*CUA*uaaaccguguL	G11	a●*C*●ac**G**guuuauag*A*g*C*aagaa●c●a	64.0 ± 0.7	-6.0	-
D12	*Ttr*	P4	u●u●cuug*C*u*CUA*uaaaccguguL	G12	a●*C*●ac[i]**G**guuuauag*A*g*C*aagaa●c●a	67.3 ± 0.4	-2.7	3.3
D13	*Ttr*	P4	u●u●cuug*C*u*CUA*uaaaccguguL	G13	a●*C*●acg**G**uuuauag*A*g*C*aagaa●c●a	63.3 ± 0.4	-6.7	-
D14	*Ttr*	P4	u●u●cuug*C*u*CUA*uaaaccguguL	G14	a●*C*●acg[i]**G**uuuauag*A*g*C*aagaa●c●a	67.3 ± 0.4	-2.7	4.0
D15	*Ttr*	P5	a●g●gauc*U*u*GCC*aaagcaguaaL	G15	u●*U*●acugcuuuggc*A*a*G*auccu●g●g	74.3 ± 0.4	-	-
D16	*Ttr*	P5	a●g●gauc*U*u*GCC*aaagcaguaaL	G16	u●*U*●acu**G**cuuuggc*A*a*G*auccu●g●g	66.8 ± 0.4	-7.5	-
D17	*Ttr*	P5	a●g●gauc*U*u*GCC*aaagcaguaaL	G17	u●*U*●acu[i]**G**cuuuggc*A*a*G*auccu●g●g	70.0 ± 0.0	-4.3	2.8

D18	*Ttr*	P5	a●g●gauc*U*u*GCC*aaagcaguaaL	G18	u●*U*●acug**C**uuuggc*A*a*G*auccu●g●g	66.8 ± 0.4	-7.5	-
D19	*Ttr*	P5	a●g●gauc*U*u*GCC*aaagcaguaaL	G19	u●*U*●acug^{i}**C**uuuggc*A*a*G*auccu●g●g	68.0 ± 0.0	-6.3	1.2
D20	*Ttr*	P6	g●a●ccag*G*a*UCU*ugccaaagcaL	G20	u●*G*●cuuuggcaaga*U*c*C*ugguc●c●u	80.0 ± 0.7	-	-
D21	*Ttr*	P6	g●a●ccag*G*a*UCU*ugccaaagcaL	G21	u●*G*●cuuu**G**gcaaga*U*c*C*ugguc●c●u	74.0 ± 0.7	-6.0	-
D22	*Ttr*	P6	g●a●ccag*G*a*UCU*ugccaaagcaL	G22	u●*G*●cuuu^{i}**G**gcaaga*U*c*C*ugguc●c●u	77.3 ± 0.4	-2.7	3.3
D23	*Ttr*	P7	g●a●ucuu*G*c*CAA*agcaguagcaL	G23	u●*G*●cuacugcuuug*G*c*A*agauc●c●u	76.8 ± 0.4	-	-
D24	*Ttr*	P7	g●a●ucuu*G*c*CAA*agcaguagcaL	G24	u●*G*●cua**C**ugcuuug*G*c*A*agauc●c●u	68.8 ± 0.4	-8.0	-
D25	*Ttr*	P7	g●a●ucuu*G*c*CAA*agcaguagcaL	G25	u●*G*●cua^{i}**C**ugcuuug*G*c*A*agauc●c●u	70.3 ± 0.4	-6.5	1.5
D26	*F12*	P8	g●g●agcc*C*a*AGA*aagugaaagaL	G26	u●*C*●uuucacuuucu*U*g*G*gcucc●a●c	77.0 ± 0.7	-	-
D27	*F12*	P8	g●g●agcc*C*a*AGA*aagugaaagaL	G27	u●*C*●uuu**C**acuuucu*U*g*G*gcucc●a●c	69.8 ± 0.4	-7.2	-
D28	*F12*	P8	g●g●agcc*C*a*AGA*aagugaaagaL	G28	u●*C*●uuu^{i}**C**acuuucu*U*g*G*gcucc●a●c	71.3 ± 0.4	-5.7	1.5

Italicized uppercase, lower-case, and uppercase bold underlined represent 2'-deoxy-2'-F, 2'-OMe, and (*S*)-GNA modifications, respectively to Adenosine, Cytosine, Guanosine, and Uridine. "L" represents the tri-N-acetylgalactosamine ligand. Phosphorothioate linkages are indicated by the "●" symbol.

RT-qPCR primers and probes

Table S4. Guide Strand RT-qPCR Primers and Probes

siRNAs	Primer ID	Primer Sequence (5' to 3')
**D1, D2, D3**	RT	GTCGTATCCAGTGCAGGGTCCGAGGTATTCGCACTGGATACGACCAGAATGTGA
	Forward qPCR	GCCGCGCTTGTCGATGACTT
	TaqMan Probe	CTGGATACGACCAGAATGTG
	Universal Reverse qPCR	GTGCAGGGTCCGAGGT
**D4, D5, D6**	RT	GTCGTATCCAGTGCAGGGTCCGAGGTATTCGCACTGGATACGACAAAACAGTGT
	Forward qPCR	GCCGCGCTTATAGAGCAAG
	TaqMan Probe	CTGGATACGACAAAACAGT
	Universal Reverse qPCR	GTGCAGGGTCCGAGGT
**D7, D8, D9**	RT	GTCGTATCCAGTGCAGGGTCCGAGGTATTCGCACTGGATACGACAGTGCTTTGA
	Forward qPCR	GCCGCGCTAGAAGCTGAGGC
	TaqMan Probe	CTGGATACGACAGTGCTTT
	Universal Reverse qPCR	GTGCAGGGTCCGAGGT
**D10, D11, D12**	RT	GTCGTATCCAGTGCAGGGTCCGAGGTATTCGCACTGGATACGACTGTTCTTGCT
	Forward qPCR	GCCGCGCACACGGTTTATAG
	TaqMan Probe	CTGGATACGACTGTTCTTG
	Universal Reverse qPCR	GTGCAGGGTCCGAGGT

Off-target silencing in dual-luciferase reporter and analysis

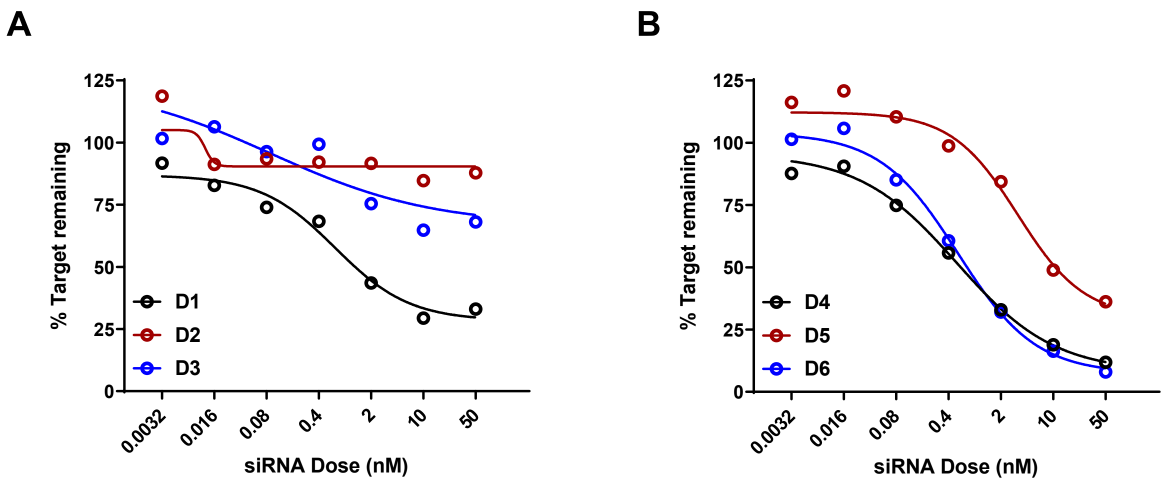

Figure S1. Off-target silencing of parent and GNA-modified GalNAc-siRNAs **D1-D3** (A) or **D4-D6** (B) in the dual-luciferase off-target reporter.

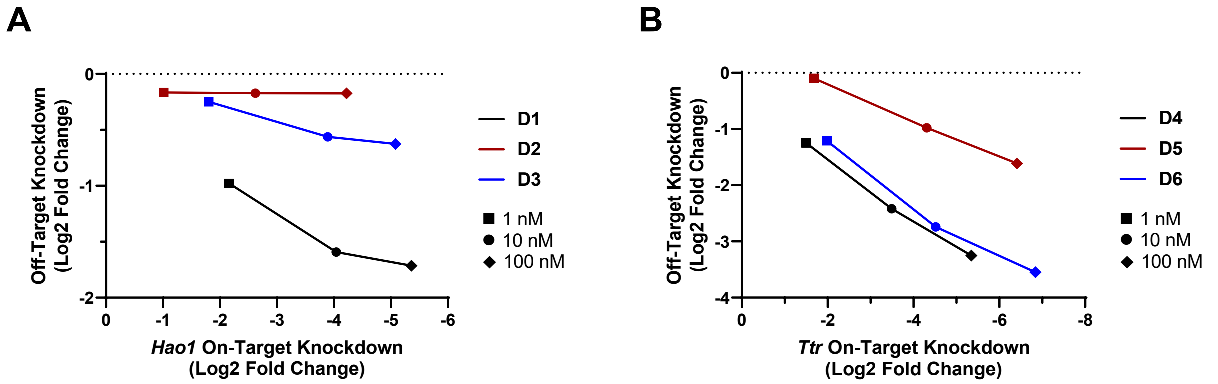

Figure S2. Correlation between off- and on-target activity across doses of **D1-D3** (A) or **D4-D6** (B). On-target activity is the $\log_2$ transformed knockdown after free uptake in PMH (see Figure 6). Off-target activity is the $\log_2$ transformed knockdown in the dual-luciferase reporter assay was inferred from the fitted curves in Figure S1.

<u>**Additional plots and analyses from *in vitro* RNAseq**</u>

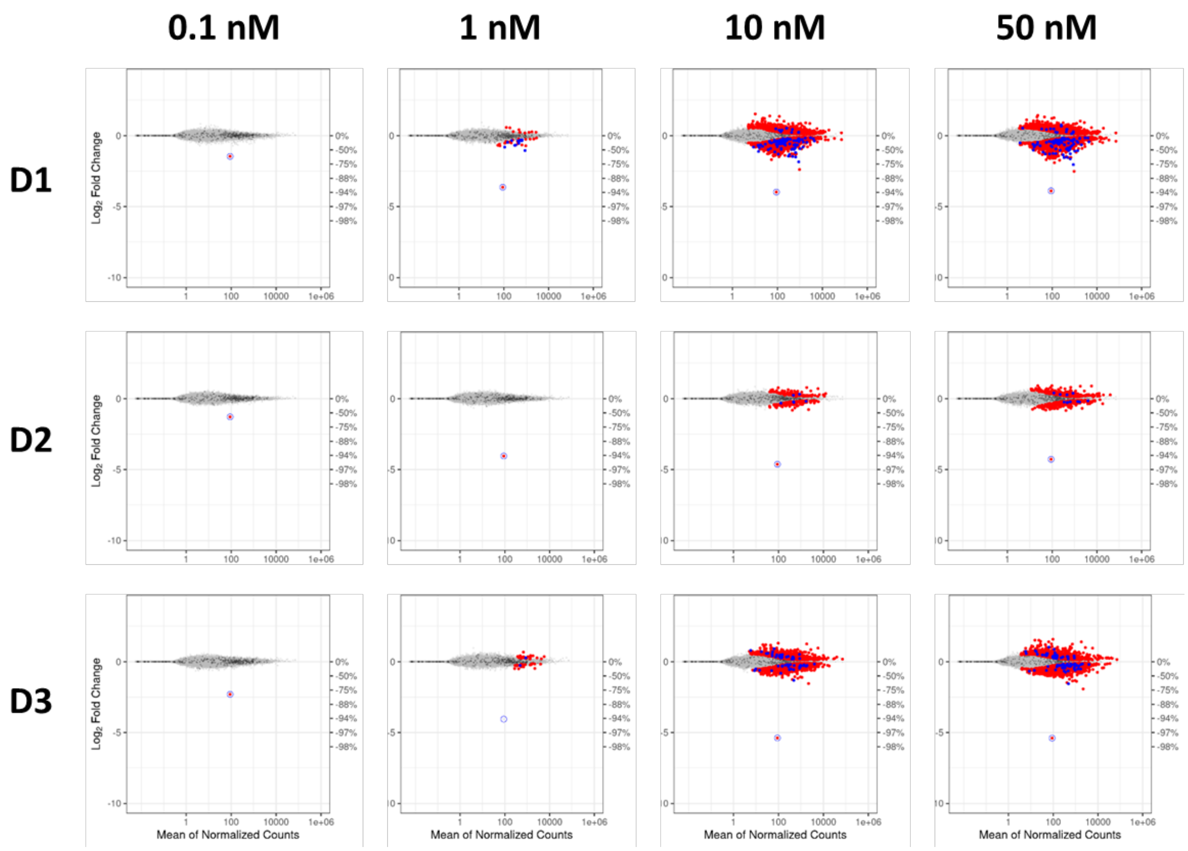

Figure S3. Log$_2$ fold change (MA plot) showing transcriptional dysregulation in primary mouse hepatocytes after transfection of *Hao1*-targeting siRNAs **D1**-**D3**. Dots represent individual mouse transcripts, their average read count, and the level of change in expression compared to the mock control. Whereas grey dots represent genes which were not determined to be differentially expressed after siRNA treatment relative to the control, the blue and red dots represent differentially expressed genes (false discovery rate <0.05) with or without a canonical miRNA match (8mer, 7mer-A1, 7mer-m8) to the guide seed region, respectively. On-target knockdown is represented by the circled dot.

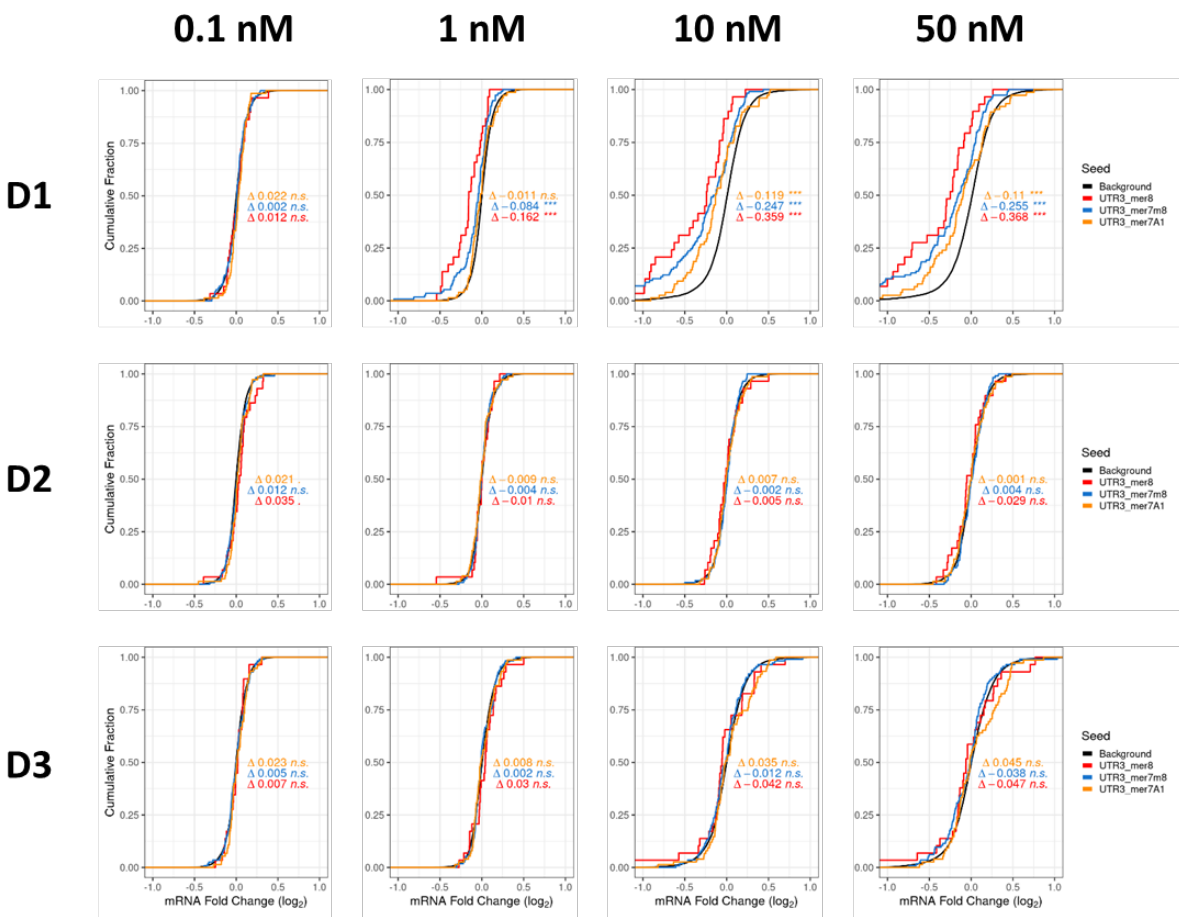

Figure S4. Cumulative distribution function (CDF) plots relative to control after treatment with the designated *Hao1*-targeting siRNAs **D1-D3** at the specified dose. Each colored line represents the impact of different types of seed matches in the 3'-UTR on the cumulative distribution of expression change of transcripts; black = background, yellow = mer7-A1, blue = mer7-m8, red = mer8. Delta estimates indicate the magnitude of each CDF shift vs. background.

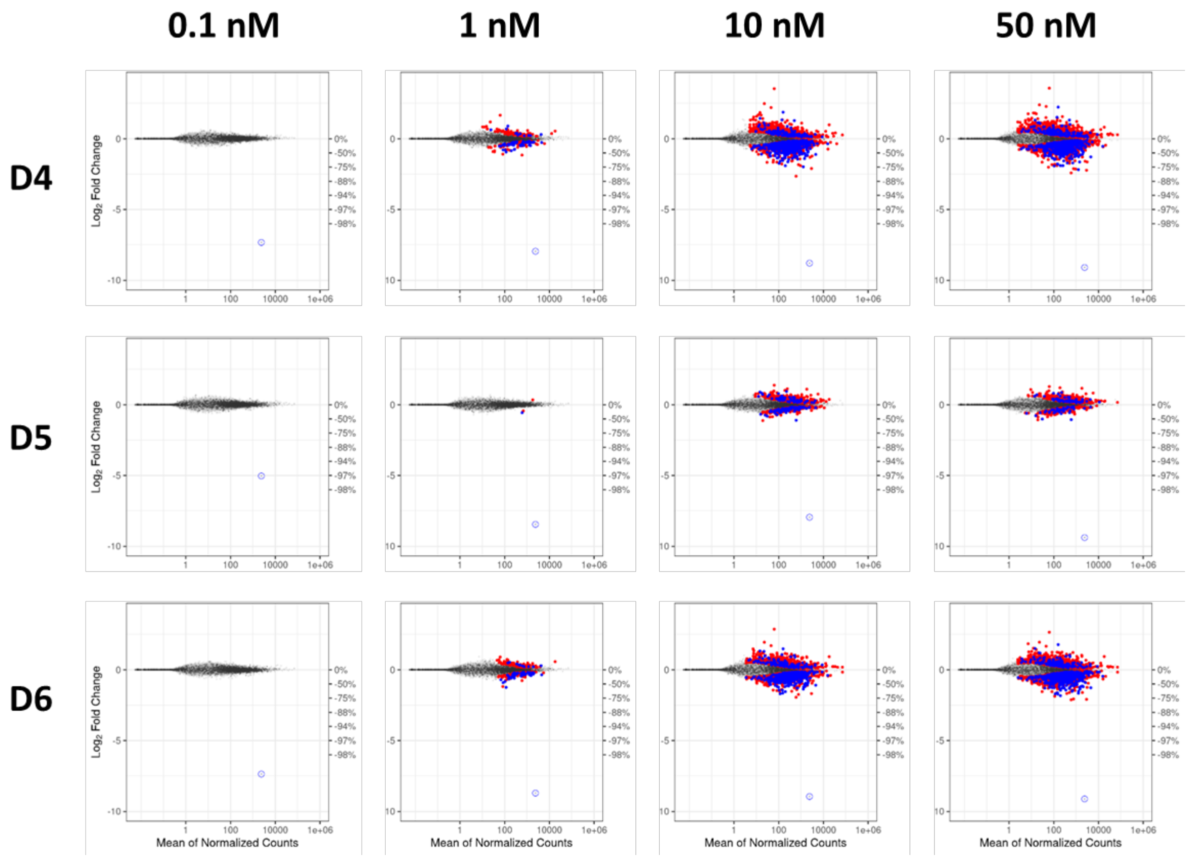

Figure S5. Log$_2$ fold change (MA plot) showing transcriptional dysregulation in primary mouse hepatocytes after transfection of *Ttr*-targeting siRNAs **D4-D6**. Dots represent individual mouse transcripts, their average read count, and the level of change in expression compared to the mock control. Whereas grey dots represent genes which were not determined to be differentially expressed after siRNA treatment relative to the control, the blue and red dots represent differentially expressed genes (false discovery rate <0.05) with or without a canonical miRNA match (8mer, 7mer-A1, 7mer-m8) to the guide seed region, respectively. On-target knockdown is represented by the circled dot.

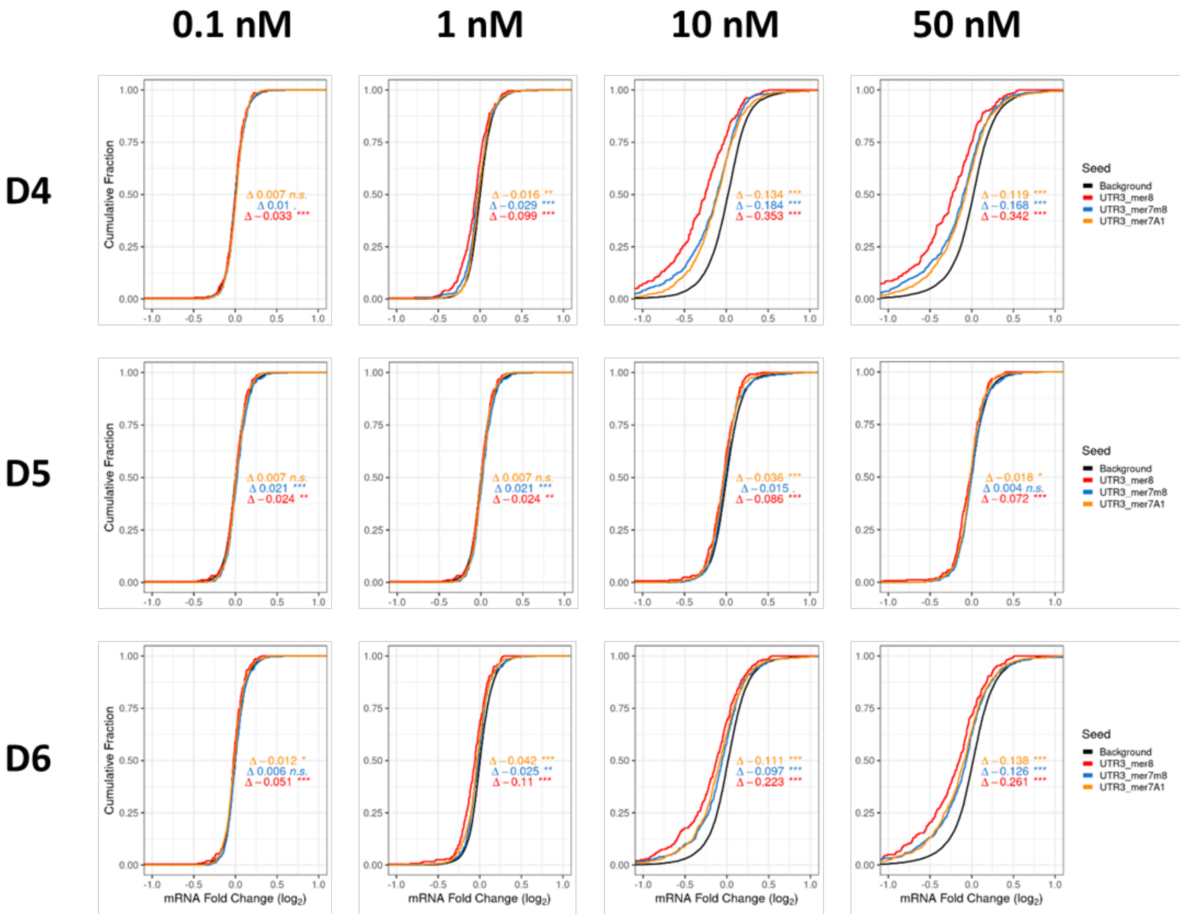

Figure S6. Cumulative distribution function (CDF) plots relative to control after treatment with the designated *Ttr*-targeting siRNAs **D4-D6** at the specified dose. Each colored line represents the impact of different types of seed matches in the 3'-UTR on the cumulative distribution of expression change of transcripts; black = background, yellow = mer7-A1, blue = mer7-m8, red = mer8. Delta estimates indicate the magnitude of each CDF shift vs. background.

Table S5. Summary of *in vitro* RNA sequencing data

siRNA	Dose (nM)	On-Target[a] (%KD)	*CDF Shift* mer8	mer7m8	mer7A1	*DEGs* Total Down	Total Up	Total DEGs
**D1**	0.1	-1.46 (64)	0.012	0.002	0.022	0	1	1
	1	-3.64 (92)	-0.162	-0.084	-0.011	10	37	47
	10	-3.98 (94)	-0.359	-0.247	-0.119	1152	1321	2473
	50	-3.89 (93)	-0.368	-0.255	-0.110	1345	1601	2946
**D2**	0.1	-1.29 (59)	0.035	0.012	0.021	0	1	1
	1	-4.05 (94)	-0.010	-0.004	-0.009	0	1	1
	10	-4.63 (96)	-0.005	-0.002	0.007	110	118	228
	50	-4.28 (95)	-0.029	0.004	-0.001	342	368	710
**D3**	0.1	-2.30 (80)	0.007	0.005	0.023	0	1	1
	1	-4.05 (94)	0.030	0.002	0.008	22	24	46
	10	-5.38 (98)	-0.042	-0.012	0.035	1000	1104	2104
	50	-5.39 (98)	-0.047	-0.038	0.045	1321	1668	2989
**D4**	0.1	-7.32 (99)	-0.033	0.010	0.007	0	0	0
	1	-7.95 (100)	-0.099	-0.029	-0.016	98	115	213
	10	-8.79 (100)	-0.353	-0.184	-0.134	1600	1854	3454
	50	-9.09 (100)	-0.342	-0.168	-0.119	1809	2006	3815
**D5**	0.1	-5.03 (97)	-0.024	0.021	0.007	0	0	0
	1	-8.45 (100)	-0.050	-0.002	-0.009	1	2	3
	10	-7.95 (10)	-0.086	-0.015	-0.036	549	359	908
	50	-9.38 (100)	-0.072	0.004	-0.018	550	259	809
**D6**	0.1	-7.36 (99)	-0.051	0.006	-0.012	0	0	0
	1	-8.71 (100)	-0.110	-0.025	-0.042	107	130	237
	10	-8.95 (100)	-0.223	-0.097	-0.111	1383	1509	2892
	50	-9.12 (100)	-0.261	-0.126	-0.138	1645	1899	3544

[a]Log_2 fold change relative to mock control.

Mass Identification of siRNA Metabolites in Mouse Liver

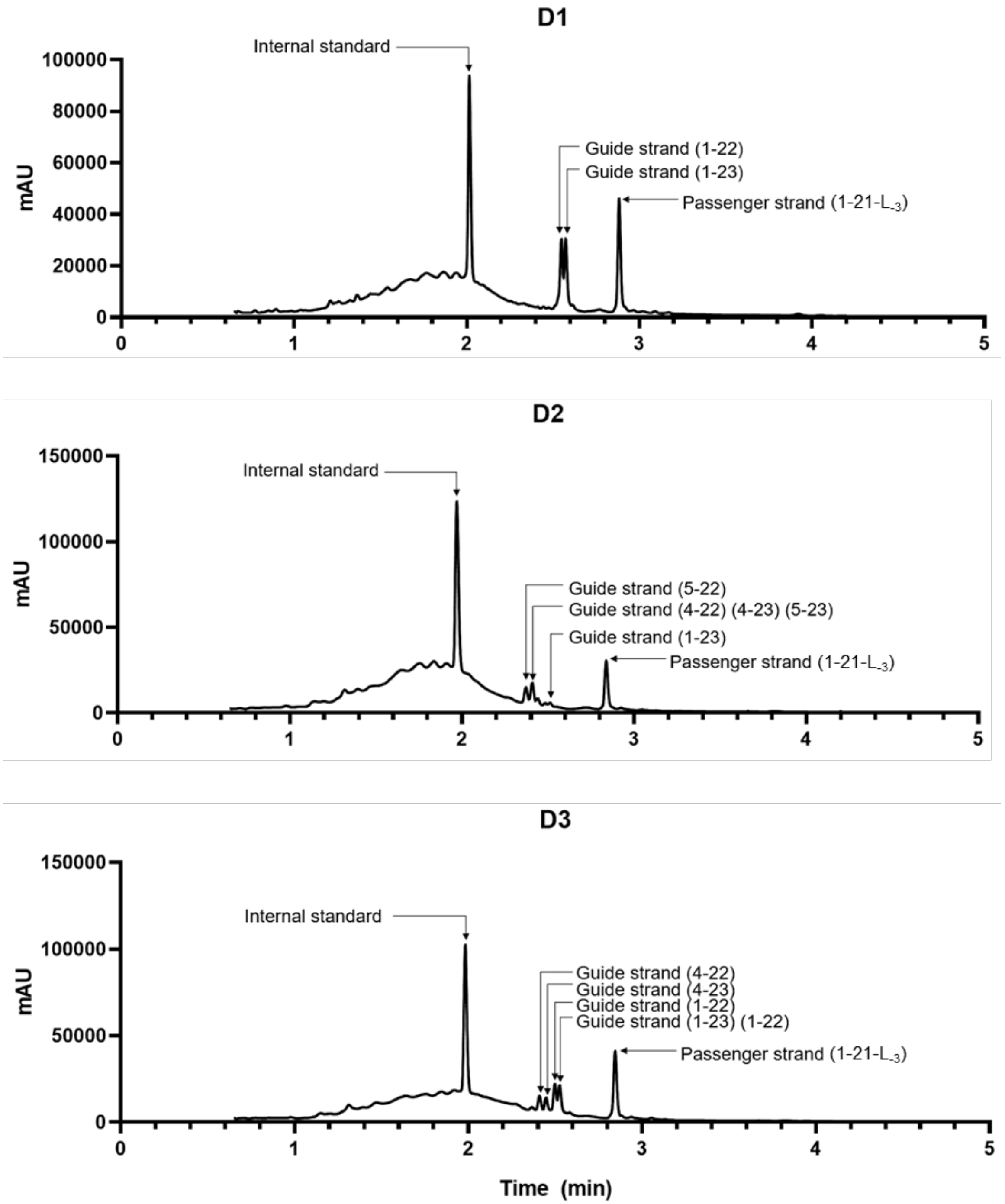

Figure S7. Representative UV Chromatograms for *in vivo* mouse MetID with arrows pointing to signal corresponding to observed fragments. See Table S6 and Experimental Section for further details.

Table S6. Metabolic Identification from Mouse Liver

siRNA	Observed MW	Identified Fragment (5' to 3')	Strand	Nucleotides in Fragment (5' to 3')	% of Total Strand
D1	7932.729	g●a●augu*G*aa*AG*ucaucgacaaL$_{-3}$	Passenger	1-21-L$_{-3}$	89
	7054.210	g●a●augu*G*aa*AG*ucaucgacaa	Passenger	1-21	1
	6368.078	g●a●augu*G*aa*AG*ucaucgac	Passenger	1-19	4
	5705.948	g●a●augu*G*aa*AG*ucaucg	Passenger	1-17	1
	5346.883	g●a●augu*G*aa*AG*ucauc	Passenger	1-16	1
	7557.683	a●augu*G*aa*AG*ucaucgacaaL$_{-3}$	Passenger	2-21-L$_{-3}$	4
	7534.058	u●*U*●guc*G*a*UG*acuu*U*c*A*cauuc●u●g	Guide	1-23	54
	7159.016	u●*U*●guc*G*a*UG*acuu*U*c*A*cauuc●u	Guide	1-22	43
	4530.709	acuu*U*c*A*cauuc●u●g	Guide	10-23	1
	4187.639	cuu*U*c*A*cauuc●u●g	Guide	11-23	1
	5819.896	c*G*a*UG*acuu*U*c*A*cauuc●u	Guide	5-22	1
	3812.589	cuu*U*c*A*cauuc●u	Guide	11-22	1
D2	8032.799	g●a●augu*G*aa*AG*ucaucgacaaL$_{-2}$	Passenger	1-21-L$_{-2}$	1
	7932.735	g●a●augu*G*aa*AG*ucaucgacaaL$_{-3}$	Passenger	1-21-L$_{-3}$	96
	5346.875	g●a●augu*G*aa*AG*ucauc	Passenger	1-16	3
	7462.048	u●*U*●gu**C** *G*a*UG*acuu*U*c*A*cauuc●u●g	Guide	1-23	9
	7087.007	u●*U*●gu**C** *G*a*UG*acuu*U*c*A*cauuc●u	Guide	1-22	4
	6442.961	u**C** *G*a*UG*acuu*U*c*A*cauuc●u●g	Guide	4-23	16
	6122.913	**C** *G*a*UG*acuu*U*c*A*cauuc●u●g	Guide	5-23	31
	4530.708	acuu*U*c*A*cauuc●u●g	Guide	10-23	1
	6067.919	u**C** *G*a*UG*acuu*U*c*A*cauuc●u	Guide	4-22	14
	5747.861	**C** *G*a*UG*acuu*U*c*A*cauuc●u	Guide	5-22	26
D3	8032.779	g●a●augu*G*aa*AG*ucaucgacaaL$_{-2}$	Passenger	1-21-L$_{-2}$	1
	7932.727	g●a●augu*G*aa*AG*ucaucgacaaL$_{-3}$	Passenger	1-21-L$_{-3}$	95
	7054.202	g●a●augu*G*aa*AG*ucaucgacaa	Passenger	1-21	1
	5705.941	g●a●augu*G*aa*AG*ucaucg	Passenger	1-17	1
	5346.878	g●a●augu*G*aa*AG*ucauc	Passenger	1-16	3
	7462.036	u●*U*●gu^{i}**C** *G*a*UG*acuu*U*c*A*cauuc●u●g	Guide	1-23	25
	7086.995	u●*U*●gu^{i}**C** *G*a*UG*acuu*U*c*A*cauuc●u	Guide	1-22	43
	6442.956	u^{i}**C** *G*a*UG*acuu*U*c*A*cauuc●u●g	Guide	4-23	21
	6122.915	^{i}**C** *G*a*UG*acuu*U*c*A*cauuc●u●g	Guide	5-23	1
	6067.916	u^{i}**C** *G*a*UG*acuu*U*c*A*cauuc●u	Guide	4-22	10
	5747.875	^{i}**C** *G*a*UG*acuu*U*c*A*cauuc●u	Guide	5-22	1

Italicized uppercase, lower-case, and uppercase bold underlined letters represent 2'-deoxy-2'-F, 2'-OMe, and (*S*)-GNA modifications, respectively to adenosine, cytosine, guanosine, and uridine. "L" represents the tri-N-acetylgalactosamine ligand, and "L$_{-2}$" or "L$_{-3}$" represent loss of all three sugars and two or three linkers from "L". Structures shown in Figure S8 below. Phosphorothioate linkages are indicated by the "●" symbol. Percent of strand is calculated based on TIC intensity summed for all metabolites of a given strand.

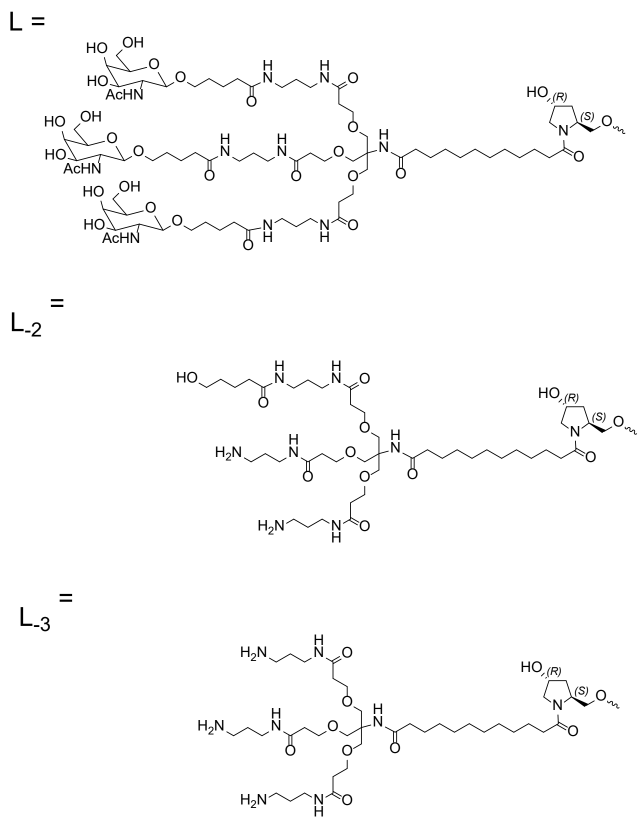

Figure S8. Structure of "L", "L$_{-2}$", and "L$_{-3}$".

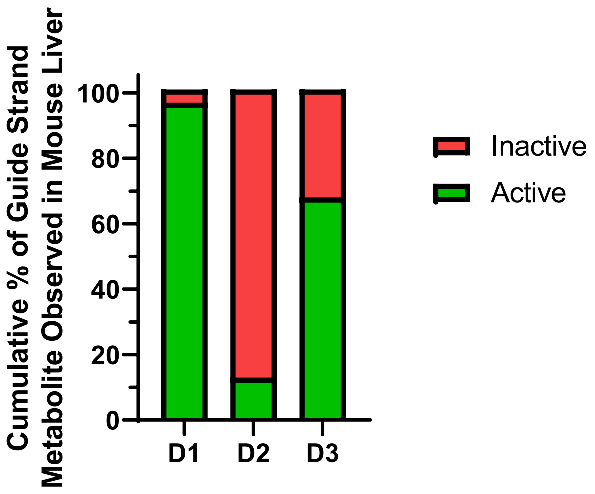

Figure S9. Percent of guide strand metabolites observed in mouse liver relative to all metabolites detected in each sample (see Table S6). Active metabolites are defined as the sum of full-length guide strand (nucleotides 1-23) and the 3'-N-1 metabolite (nucleotides 1-22). All other guides strand metabolites in Table S6 are considered unlikely to be active.

^{1}H NMR of compound **3** (500 MHz, DMSO-d_6)

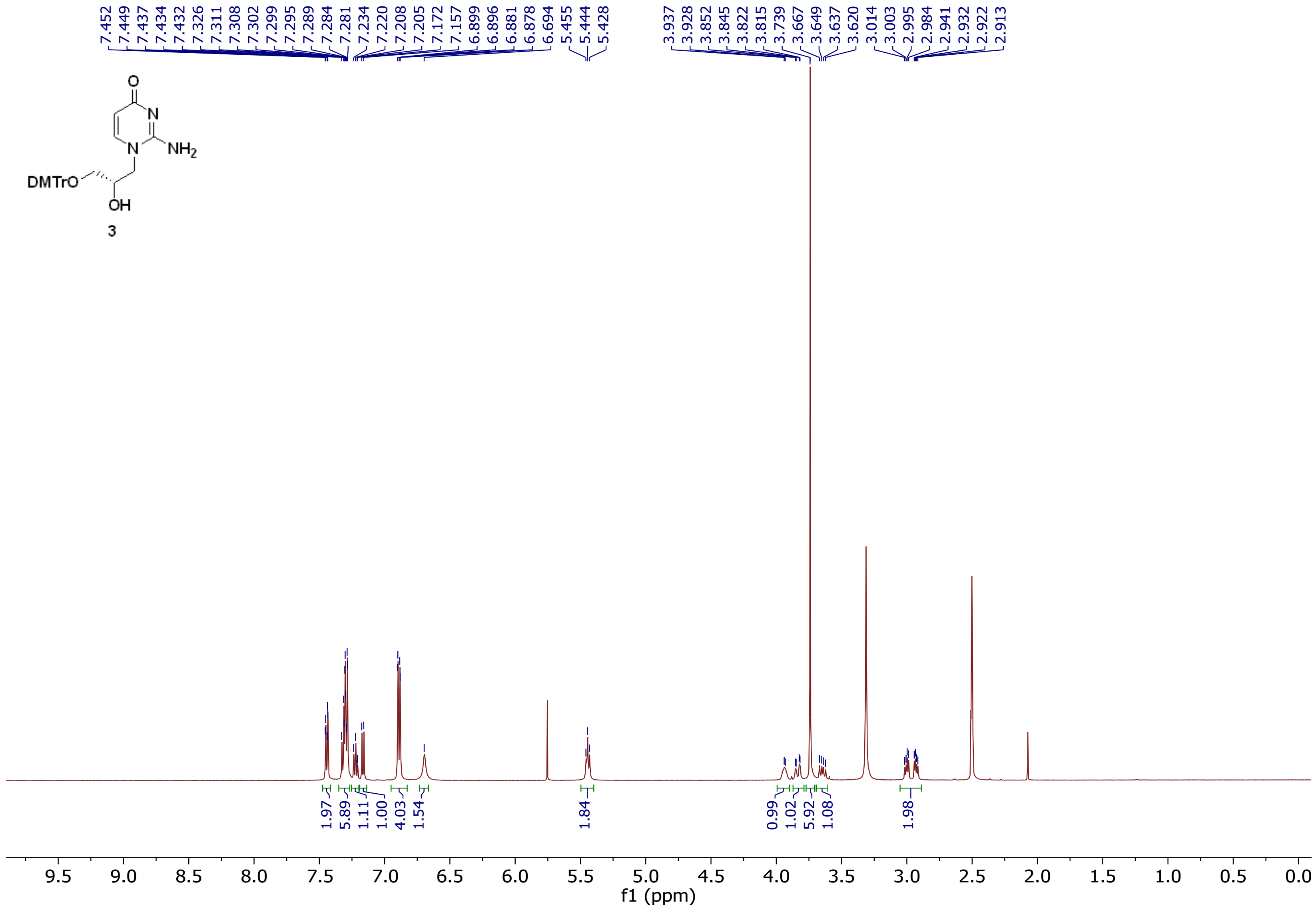

^{13}C NMR of compound **3** (126 MHz, DMSO-d_6)

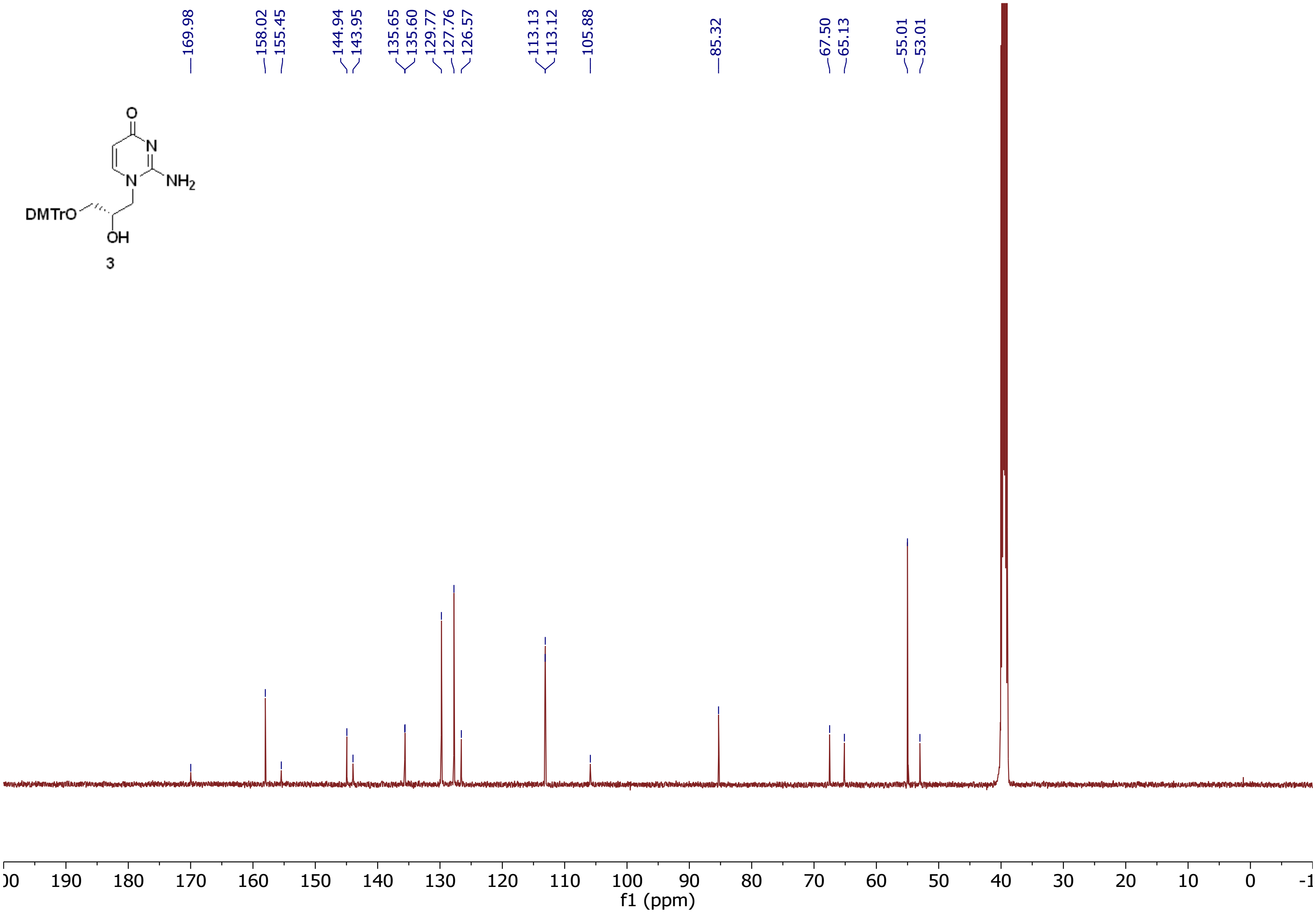

^{1}H NMR of compound **4** (400 MHz, DMSO-d_6)

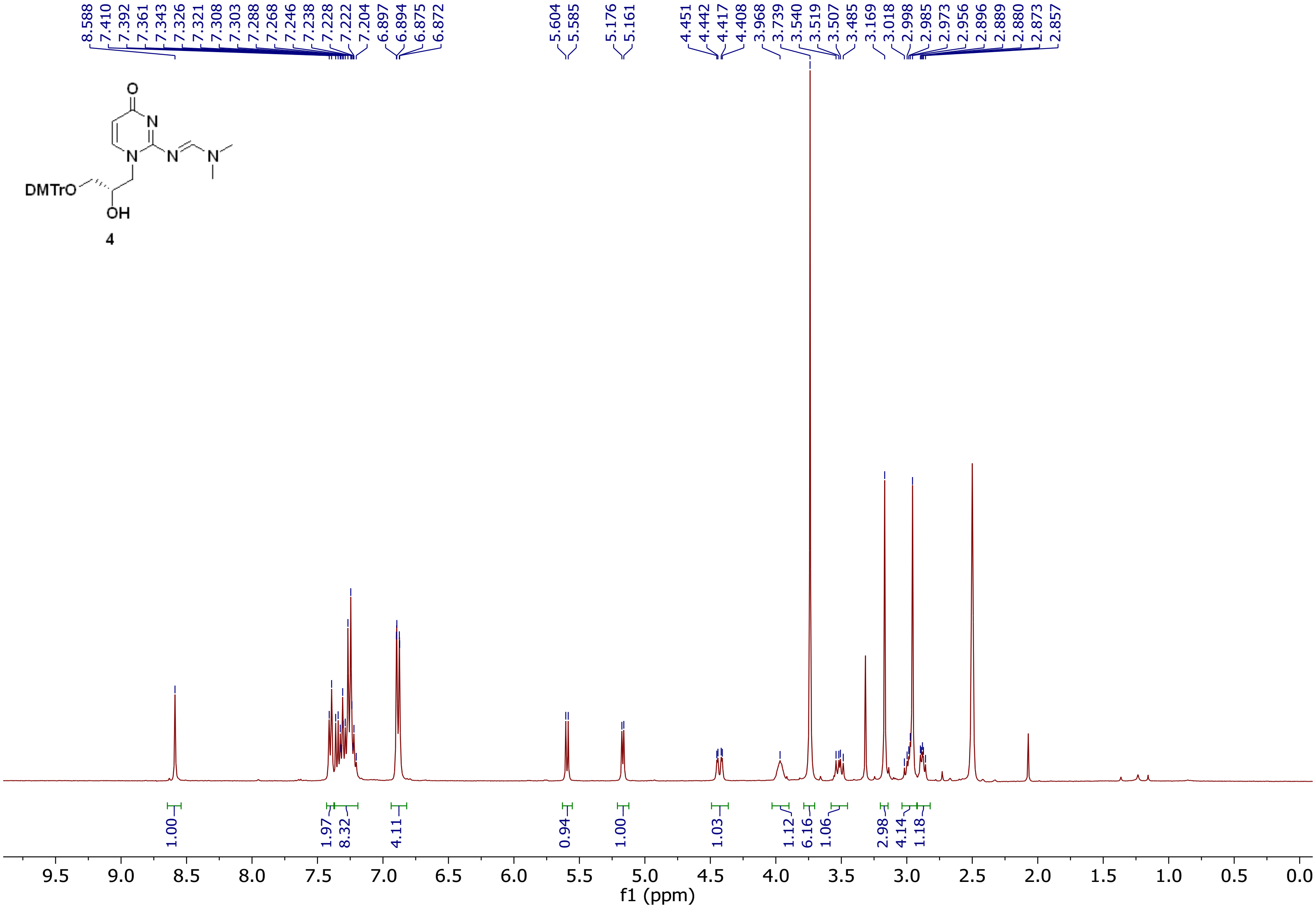

^{13}C NMR of compound **4** (126 MHz, DMSO-d_6)

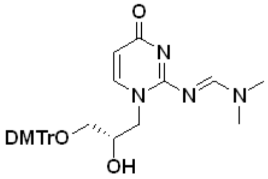

4

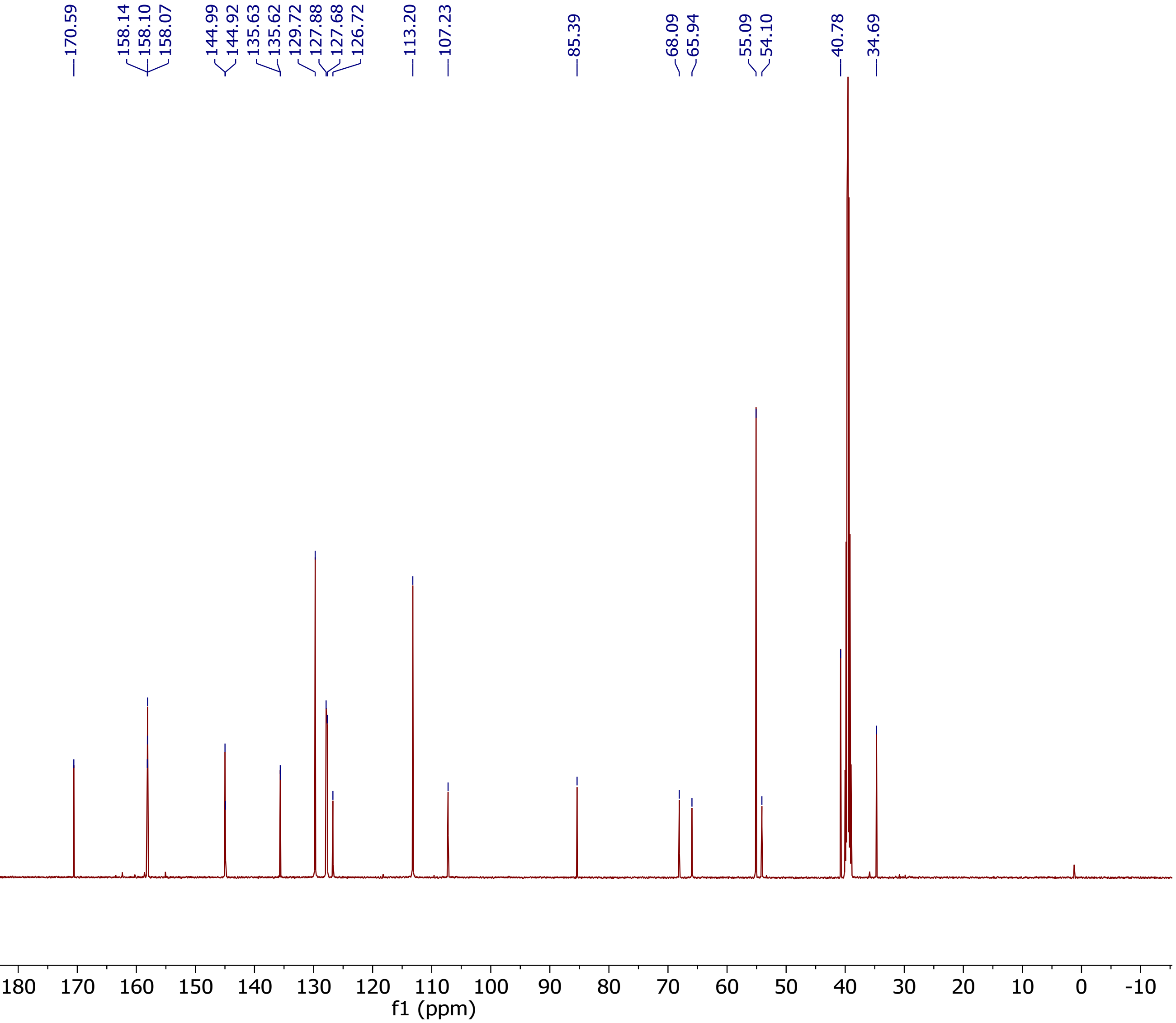

^{1}H NMR of phosphoramidite ***i*C** (500 MHz, CD_3CN)

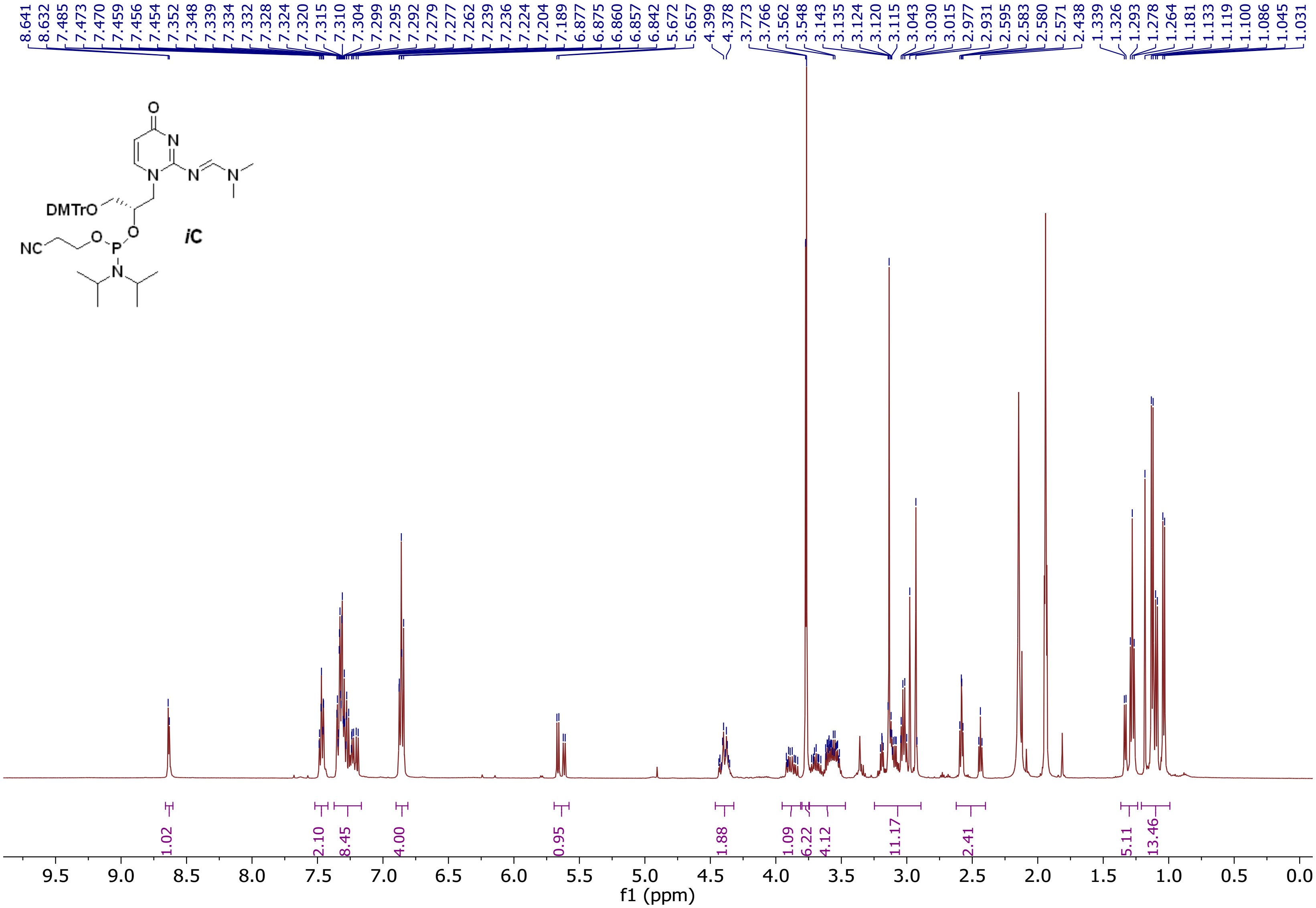

^{13}C NMR of phosphoramidite ***i*C** (126 MHz, CD_3CN)

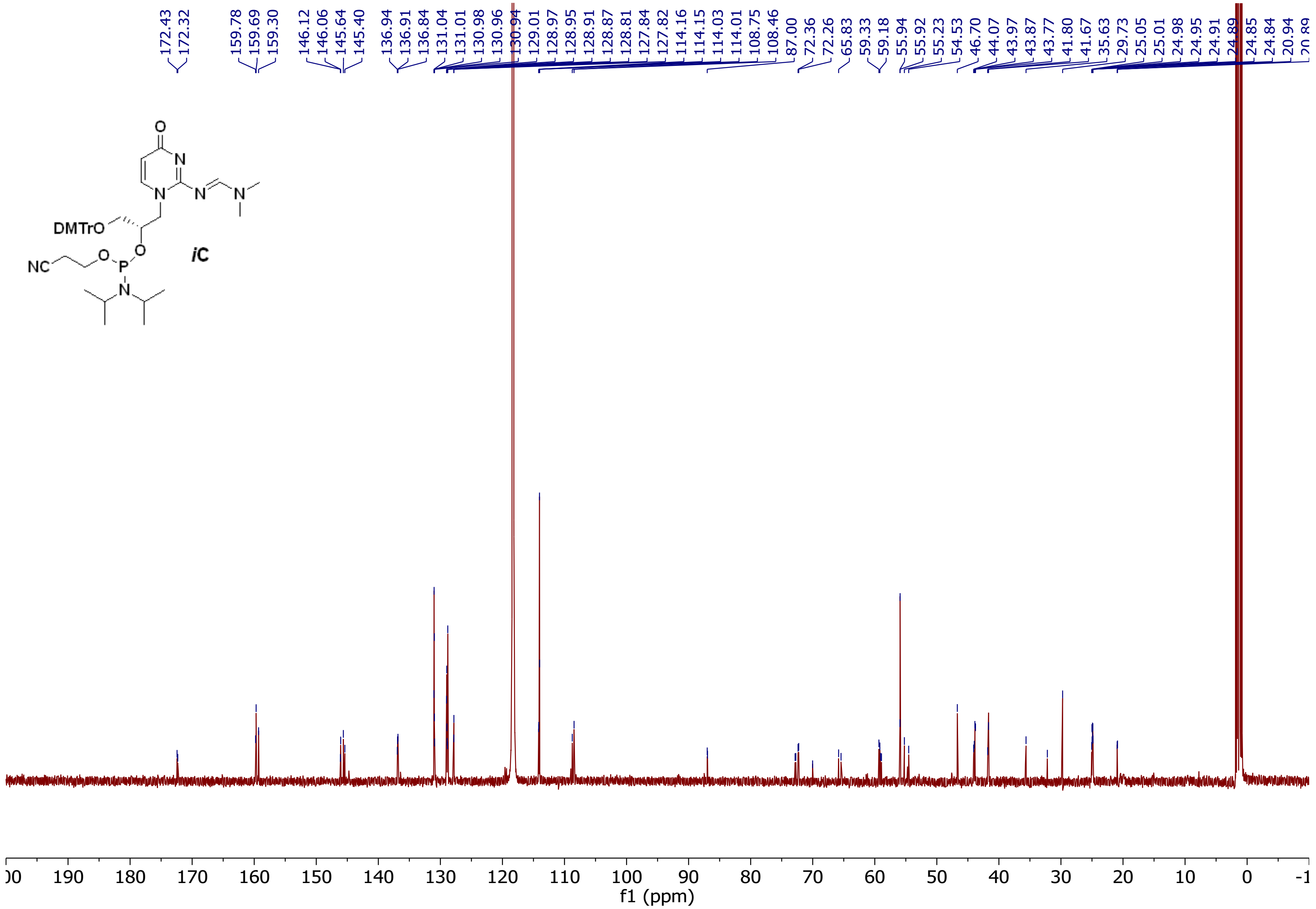

^{31}P NMR of phosphoramidite ***i*C** (202 MHz, CD_3CN)

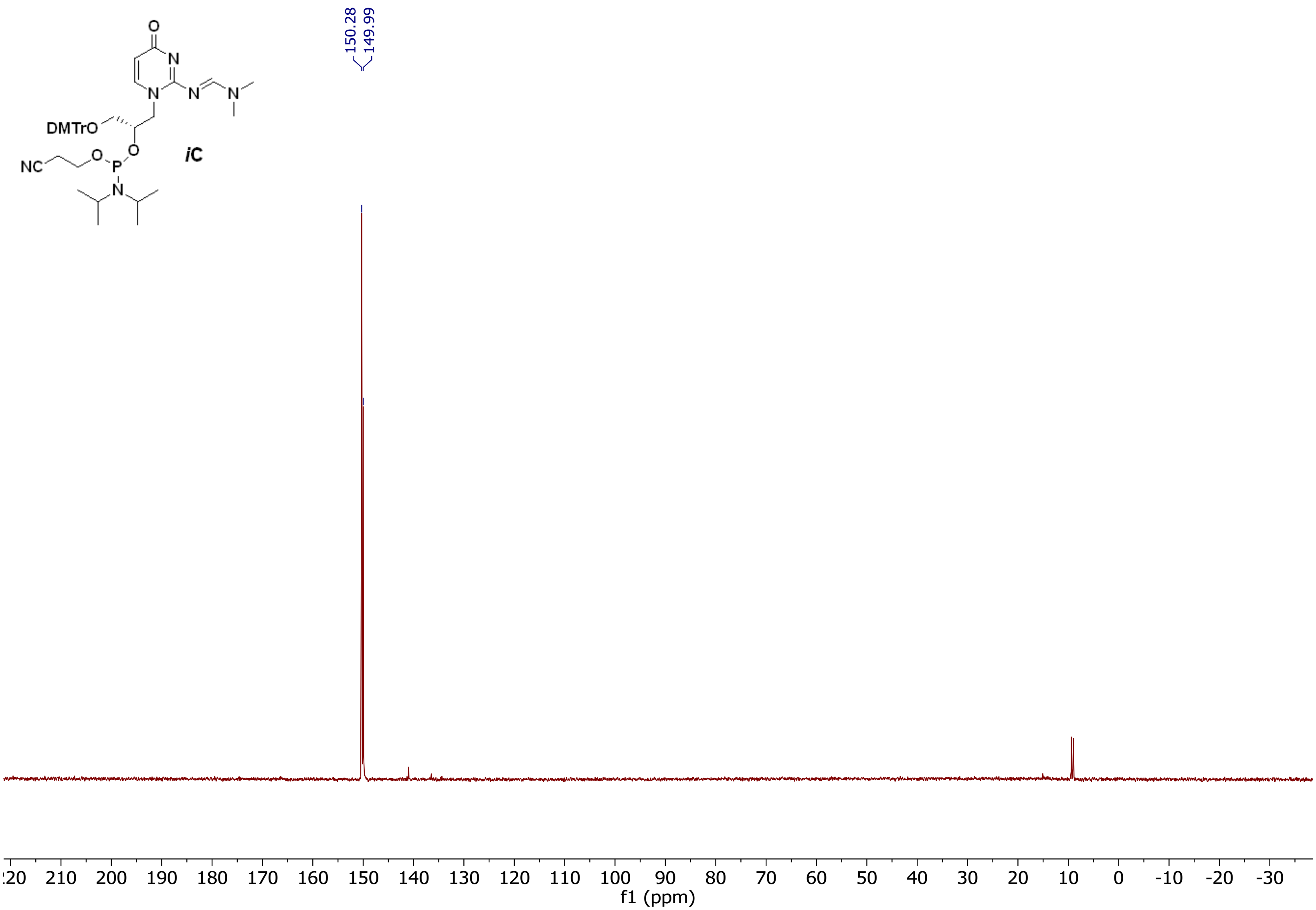

^{1}H NMR spectrum of compound **7** (400 MHz, $CDCl_3$)

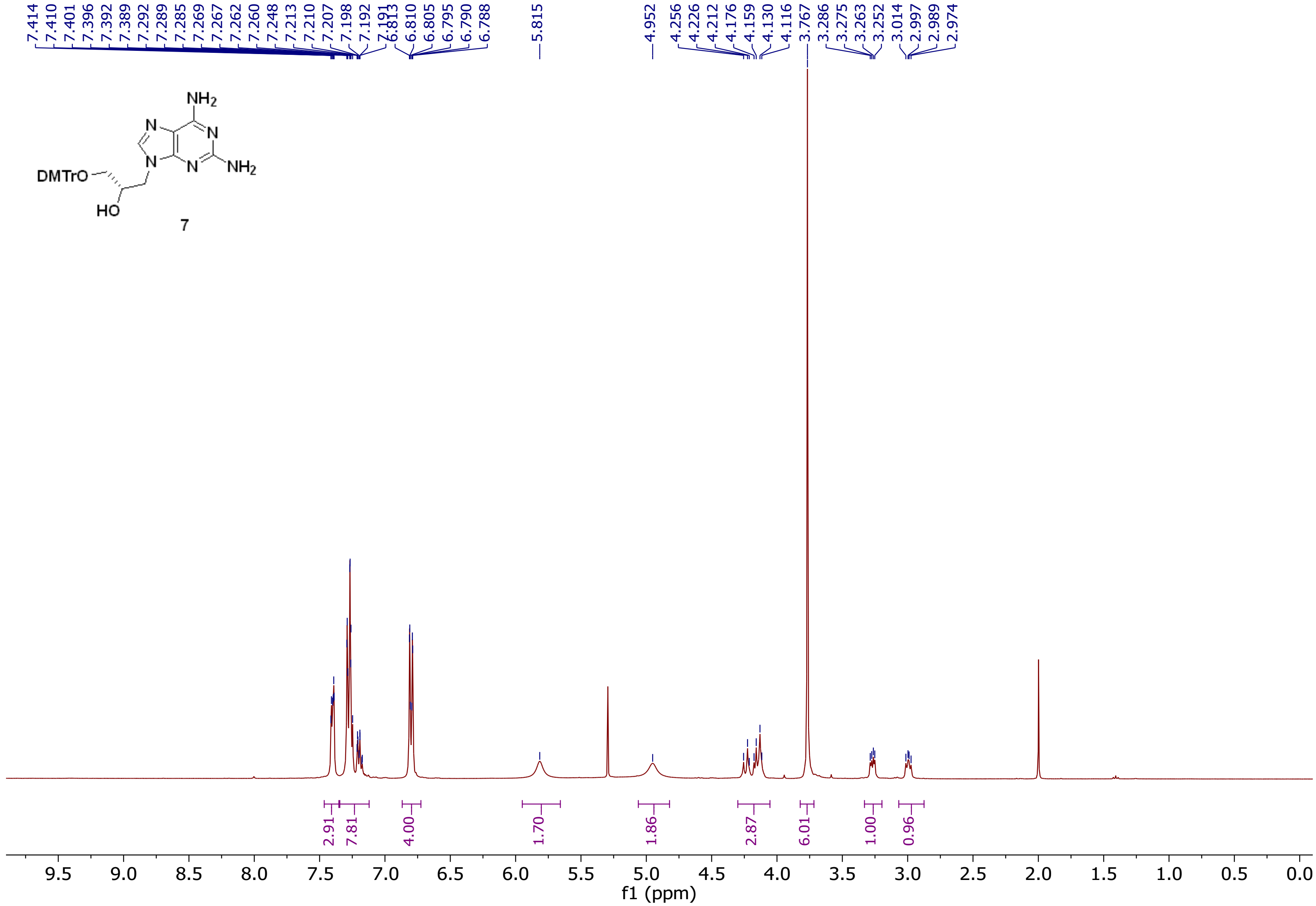

^{13}C NMR spectrum of compound **7** (101 MHz, $CDCl_3$)

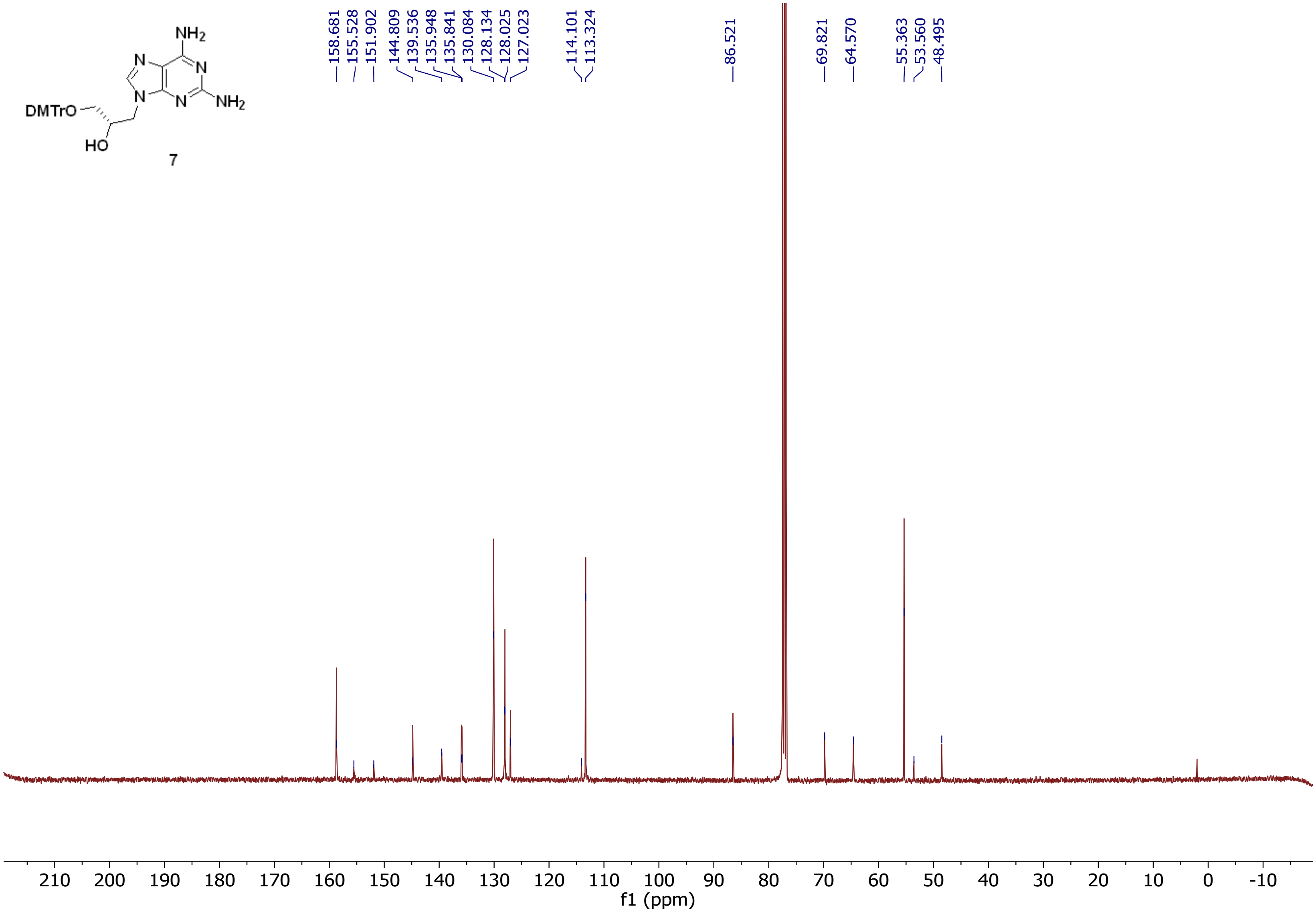

^{1}H NMR spectrum of the acetate salt of compound **8** (400 MHz, D_2O)

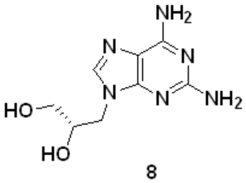

8

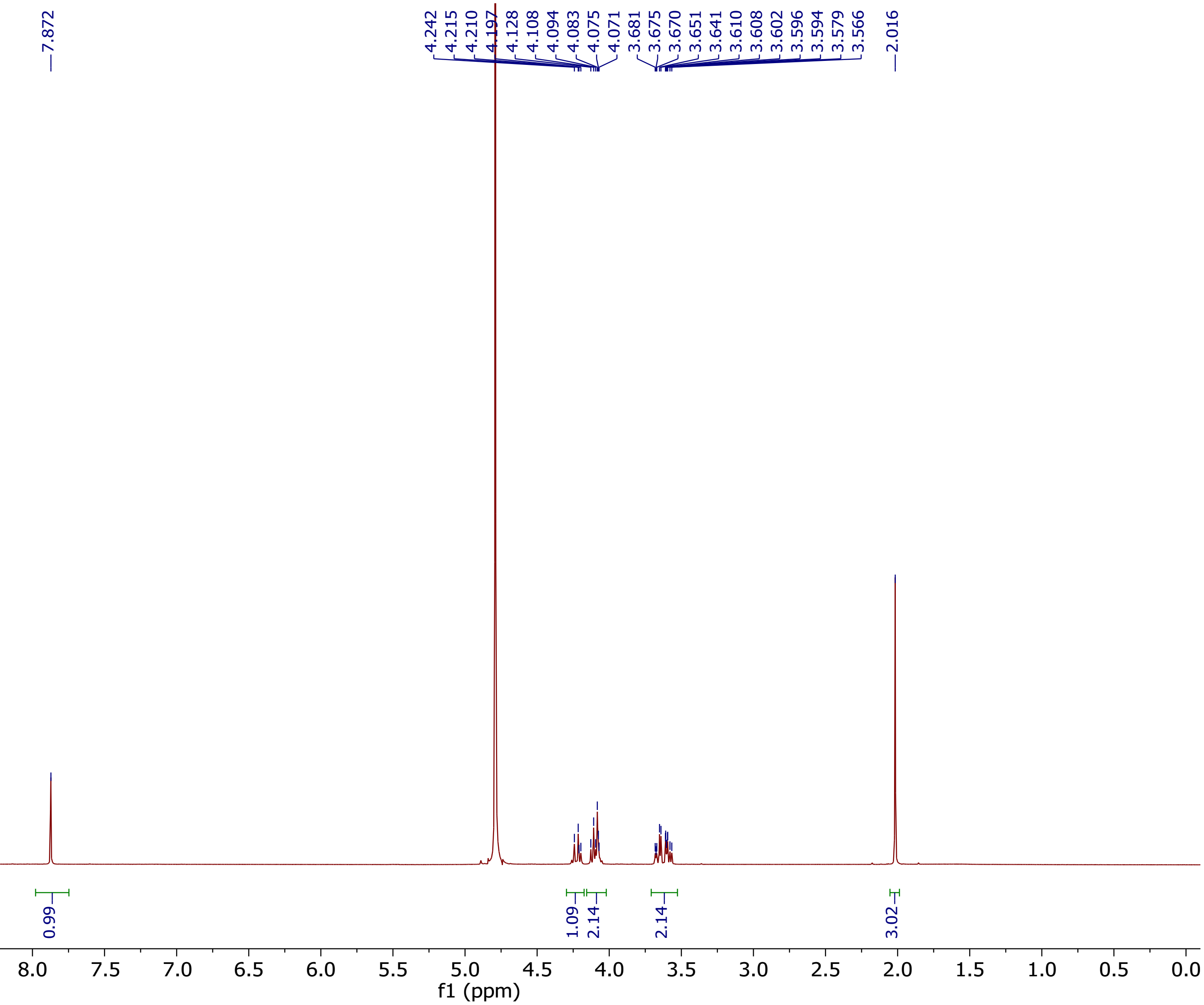

^{13}C NMR spectrum of the acetate salt of compound **8** (126 MHz, D_2O)

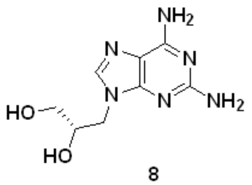

8

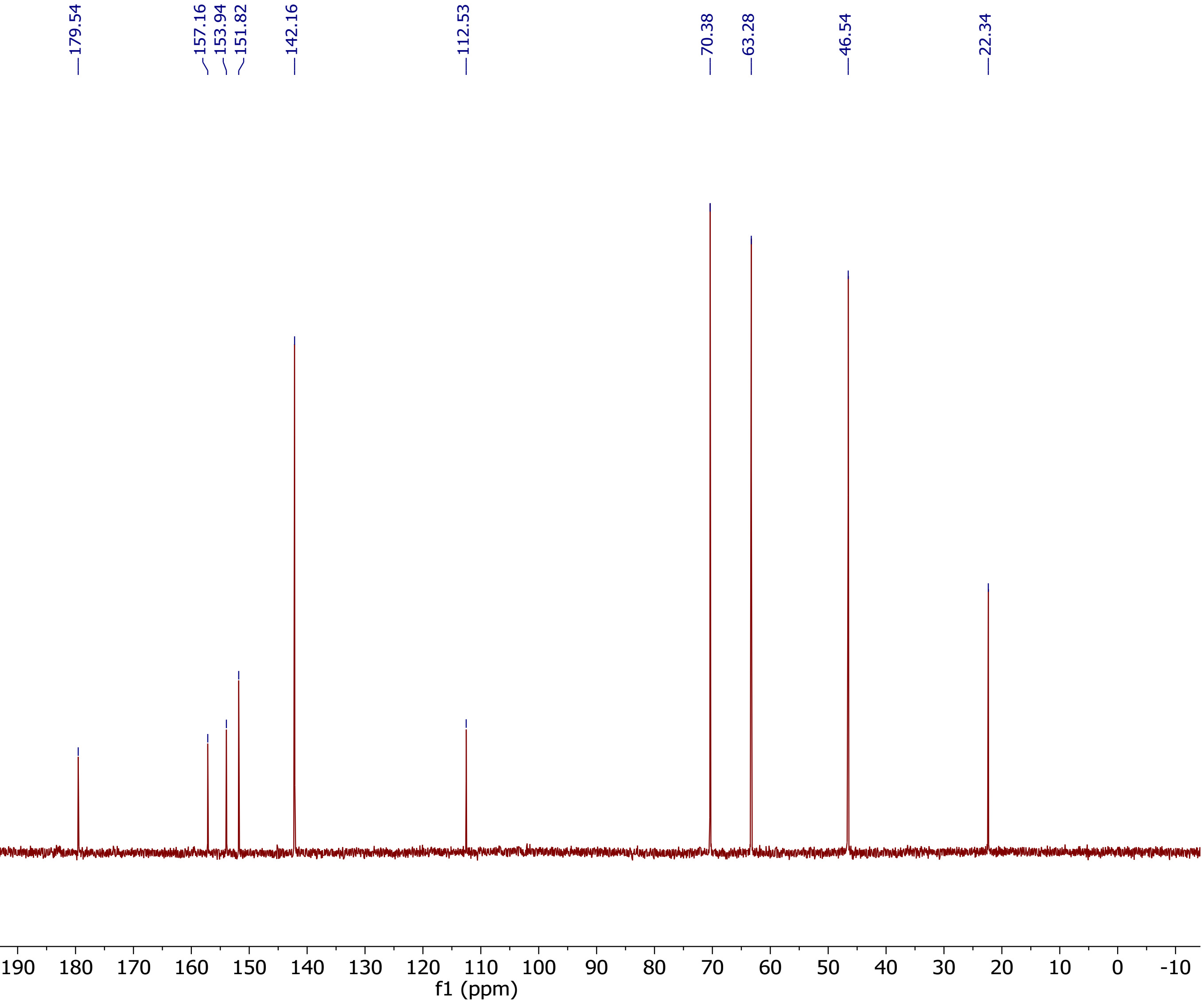

^{1}H NMR spectrum of the acetate salt of compound **9** (500 MHz, DMSO-d_6)

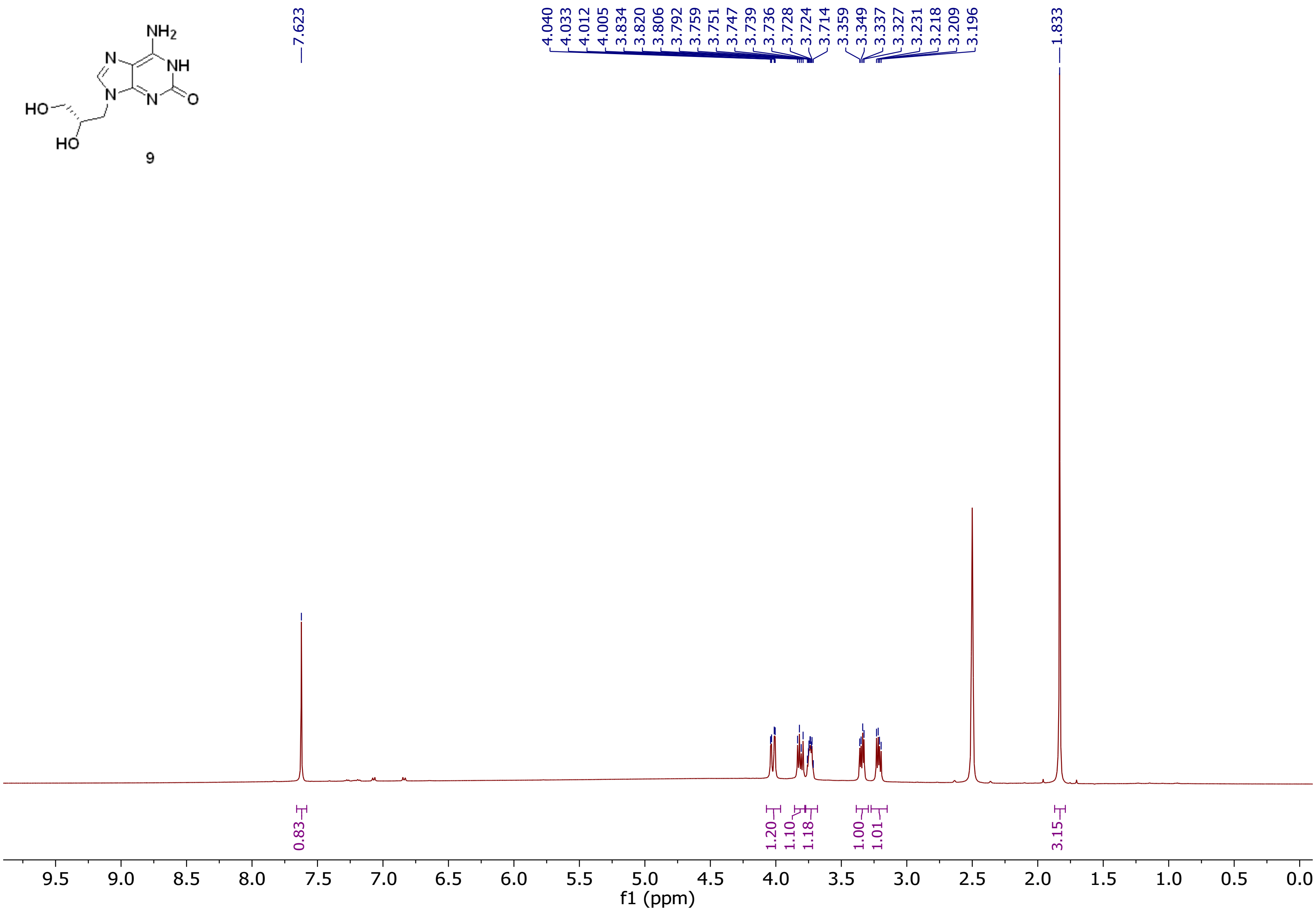

^{13}C NMR spectrum of the acetate salt of compound **9** (126 MHz, DMSO-d_6)

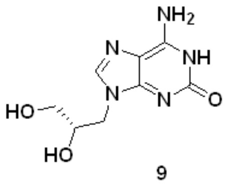

9

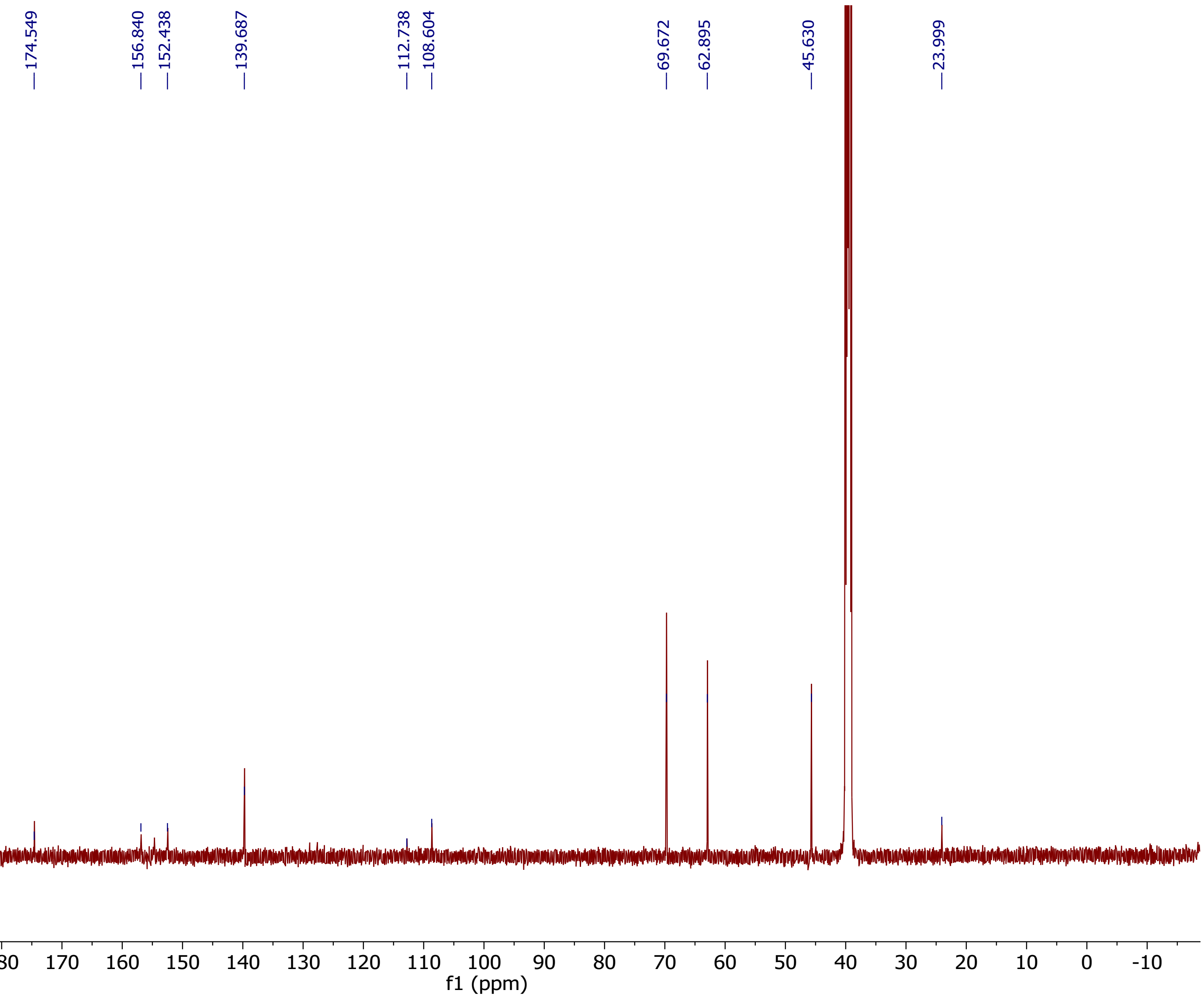

^{1}H NMR spectrum of compound **10** (400 MHz, DMSO-d_6)

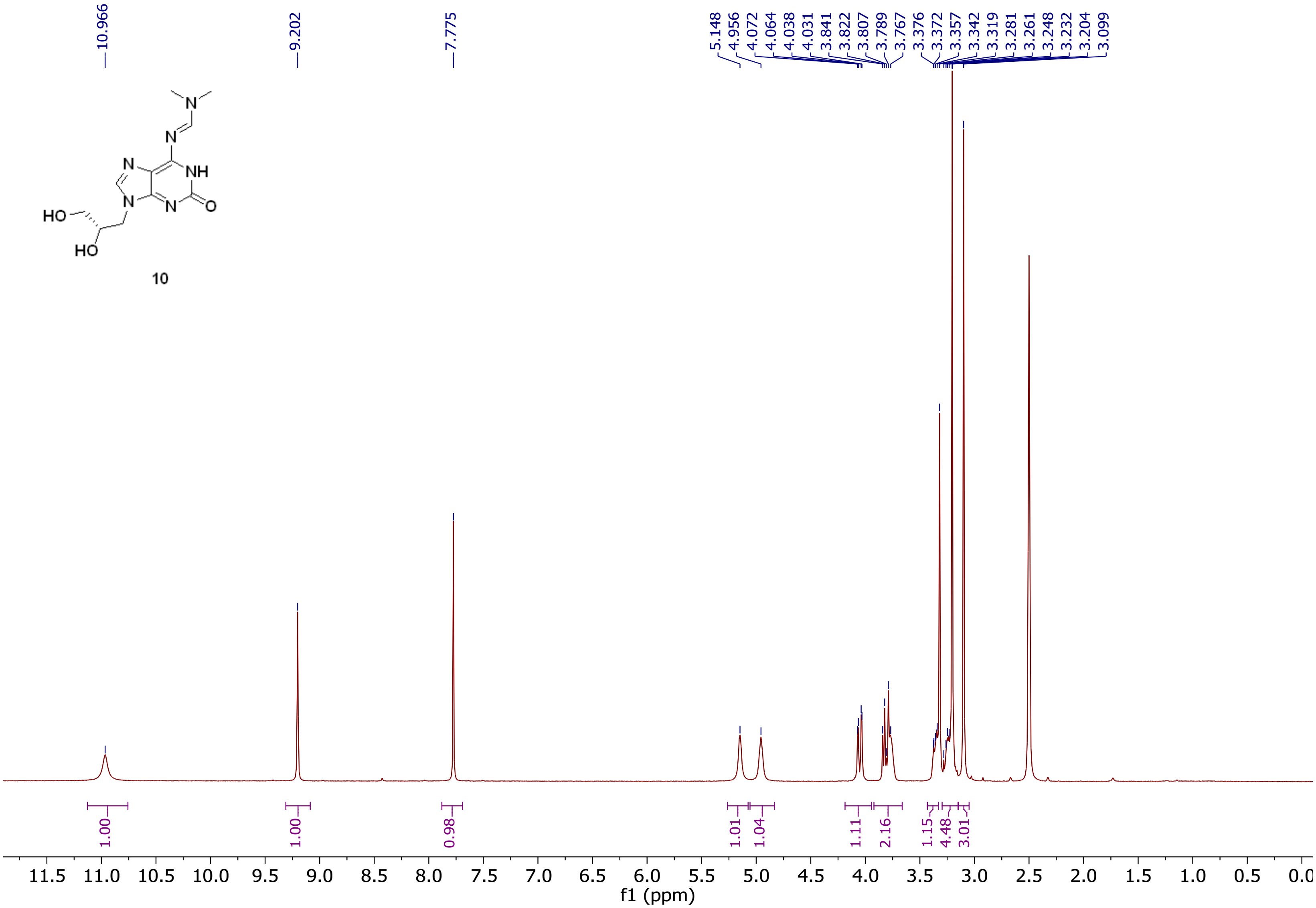

^{13}C NMR spectrum of compound **10** (126 MHz, DMSO-d_6)

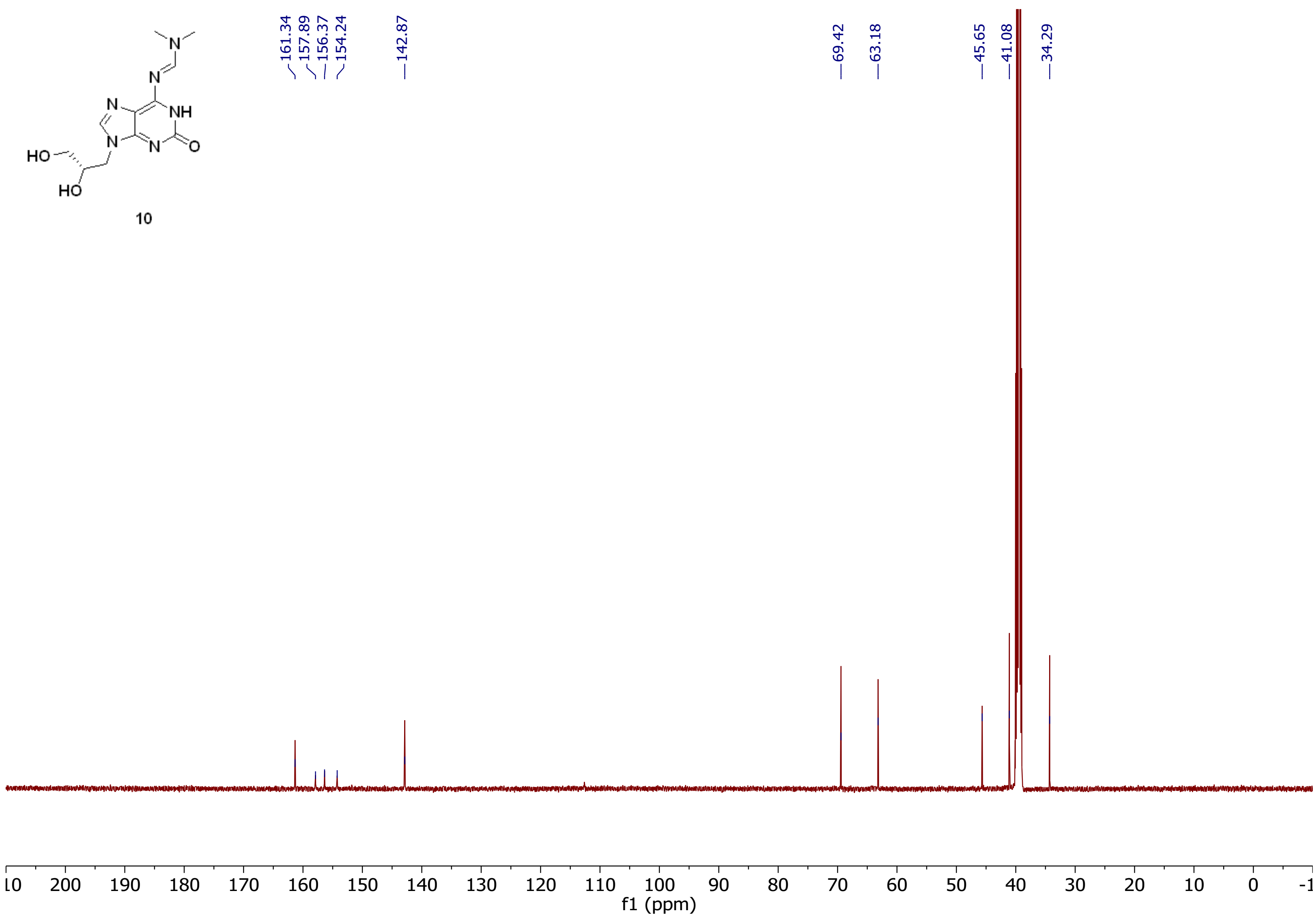

^{1}H NMR spectrum of compound **11** (400 MHz, DMSO-d_6)

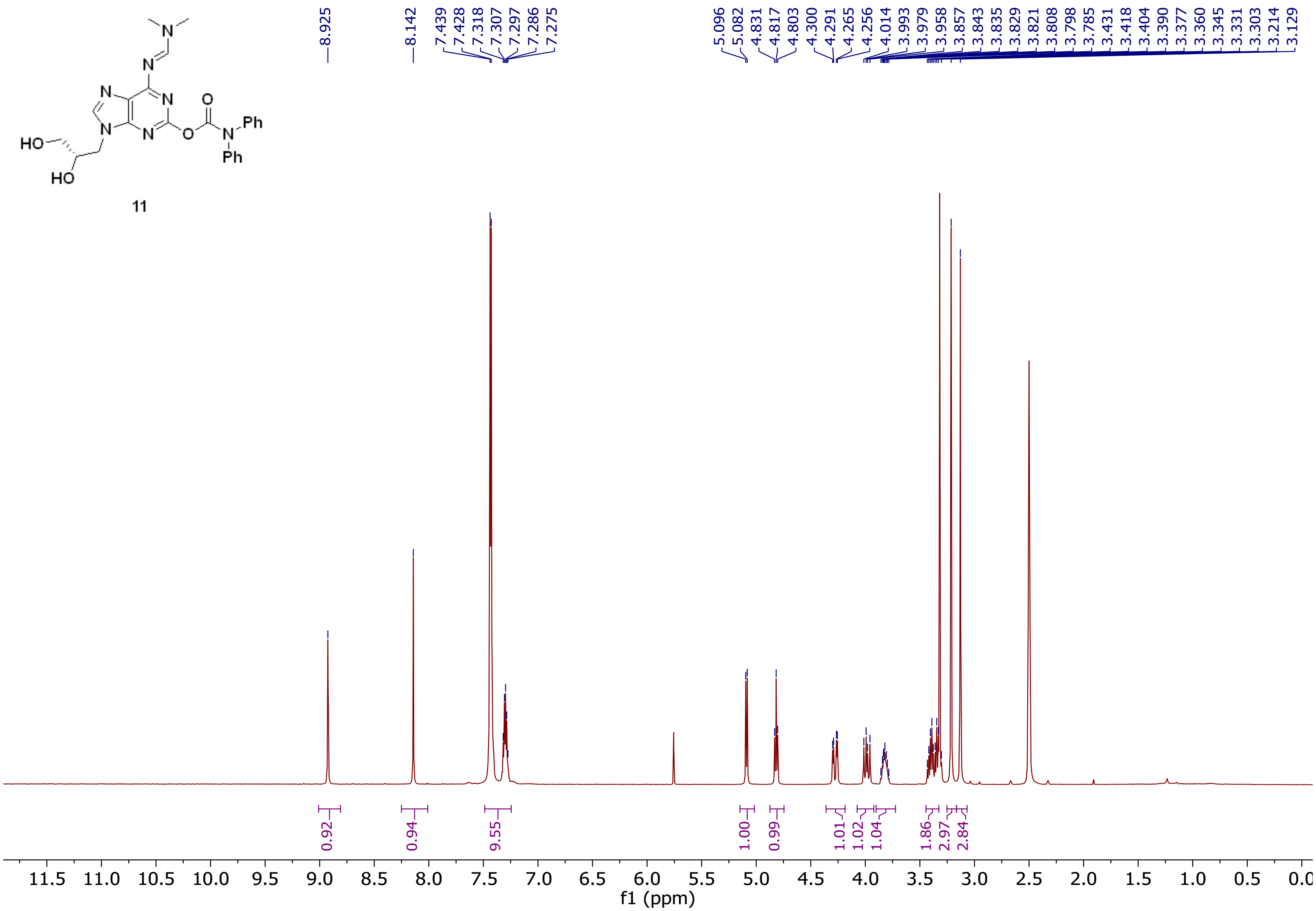

^{13}C NMR spectrum of compound **11** (126 MHz, DMSO-d_6)

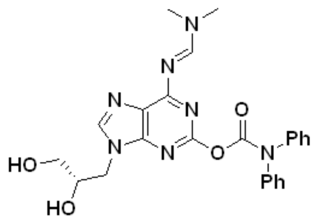

11

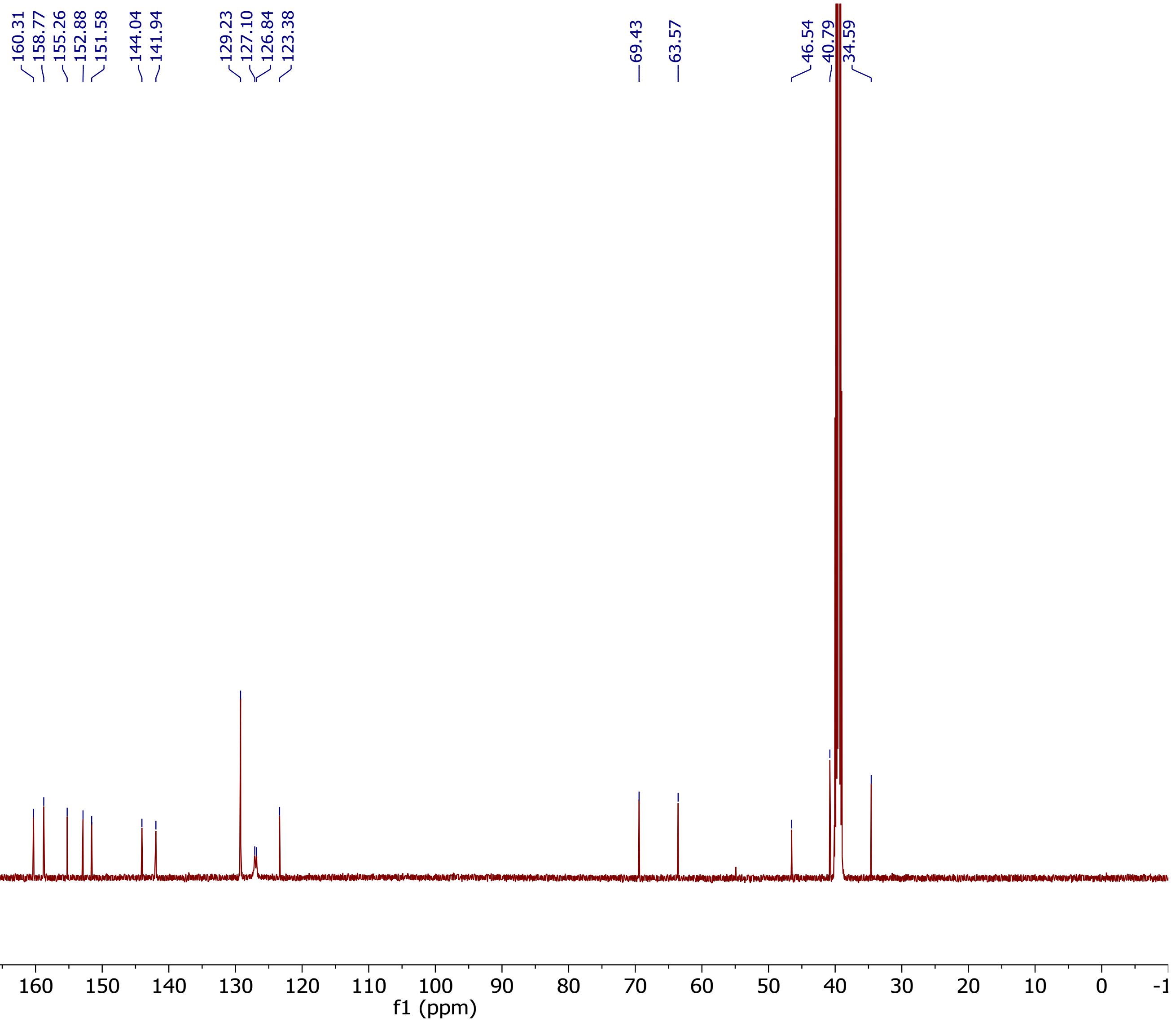

^{1}H NMR spectrum of compound **12** (400 MHz, DMSO-d_6)

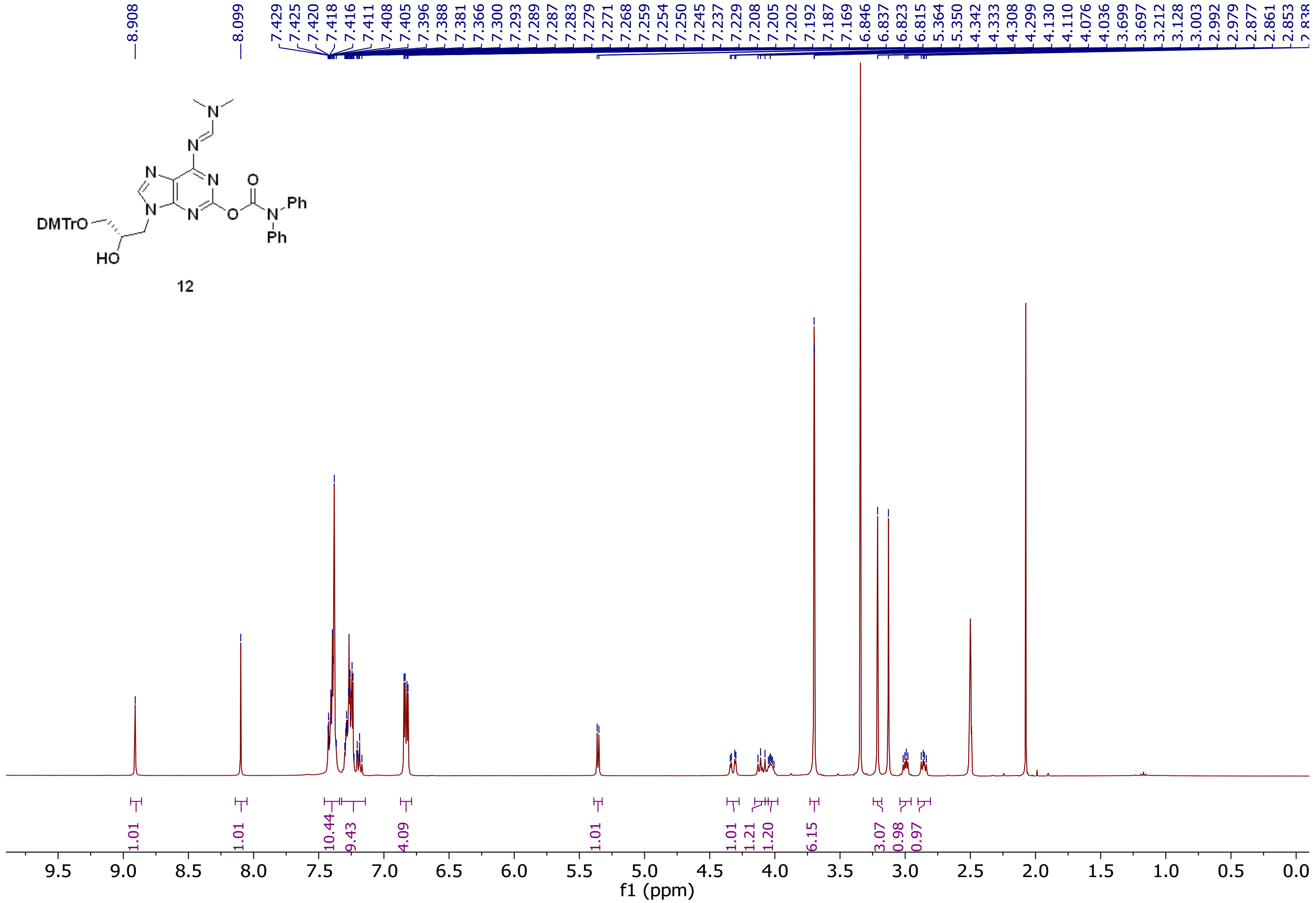

^{13}C NMR spectrum of compound **12** (101 MHz, DMSO-d_6)

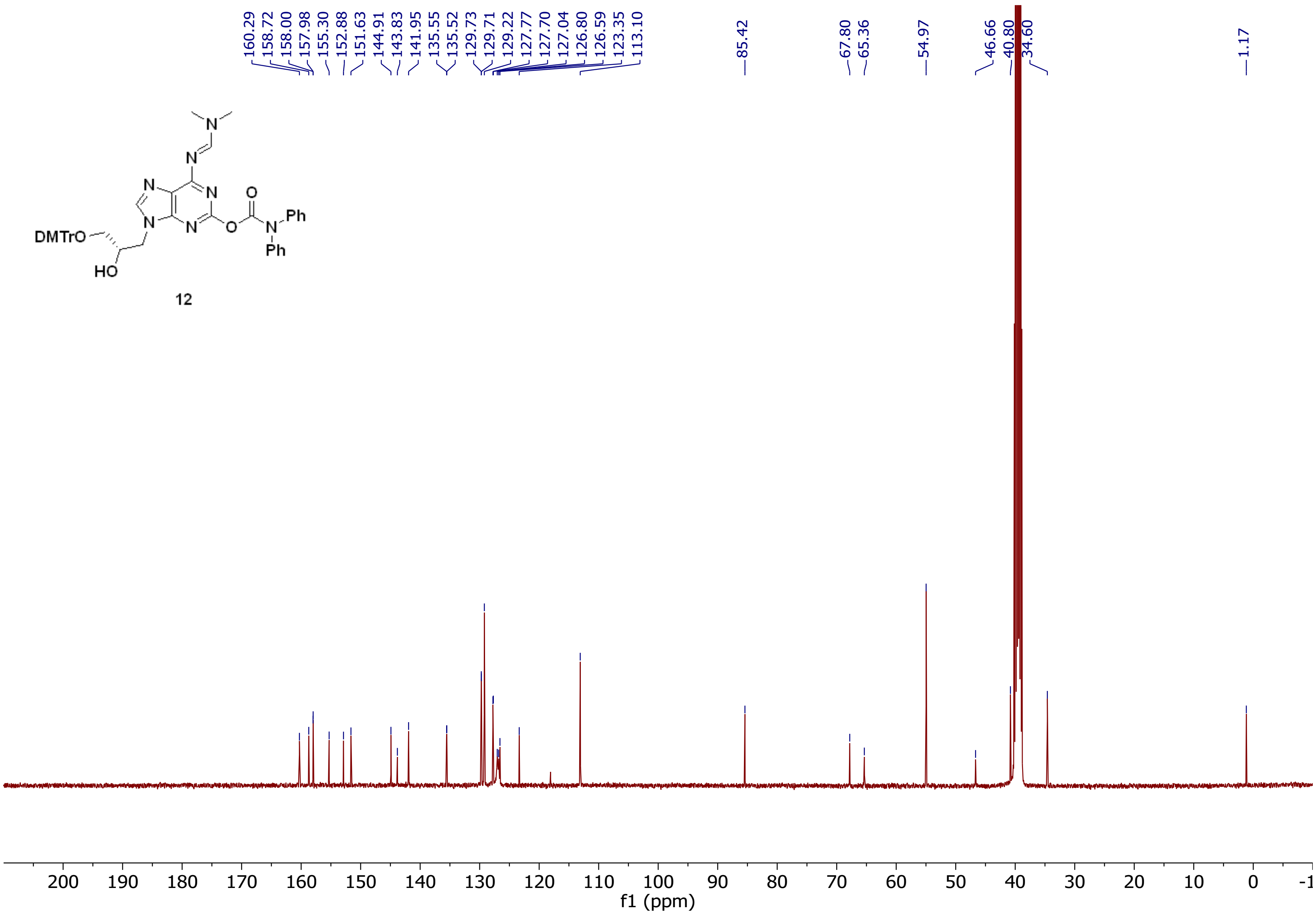

^{1}H NMR spectrum of phosphoramidite ***i*G** (500 MHz, CD_3CN)

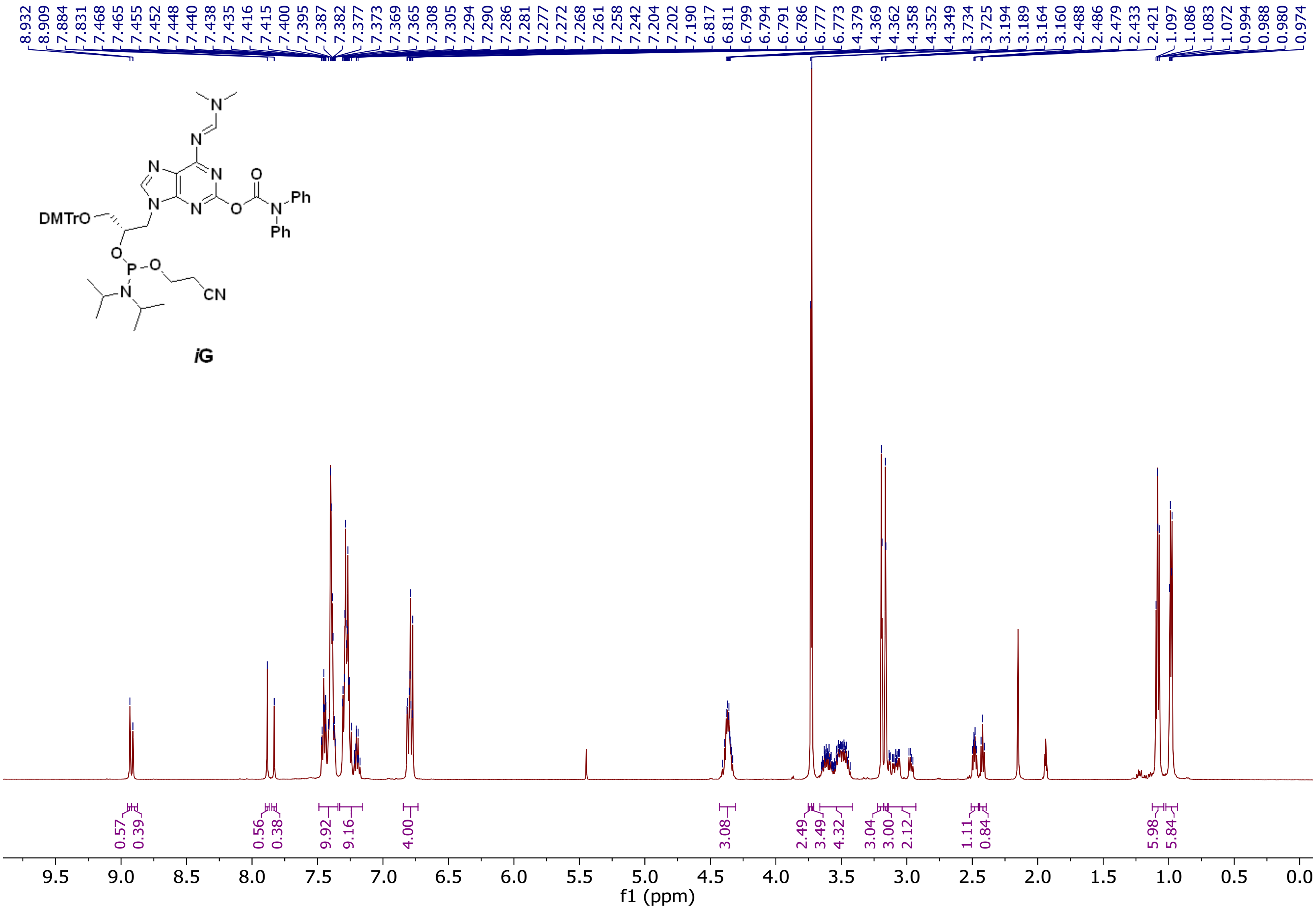

^{13}C NMR spectrum of phosphoramidite ***iG*** (126 MHz, CD_3CN)

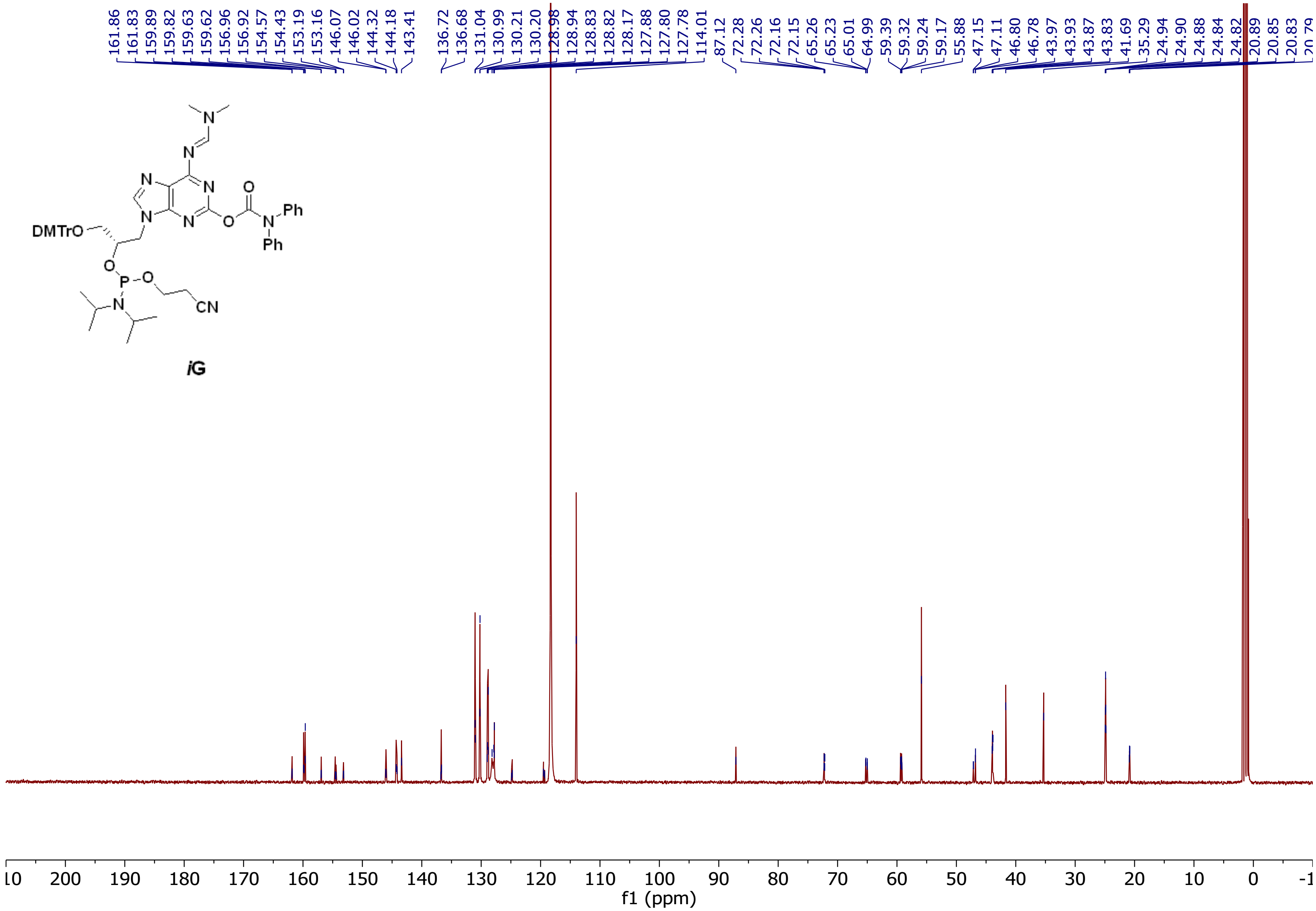

^{31}P NMR spectrum of phosphoramidite ***i*G** (202 MHz, CD_3CN)

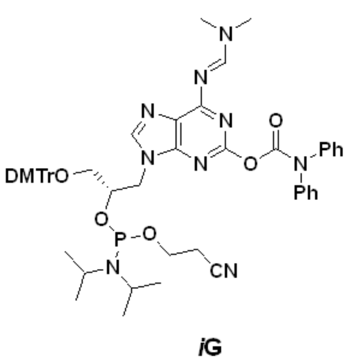

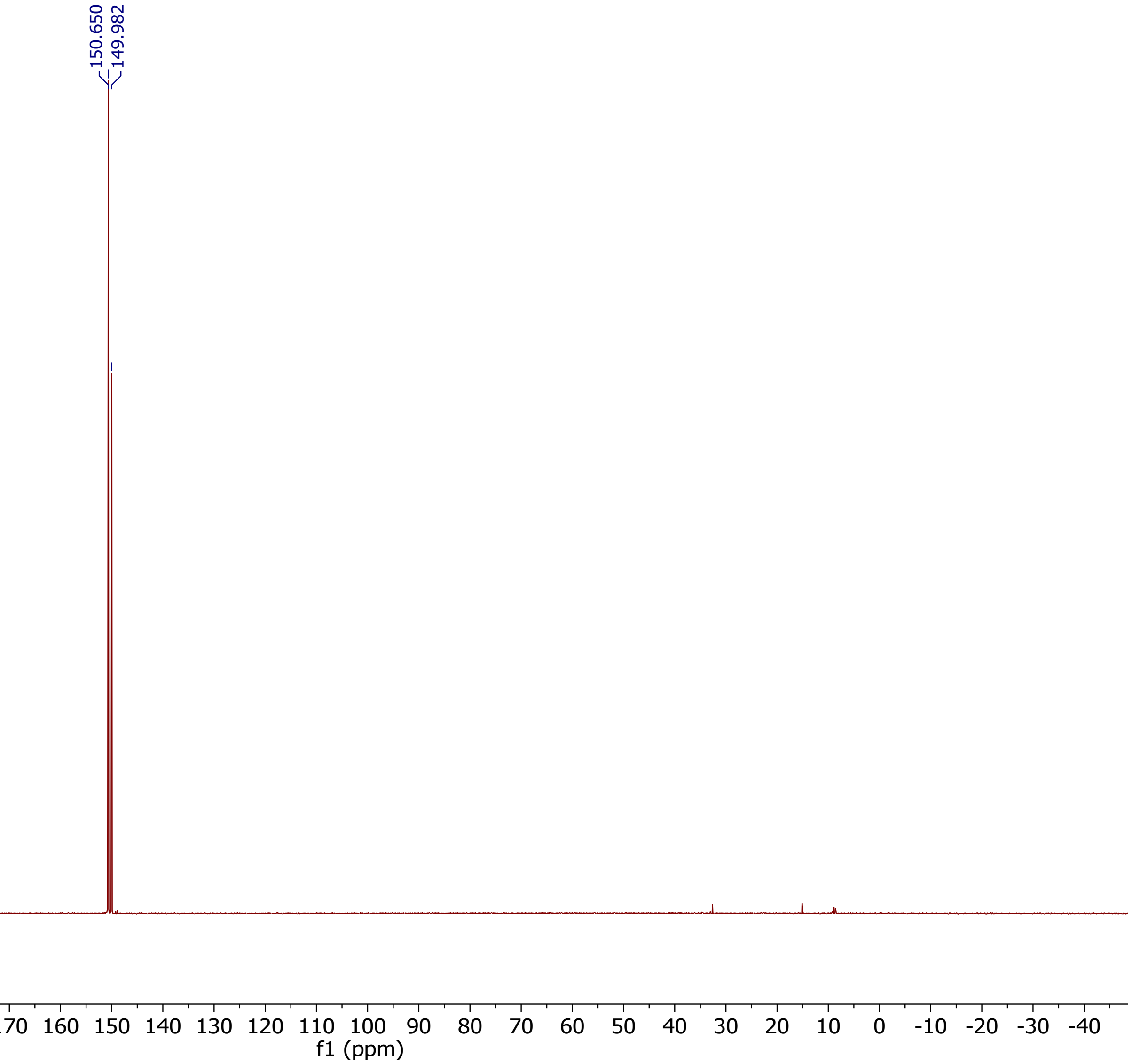

Supplement: gkab916_Supplemental_File [file gkab916_supplemental_file.pdf]
